# Swimming alleviates myocardial fibrosis of type II diabetic rats through activating miR-34a-mediated SIRT1/PGC-1α/FNDC5 signal pathway

**DOI:** 10.1371/journal.pone.0310136

**Published:** 2024-09-09

**Authors:** Yanju Guo, Fengmin Zhou, Jingjing Fan, Tong Wu, Shaohui Jia, Jinxiu Li, Ning Chen

**Affiliations:** 1 Tianjiu Research and Development Centre for Exercise Nutrition and Foods, Hubei Key Laboratory of Exercise Training and Monitoring, College of Sports Medicine, Wuhan Sports University, Wuhan, China; 2 College of Physical Education and Health, Guangxi Normal University, Guilin, China; Jordan University of Science and Technology Faculty of Medicine, JORDAN

## Abstract

Myocardial fibrosis can trigger heart failure in diabetic cardiomyopathy (DCM), and irisin, an exercise-induced myokine, may have a beneficial effect on cardiac function. However, the specific molecular mechanism between exercise and irisin in the diabetic heart remains not fully explored. This study aimed to investigate how miR-34a mediates exercise-induced irisin to ameliorate myocardial fibrosis and its underlying mechanisms. Type 2 diabetes mellitus (T2DM) with DCM was induced in adult male rats with high-fat diet and streptozotocin injection. The DCM rats were subjected to swimming (60 min/d) and recombinant irisin (r-irisin, 500 μg/kg/d) interventions for 8 weeks, respectively. Cardiac function, cardiomyocyte structure, myocardial fibrosis and its correlated gene and protein expression were analyzed. Swimming intervention alleviated insulin resistance, myocardial fibrosis, and myocardial hypertrophy, and promoted blood glucose homeostasis in T2DM model rats. This improvement was associated with irisin upregulation and miR-34a downregulation in the myocardium, thus enhancing cardiac function. Similar efficacy was observed via intraperitoneal injection of exogenous recombinant irisin. Inhibition of miR-34a *in vivo* exhibited an anti-myocardial fibrotic effect by promoting irisin secretion through activating sirtuin 1 (SIRT1)/peroxisome proliferator-activated receptor-gamma coactivator-1α (PGC-1α)/fibronectin type III domain-containing protein 5 (FNDC5) signal pathway and downregulating myocardial fibrosis markers (collagen I, collagen III, and transforming growth factor-β1). Therefore, swimming-induced irisin has the potential therapeutic effect on diabetic myocardial fibrosis through activating the miR-34a-mediated SIRT1/PGC-1α/FNDC5 signal pathway.

## 1. Introduction

Type 2 diabetes mellitus (T2DM) is a systemic metabolic disease characterized by insulin resistance and insufficient insulin secretion, accounting for more than 90% of all diabetes cases [1, 2]. According to the estimation, approximately 537 million adults (aged 20–79 years old) were diagnosed with diabetes in 2021, and this number is predicted to rise to 643 million by 2030 [3]. Diabetic cardiomyopathy (DCM) is a major factor responsible for high mortality and morbidity rates to diabetes. DCM represents a distinct disease entity, characterized by impaired metabolism, myocardial hypertrophy and fibrosis, early diastolic dysfunction, and late systolic dysfunction, accompanied by higher expression of inflammatory cytokines [4–6]. Myocardial fibrosis, characterized by excessive collagen deposition in the extracellular matrix (ECM), is one of the major pathological features of DCM, causing systolic dysfunction and heart failure [7]. Therefore, developing anti-fibrotic strategies is crucial for preventing and treating DCM.

MicroRNAs (miRNAs) are endogenous small non-coding RNA molecules with crucial roles in various biological processes, including development, regeneration, aging, and the onset and progression of many cardiovascular diseases. Several miRNAs have been identified to play an important role in the macrovascular and microvascular complications associated with T2DM [8]. MicroRNA-34 (miR-34) family members, including miR-34a, miR-34b, and miR-34c, are activated during myocardial injury [9]. MiR-34a, primarily expressed in the heart, can accelerate the senescence of endothelial and vascular smooth muscle in the vascular wall, mainly through the direct downregulation of its target gene sirtuin1 (*Sirt1*), which promotes arterial inflammation and the development of cardiovascular diseases associated with aging [10–12]. *In vivo* and *in vitro* studies have demonstrated that miR-34a promotes myocardial fibrosis in mice after myocardial infarction (MI) by targeting mothers against decapentaplegic homolog 4 (*Smad4*) [13]. Similarly, miR-34a inhibitors can suppress transforming growth factor-β1 (*Tgfb1*)-induced fibroblast proliferation and ECM deposition by targeting c-Ski *in vitro* [14]. Furthermore, miR-34a inhibitors can also mitigate hyperglycemia-induced damage to mesenchymal stem cells (MSCs), and the transplantation of anti-miR-34a-MSCs can improve left ventricular ejection fraction (LVEF) after MI, and reduce myocardial fibrosis and the infarct size in rats with T2DM induced by a high-fat diet (HFD) combined with streptozotocin (STZ) injection [15]. This suggests that targeting miR-34a may hold a potential for treating T2DM cardiomyopathy.

Irisin is a multifunctional hormone-like bioactive peptide that is mainly released from the skeletal muscle and myocardium during exercise, cold stimulation, ischemia, and inflammation [16]. Irisin secretion is triggered by activation of peroxisome proliferator-activated receptor gamma coactivator-1 alpha (PGC-1α) [17], which shows promising therapeutic potential in metabolic diseases such as obesity [18, 19], insulin resistance [20, 21], and diabetes [22, 23]. Irisin has also been found to protect the myocardium from ischemia-reperfusion injury by inhibiting the proliferation of endothelial precursors via the activation of AMP-activated protein kinase-protein kinase B-endothelial nitric oxide synthase-nitric oxide (AMPK-AKT-eNOS-NO) signaling [24, 25]. Irisin also can reduce myocardial apoptosis and hypertrophy caused by pressure overload [26]. Low-dose administration of human recombinant irisin (r-irisin) can reduce myocardial fibrosis and left ventricular function in diabetic mice, while high-dose r-irisin can stimulate mitogen-activated protein kinase (MAPK) signaling, thus upregulating matrix metalloproteinases (MMPs) in response to high-glucose environment, ultimately promoting the proliferation and migration of cardiac fibroblasts, and leading to excessive collagen deposition [27]. However, the exact mechanism of irisin for alleviating myocardial fibrosis, especially in DCM, remains unclear. Therefore, further studies are required to determine whether irisin could be a potential therapeutic target for ameliorating diabetic myocardial fibrosis.

Exercise is a non-pharmacological intervention strategy for T2DM individuals, although the underlying mechanisms remain unknown. Different forms of exercise (aerobic exercise, resistance exercise or high intensity intermittent exercise) appear to have beneficial effects on diabetic myocardial impairments, such as inhibiting pathological cardiac remodeling, reducing myocardial fibrosis, and promoting cardiac function [28–30]. The anti-remodeling effect of appropriate exercise training has been confirmed in models of heart failure, MI, and diabetes. MiR-34a is significantly upregulated in the diabetic myocardium, and exercise can trigger the generation and secretion of irisin for cardioprotection. To elucidate the underlying mechanisms, we investigated the effect and mechanism of exercise-induced irisin on myocardial fibrosis in DCM using a T2DM rat model. We hypothesized that swimming (a form of aerobic exercise) may alleviate myocardial fibrosis in diabetic rats by inducing irisin through activating miR-34a-mediated SIRT1/PGC-1α/FNDC5 signal pathway. These findings could provide experimental evidence for ameliorating the onset and progression of DCM upon swimming intervention, and a novel strategy for the development of myokine irisin as an exercise mimetic.

## 2. Materials and methods

### 2.1. Animal grouping and interventions

All animal experimental protocols were reviewed and approved by the Animal Care and Use Committee of Wuhan Sports University (S0087-20220315-04). The animals were housed under specific pathogen-free (SPF) conditions with standard lighting (12 h light/dark cycle), temperature (20–22°C), and humidity (50–60%). First, to explore the ameliorative effect of swimming-induced and exogenous recombinant irisin on DCM, 70 male Sprague-Dawley (SD) rats (4-week-old, 150 ± 10 g; Certificate No. 42010200005338; Liaoning Changsheng Biotechnology Co., Ltd) were randomly divided into normal control (NC, n = 10) and HFD (n = 60) groups. The HFD with calorie of 4.12 kcal/g comprised 50.1% carbohydrate, 13.4% protein, and 36.5% fat. The rats from the NC group were fed a standard chow diet with a total calorie of 3.04 kcal/g, comprising 64.2% carbohydrate, 23.8% protein, and 12% fat. After 4-week feeding, the rats in the HFD group were intraperitoneally (i.p.) injected with STZ (Sigma-Aldrich, MO, USA) at a dose of 30 mg/kg in 0.1 M sodium citrate buffer (pH 4.5). At two weeks after STZ injection, fasting blood glucose (FBG) was measured using a glucometer (Roche, ACCU-CHEK, Ireland) with blood collected from the tail vein. Rats with FBG levels ≥ 11.1 mmol/L and showing symptoms of polydipsia, polyuria, and polydipsia were considered T2DM model rats, which were further divided into three groups: diabetes model (DM, n = 10), diabetes swimming exercise (DE, n = 10), and diabetes r-irisin injection at 500 μg/kg body weight (BW) (DI, n = 10). These T2DM model rats were then fed an HFD for 4 additional weeks. Rats in the NC group were intraperitoneally injected with an equal volume of citrate buffer.

A 75 × 115 cm animal swimming pool (Beijing Zhongshi Dichuang, Beijing, China) was used for the swimming intervention. The pool was filled with water in a height of 40–45 cm at 30–32°C, which was used for 8-week swimming training of the rats. At the beginning of swimming training, the rats from the DE group underwent a 1-week incremental-load swimming. Sequentially, the rats in the DE group underwent swimming training (60 min/d, 5 d/week) for 8 consecutive weeks. In addition, rats in the DI group were administered with r-irisin (500 μg/kg BW) via intraperitoneal injection once a day (5 times/week) for 8 consecutive weeks. All rats from the NC and DM groups were fed their respective diets until the end of the experiments.

**Fig 1:**
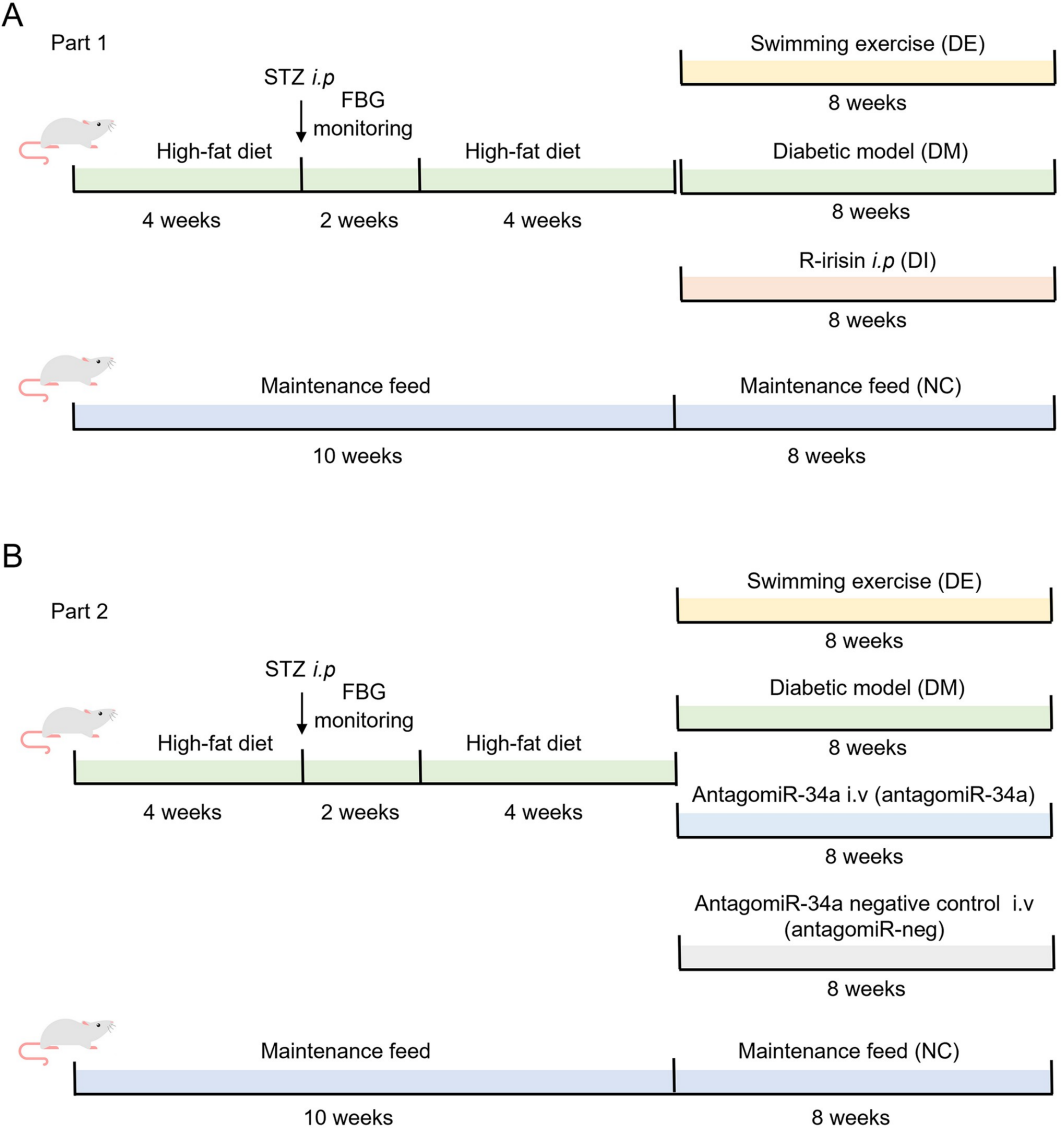
Procedures of the animal experiments. (**A**) Animal grouping and timeline for swimming and r-irisin interventions. (**B**) Animal grouping and timeline for swimming and miR-34a interventions.

To elucidate the effects of miR-34a and irisin on DCM during the swimming intervention, 82 male SD rats (4-week-old, 130 ± 20 g; Certificate No. 42010200005338; Hunan SJA Laboratory Animal Co., Ltd) were randomly divided into five groups: NC (n = 10), DM (n = 18), DE (n = 18), diabetes antagomiR-34a injection (antagomiR-34a, n = 18) and diabetes antagomiR negative control (antagomiR-neg, n = 18) groups. The T2DM rat model and swimming training scheme were same as those described above. Rats in the antagomiR-34a and antagomiR-neg groups were administered with antagomiR-34a (miR30000815, Guangzhou RiboBio Co., Ltd.) or antagomiR-neg (miR3N0000001, Guangzhou RiboBio Co., Ltd.) via tail vein injection once a week at the dose of 100 nmol in 0.2 mL of saline for 8 consecutive weeks according to the manufacturer’s instructions. Fig 1 shows the procedures of the animal experiments.

### 2.2. Body weight and blood glucose monitoring

The BW and FBG of all rats were measured at six time points: before STZ injection, at the beginning of swimming training (0 week), and during swimming training for 2, 4, 6, and 8 weeks. All rats were fasted for 8 h prior to measuring BW and FBG [31].

### 2.3. Oral glucose tolerance test (OGTT)

Before sacrifice, all rats were subjected to OGTT. Rats from each group were intragastrically administered with 50% glucose solution (2 g/kg) after 8 h fasting. Blood samples were collected from the tail vein and blood glucose (BG) was measured at 0, 30, 60, 90, and 120 min. The area under the curve (AUC) of blood glucose was then calculated using the following equation [32]:

$$\begin{array}{l} \text{AUC}=0.5\times \left({\text{BG}}_{0\text{min}}+{\text{BG}}_{30\text{min}}\right)/2+0.5\times \left({\text{BG}}_{30\text{min}}+{\text{BG}}_{60\text{min}}\right)/2+0.5\\ \times \left({\text{BG}}_{60\text{min}}+{\text{BG}}_{90\text{min}}\right)/2+0.5\times \left({\text{BG}}_{90\text{min}}+{\text{BG}}_{120\text{min}}\right)/2 \end{array}$$

### 2.4. Echocardiography

Transthoracic echocardiography was performed to evaluate cardiac function using a MyLab X5 VET ultrasound system (Esaote, Italy). The rats were continuously anesthetized with 1% isoflurane (Beijing Zhongshi Dichuang, Beijing, China) and placed on a warming pad throughout the process. Hair was removed from the target area using hair removal cream. A 22-MHz probe was placed on the body surface to measure the left ventricular end-diastolic volume (LVEDV) and left ventricular end-systolic volume (LVESV) at the level of the papillary muscle in an M-mode. The LVEF and left ventricular fraction shortening (LVFS) were calculated using the formula provided by the ultrasonic detection system. All measurements were based on the average of six consecutive cardiac cycles.

### 2.5. Tissue sample preparation

At the end of the 8-week interventions, the rats were rested for 48 h, and then narcotized with 3% sodium pentobarbital (i.p. 1.5 ml/kg BW). The blood samples were collected through abdominal aorta, and the heart from the rat was harvested to measure its weight immediately. The left ventricular tissue was cut on frozen packs, cleaned with pre-cooled saline, and fixed in 4% paraformaldehyde (Serviebio, Wuhan, China) and 2.5% glutaraldehyde (Serviebio, Wuhan, China) for immunohistochemical and transmission electron microscopic analysis, respectively. The remaining heart tissues were immediately excised, snap-frozen in liquid nitrogen, and stored at -80°C for further studies.

### 2.6. Histological and immunohistochemical staining

The left ventricular tissue was rinsed with cooled saline and fixed in 4% paraformaldehyde (Serviebio, Wuhan, China), dehydrated in alcohol, embedded in paraffin, sectioned (4 μm), and then used for HE and Masson’s trichrome staining. To evaluate the degree of myocardial injury and fibrosis, sections from three rats in each group were scanned and analyzed using a digital image analyzer. Immunohistochemical staining was performed to detect the accumulation of fibrosis-related proteins. Following antigen repair using ethylenediaminetetraacetic acid antigen repair buffer (pH 9.0) and endogenous peroxidase with a 3% hydrogen peroxide solution, and blocking of non-specific binding sites using a 3% goat serum, the sections were incubated overnight at 4°C with primary antibodies against collagen I, collagen III (1:500, Proteintech, China), or TGF-β1 (1:200, Bioss, China). After washing the sections with phosphate-buffered saline for 3 min each wash, they were incubated with a goat anti-mouse or anti-rabbit secondary antibody (Dako, Denmark) coupled with horseradish peroxidase and examined under a light microscope (BX51, Olympus, Tokyo, Japan).

### 2.7. Ultrastructure of myocardial tissues evaluated by transmission electron microscope

The myocardial tissue samples from left ventricular were fixed at a volume of 1 mm^3^, rinsed, sectioned (50 nm), and stained. The detailed experimental procedures referred to the section of materials and methods from the previous report with minor modifications [33]. The ultrastructure of myocardial tissues from left ventricular of rats in different groups was evaluated using a transmission electron microscope (HT7700 Hitachi, Tokyo, Japan) at the Research Center of Medicine and Structure Biology, Medical School of Wuhan University. Electron micrographs were randomly selected and acquired at 2500× magnification to observe the myocardial ultrastructure.

### 2.8. RNA isolation and reverse transcription-quantitative polymerase chain reaction (RT-qPCR)

Total RNA was isolated from myocardial tissues using TRIzol reagent (Sangon Biotech, Shanghai, China), and then quantified by measuring the optical density at 260 nm. Reverse transcription was performed using 1 μg RNA from each sample with the iScript cDNA synthesis kit (Bio-Rad, CA, USA) according to the manufacturer’s instructions. RT-qPCR was performed using iTaq^™^ Universal SYBR Green Supermix (Bio-Rad, Hercules, CA, USA) and the CFX Connect quantitative real-time PCR detection system (Bio-Rad Laboratories, CA, USA). The miRNA 1st Strand cDNA Synthesis Kit (Vazyme, Nanjing, China) and miRNA Universal SYBR qPCR Master Mix Kit (Vazyme, Nanjing, China) were used for determining mature miRNA expression. The β-actin was used as the housekeeping gene for target genes and U6 served as the control for miRNAs. The data were normalized using the standard comparative cycle threshold method (2^-ΔΔCT^). Table 1 shows the primer pairs used for RT-qPCR. The primers for mature miR-34a and U6 snRNA were synthesized by RiboBio Co. Ltd. (Guangzhou, China).

**Table 1:** Primers for RT-qPCR and oligonucleotides.

Gene	Sequence (5′–3′)	Accession number
***miR-34a***	F: CGCGTGGCAGTGTCTTAGCT	NR_029610
	R: AGTGCAGGGTCCGAGGTATT	
***U6***	F: CTCGCTTCGGCAGCACA	NR_138085
	R: AACGCTTCACGAATTTGCGT	
***Sirt1***	F: GAGTGTGCTGGAGGATCTG	NM_001414959.1
	R: TGCTCTGATTTGTCTGGTGT	
***Pgc1α***	F: TTCAGGAGCTGGATGGCTTG	NM_031347.1
	R: TATGTTCGCGGGCTCATTGT	
***Fndc5***	F: GAGGTGCTGATCATCGTCGT	NM_001270981
	R: GAGCAAGCACTGAAAGGGTTT	
***β-actin***	F: CCTCACTGTCCACCTTCCA	NM_031144
	R: GGGTGTAAAACGCAGCTCA	
**Oligonucleotide antagomiR-34a**	UGGCAGUGUCUUAGCUGGUUGUU	NR_029610

### 2.9. Western blotting

Myocardial tissues were lysed and homogenized in lysis buffer (Beyotime, Shanghai, China) for radioimmunoprecipitation assay, and the protein concentration was quantified using a bicinchoninic acid (BCA) protein assay kit (Beyotime, Shanghai, China). Protein samples (30 μg) were separated using sodium dodecyl sulphate-polyacrylamide gel electrophoresis (SDS-PAGE) and transferred to polyvinylidene fluoride (PVDF) membrane (Millipore, Billerica, MA, USA). After blocking with 5% non-fat milk for 1.5 h at room temperature, the membrane was incubated overnight at 4°C with primary antibodies, including SIRT1 and PGC-1α (1:1000, GeneTex Inc., CA, USA), and FNDC5 (1:1000, Abcam, Cambridge, UK). After three washes with Tris-buffered saline supplemented with Tween-20 (TBS-T) for 15 min, the membrane was incubated with rabbit horseradish peroxidase-conjugated secondary antibody (1:10000, Boster, Wuhan, China) for 1 h at room temperature. Immunoreactive protein bands were visualized using an enhanced chemiluminescence (ECL) kit (Thermo Fisher Scientific, Waltham, USA) after three washes with TBS-T for 10 min. The ChemiScope6300 imaging system (CLiNX Science Instruments, Shanghai, China) was used to acquire images. The integrated density of the blots for the target protein was quantified using ImageJ software (NIH, Bethesda, MD, USA) and presented as a relative expression of the target protein to the internal control tubulin from at least three rats in each group.

### 2.10. Statistical analysis

All data were presented as mean ± standard deviation (M ± SD) and conducted corresponding statistical analysis by GraphPad Prism version 9.0 software (La Jolla, CA, USA) and SPSS version 22.0 (Chicago, USA). The data of FBG and blood glucose AUC were analyzed using two-way repeated measure ANOVA, including the effect of time, group, and the interaction between group and time; and the Bonferroni adjustment for post-hoc comparisons following ANOVA. Group comparisons were examined using one-way ANOVA followed by Tukey’s test. The statistically significant difference was considered at *P* < 0.05.

## 3. Results

### 3.1. Swimming and r-irisin reduced FBG, glucose tolerance, and insulin resistance of T2DM rats

**Fig 2:**
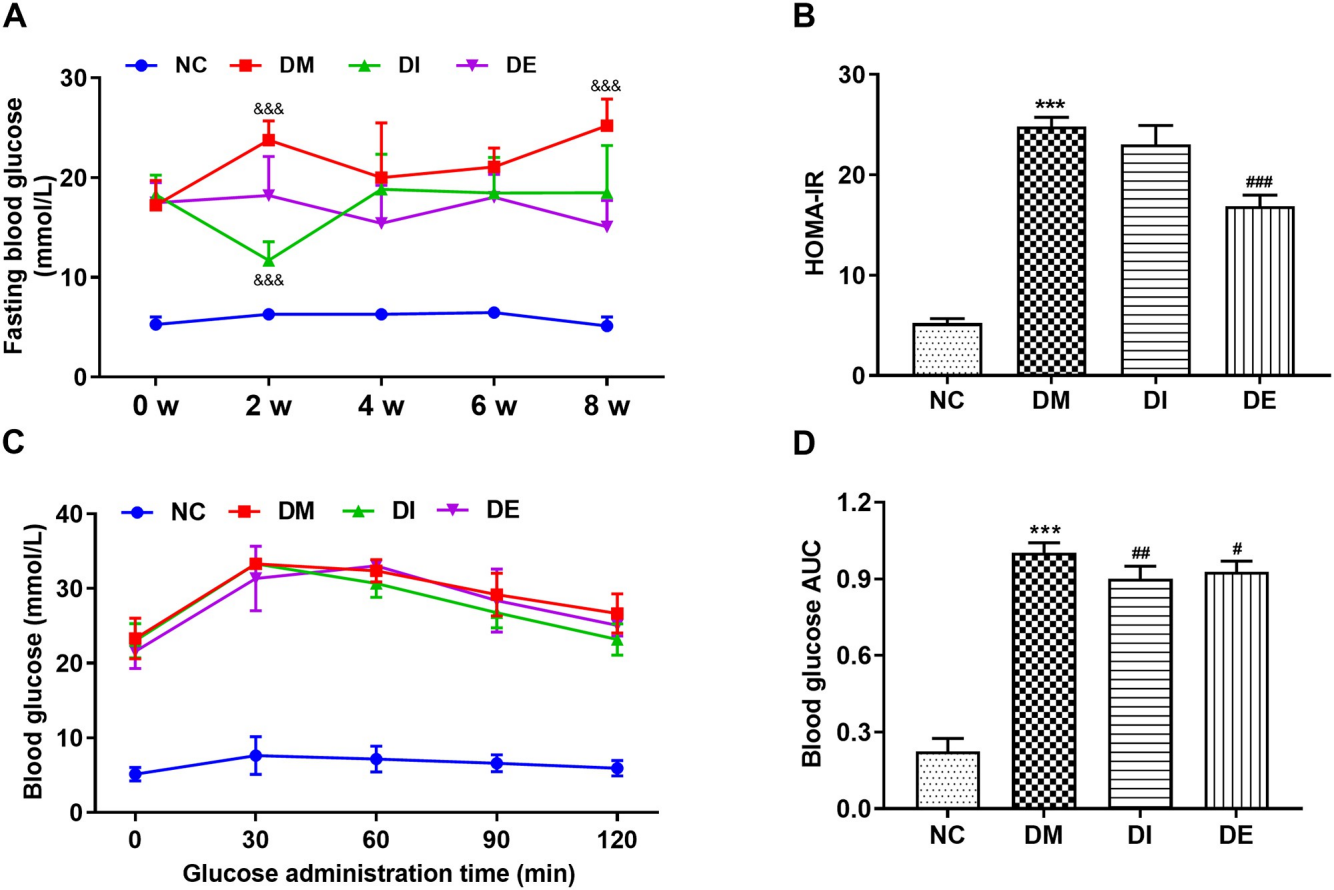
FBG (A) during intervention, and HOMA-IR (B), blood glucose (C) and blood glucose AUC (D) during OGTT for rats from each group. All data are expressed as mean ± standard deviation (M ± SD) (n = 8 rats per group). ^&&&^*P* < 0.001 versus at time point of 0 week within group; ***P < 0.001 versus NC group; ^#^*P* < 0.05, and ^###^*P* < 0.001 versus DM group. NC, normal control; DM, diabetic model; DI, diabetic-r-irisin; DE, diabetic-swimming exercise.

The FBG of rats in the DM group exhibited an obvious increase with the prolongation of the time course, and the FBG level at the 8^th^ week was significantly increased when compared with that of at 0 week (*P* < 0.01). After 8-week swimming intervention, the FBG level of rats in the DE group decreased from 17.48 mmol/L at 0 week to 15.06 mmol/L at the 8^th^ week, but there was no significant difference. During the intervention of i-irisin injection, the FBG levels of the rats from the DI group decreased first and then increased to the level at 0 week. However, after 8 weeks of swimming and r-irisin interventions, the rats from DI and DE groups exhibited a significant decrease in FBG when compared with that of DM rats (Fig 2A). Based on the above results, FBG levels revealed significant differences between groups (*P* < 0.01), and the interaction effect between group and time was also significant (*P* < 0.01).

The homeostasis model assessment of insulin resistance (HOMA-IR) level of rats in the DM group was significantly elevated when compared with that of rats in the NC group. After 8 weeks of swimming intervention, the HOMA-IR level of rats in the DE group was significantly decreased, whereas no significant change was found in the DI group (Fig 2B). The blood glucose AUC during OGTT was significantly increased for rats in the DM group when compared with that of the NC group. However, after 8 weeks of intervention, the blood glucose AUC of the rats from DI and DE groups decreased significantly when compared with that in the DM group (Fig 2C and 2D). Therefore, swimming and exogenous r-irisin interventions could partially rescue impaired islet function and alleviate insulin resistance in T2DM rats, and the hypoglycemic effect of swimming seems to be better than that of r-irisin.

### 3.2. Swimming and r-irisin rescued left ventricular dysfunction and remodeling

**Fig 3:**
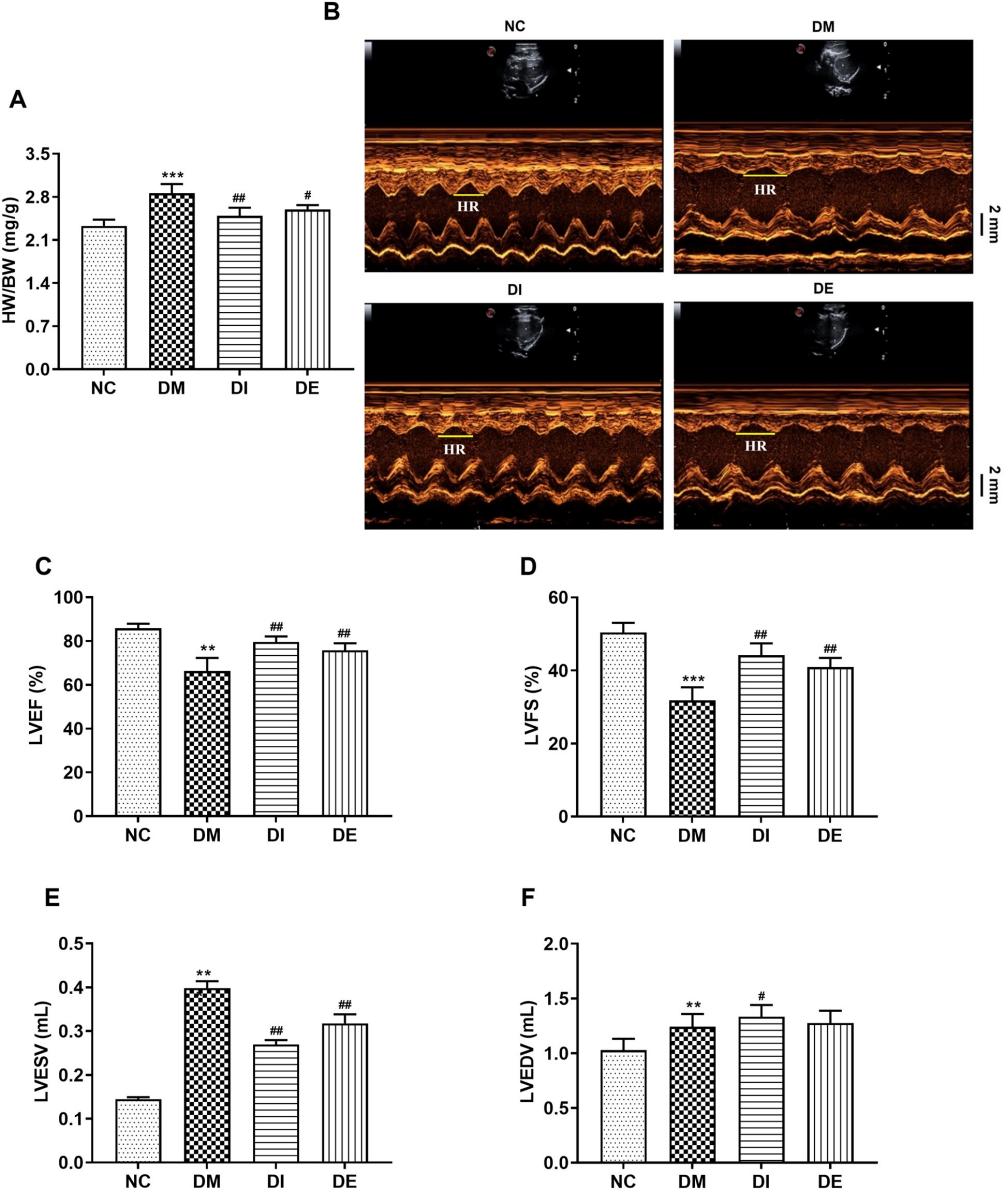
Swimming and r-irisin interventions inhibited myocardial hypertrophy and dysfunction in T2DM rats. **(A)** Heart weight/body weight (HW/BW) ratio; **(B)** Representative echocardiographic images of left ventricular (Scale bar: 2 mm); **(C)** Left ventricular ejection fraction (LVEF); **(D)** Left ventricular fractional shortening (LVFS); **(E)** Left ventricular end-systolic volume (LVESV) and **(F)** Left ventricular end-diastolic volume (LVEDV). All data are expressed as mean ± standard deviation (M ± SD) (n = 5 rats per group). **P < 0.01, ***P < 0.001 versus NC group; ^#^*P* < 0.05, ^##^*P* < 0.01 versus DM group. NC, normal control; DM, diabetic model; DI, diabetic-r-irisin; DE, diabetic-swimming exercise.

The ratio of heart weight (HW) to BW serves as an indicator of myocardial hypertrophy [34]. The HW/BW ratios of the rats in the DM group were significantly increased when compared with that in the NC group. However, after 8 weeks of swimming and r-irisin interventions, the HW/BW ratios of the rats in the DE and DI groups were significantly lower than those of rats in the DM group (Fig 3A). Based on the parameter analysis of ultrasonic images for the hearts from all rats (Fig 3B), compared with the NC group, the rats from the DM group exhibited significantly reduced LVEF and LVFS. However, after 8 weeks of swimming and r-irisin interventions, LVEF and LVFS were significantly improved in rats from DE and DI groups (Fig 3C and 3D). In addition, the rats from the DM group exhibited marked increase in LVESV and LVEDV when compared with those of rats from the NC group. Swimming and r-irisin interventions significantly reduced LVESV in T2DM rats, and the LVEDV of the rats from the DI group was significantly higher than that of the rats from the DM group (Fig 3E and 3F). These findings suggested that swimming and r-irisin interventions have cardiac protective functions, thereby rescuing myocardial hypertrophy and dysfunction in T2DM rats.

### 3.3. Swimming and r-irisin mitigated the fibrosis and disordered pathogenic structure in the diabetic myocardium

**Fig 4:**
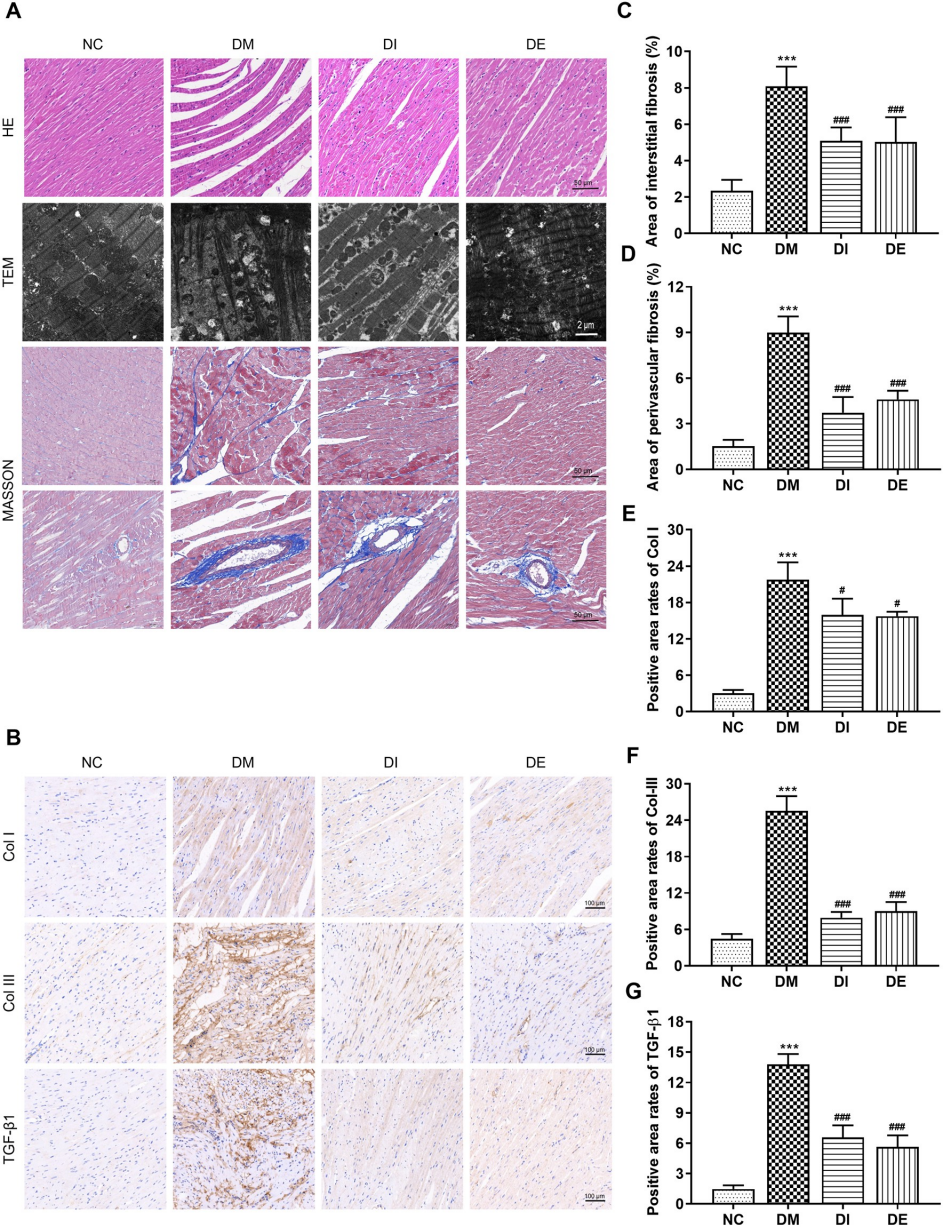
Swimming and r-irisin interventions mitigated myocardial fibrosis of T2DM rats. (**A**) Representative histopathological images stained with HE (scale bar: 50 μm), ultrastructure observed using TEM (scale bar: 2 μm), and interstitial and perivascular fibrosis stained with Masson’s trichrome (scale bar: 50 μm) of left ventricular (LV) longitudinal sections. (**B**) Representative images of immunohistochemical staining of Col I, Col III, and TGF-β1. (**C, D**) Statistical analysis for interstitial and perivascular fibrosis areas evaluated with Masson’s trichrome staining. (**E, F, G**) Quantification of Col I, Col III, and TGF-β1 expression evaluated by immunohistochemical staining. All data are presented as mean ± standard deviation (M ± SD) (n = 3 rats per group). ***P < 0.001 versus NC group; ^#^*P* < 0.05, ^###^*P* < 0.001 versus DM group. NC, normal control; DM, diabetic model; DI, diabetic-r-irisin; DE, diabetic-swimming exercise; TEM, transmission electron microscope.

HE staining of the left ventricular myocardium showed that rats in the NC group exhibited well-arranged and intact myocardial fibers, clear nuclei, and uniform nuclei and cytoplasmic staining. In contrast, the myocardial cells of the rats in the DM group showed broken and disordered myocardial fibers, an irregular nucleus size, and inflammatory cell infiltration. Rats in the DI group exhibited reduced damage to the structure of myocardial fibers, although myocyte hypertrophy was still observed. Notably, rats in the DE group exhibited more obviously weaken damage to myocardial fibers (Fig 4A). Transmission electron microscopic observation showed that cardiomyocytes of rats from the NC group had a normal myocardial structure, characterized by regular sarcomeres composed of continuous myofibrils with well-distributed mitochondria arranged in an orderly manner between myofibrils. In contrast, cardiomyocytes of rats from the DM group showed large broken myocardial fibers, the disappearance of z-lines, and vacuolation between myocardial filaments. The ablation of myocardial fibers resulted in the accumulation of many mitochondria, accompanied by ruptured mitochondrial membrane and destroyed cristae (Fig 4A). However, swimming and r-irisin interventions rescued these irregular and disordered structures.

Similarly, Masson’s trichrome staining showed that the myocardial tissues of rats in the DM group exhibited a marked increase in collagen deposition in the interstitial and perivascular areas when compared with those of rats in the NC group, which was clearly mitigated in all regions of the myocardial tissues in T2DM rats following swimming and r-irisin interventions (Fig 4A, 4C and 4D). Immunohistochemical staining further confirmed these findings, showing substantial upregulation of Col I, Col III, and TGF-β1 in the myocardium of T2DM rats when compared with corresponding expression levels in the NC group. Conversely, swimming and r-irisin interventions resulted in marked downregulation of these factors, which was consistent with results obtained from Masson staining (Fig 4B and 4E–4G).

### 3.4. Swimming and r-irisin activated the miR-34a-mediated SIRT1/PGC-1α/FNDC5 signal pathway in myocardial tissues of T2DM rats

**Fig 5:**
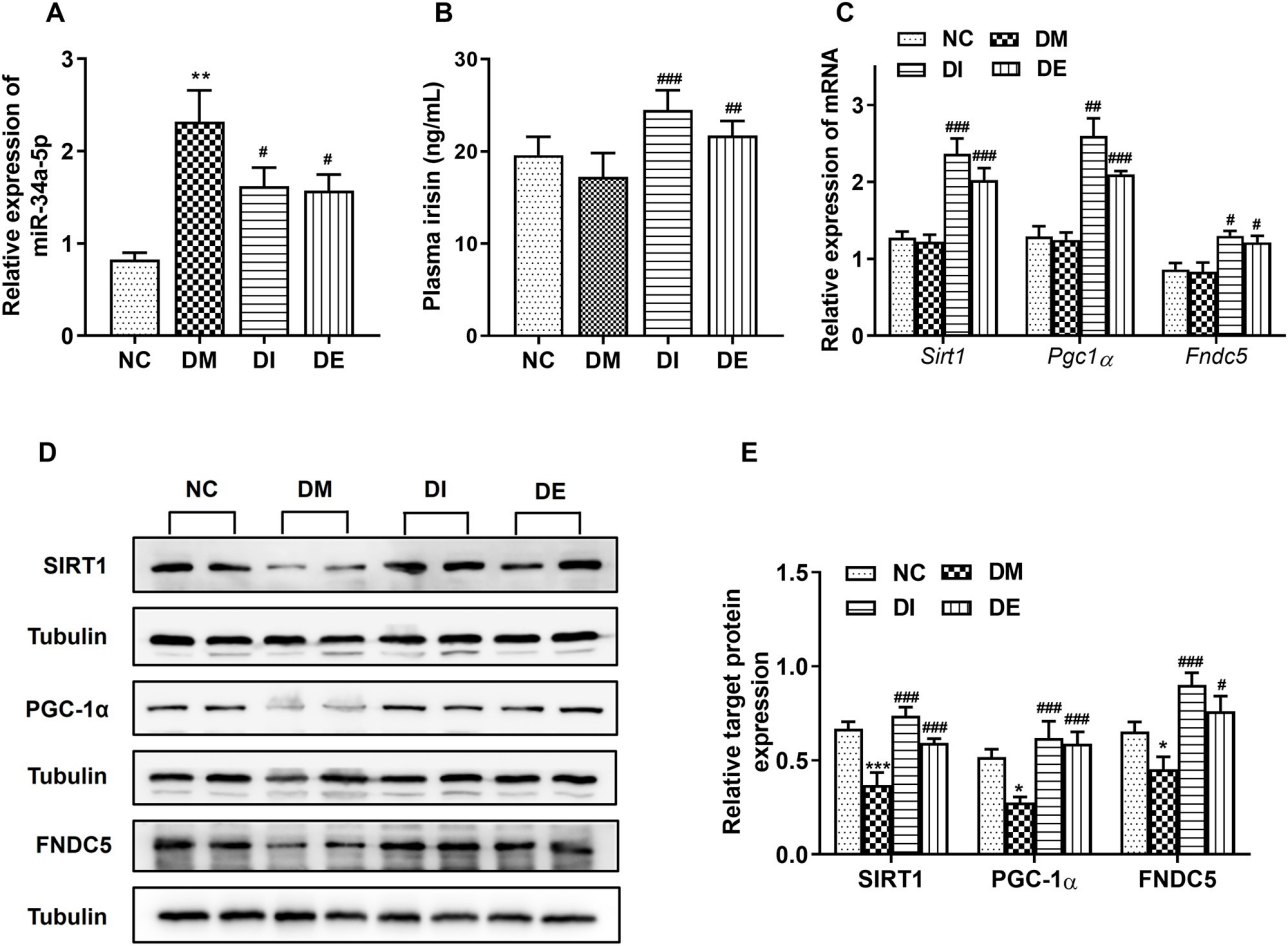
The plasma irisin levels and the expression of miR-34a, target genes and proteins. **(A)** The expression of miR-34a. **(B)** The levels of plasma irisin. **(C)** The mRNA expression of *Sirt1*, *Pgc1α*, and *Fndc5*. **(D)** Western blots for evaluating protein expression of SIRT1, PGC-1α, and FNDC5. **(E)** Corresponding statistical analysis for expression levels of SIRT1, PGC-1α, and FNDC5 proteins. All data are presented as mean ± standard deviation (M ± SD). *P < 0.05, **P < 0.01, and ***P < 0.001 versus NC group; ^#^*P* < 0.05, ^##^*P* < 0.01 and ^###^*P* < 0.001 versus DM group. NC, normal control; DM, diabetic model; DI, diabetic-r-irisin; DE, diabetic-swimming exercise.

The level of miR-34a in the myocardium of rats from the DM group was significantly higher than that in the NC group. However, after 8 weeks of swimming and r-irisin interventions, the expression of miR-34a in the myocardium of T2DM rats was significantly inhibited (Fig 5A). The plasma irisin levels of the rats from DE and DI groups were significantly increased when compared with those in the DM group (Fig 5B). Additionally, there was no significant difference in the mRNA levels of *Sirt1*, *Pgc1α*, and *Fndc5* in myocardial tissues of the rats from the DM group when compared with those in NC rats. After 8 weeks of interventions, these mRNA levels were significantly increased in the myocardial tissues of rats from DI and DE groups when compared with those from the DM group (Fig 5C). Moreover, the expression levels of SIRT1, PGC-1α and FNDC5 proteins in the myocardium of the rats in the DM group were significantly decreased when compared with the NC group. The expression levels of SIRT1, PGC-1α and FNDC5 proteins in rats from the DI and DE groups were significantly higher than those from the DM group (Fig 5D and 5E). These results demonstrated that swimming and r-irisin interventions may inhibit miR-34a expression, and activate its downstream targets, ultimately alleviating myocardial fibrosis in T2DM rats.

### 3.5. MiR-34a antagonist alleviated insulin resistance, reduced blood glucose, and rescued left ventricular dysfunction and remodeling in T2DM rats

**Fig 6:**
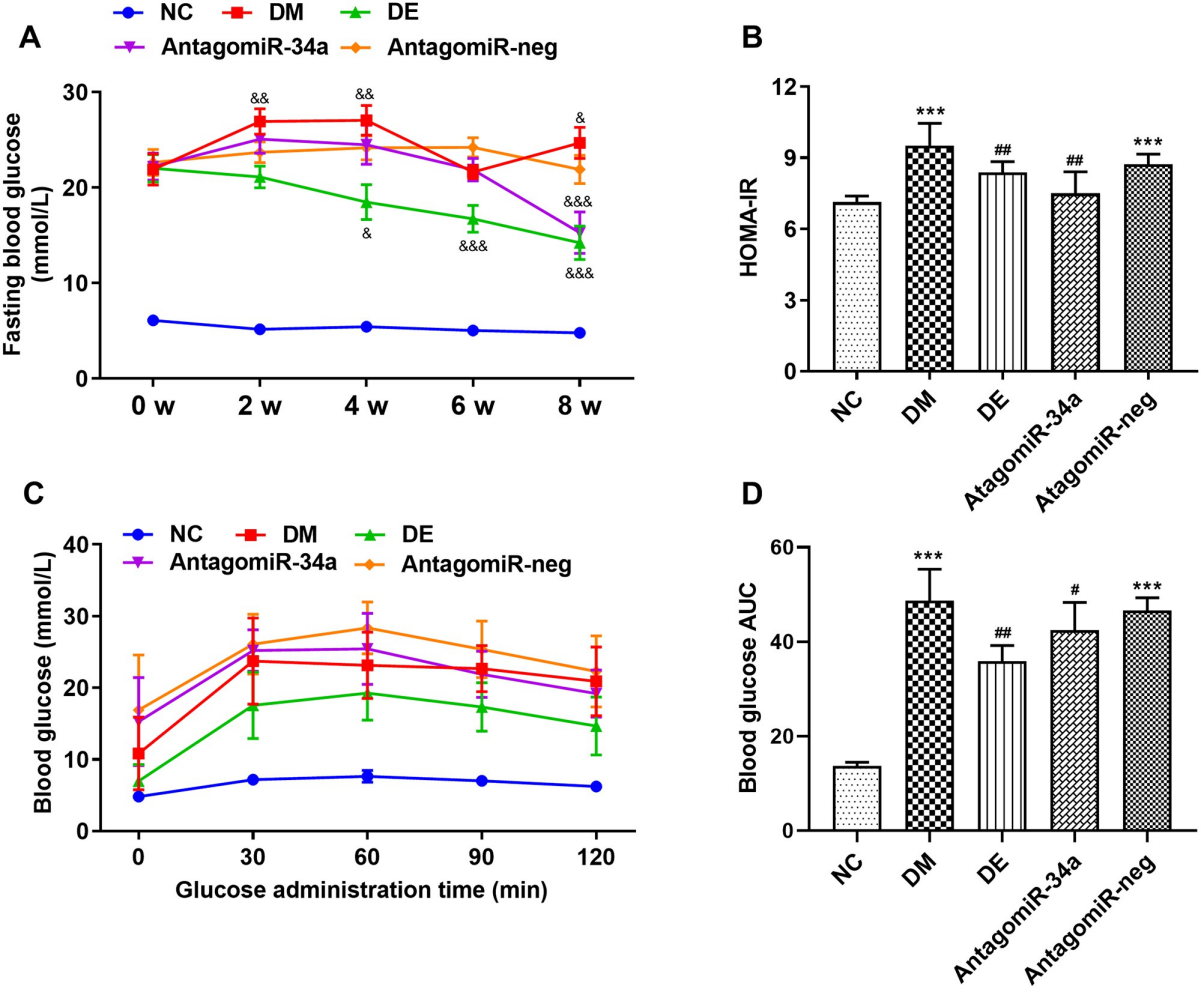
FBG (A) during intervention, and HOMA-IR (B), blood glucose (C) and blood glucose AUC (D) during OGTT for rats from each group. All data are expressed as mean ± standard deviation (M ± SD) (n = 8 rats per group). ^&^*P* < 0.05, ^&&^*P* < 0.01 and ^&&&^*P* < 0.001 versus at time point of 0 week within group; ***P < 0.001 versus NC group; ^#^*P* < 0.05 and ^##^*P* < 0.01 versus DM group. NC, normal control; DM, diabetic model; DE, diabetic-swimming exercise; antagomiR-34a, miR-34a antagonist intervention; antagomiR-neg, antagomiR negative control.

The FBG of rats in the DM group was increased with the prolongation of the time course, and the FBG level at the 8^th^ week was significantly increased when compared with that of at 0 week. The FBG levels in the DE group decreased gradually with the intervention time, and decreased significantly at the end of the 8^th^ week as compared with that before intervention. Meanwhile, the FBG levels of the rats in the antagomiR-34a group significantly decreased when compared to those at pre-intervention (Fig 6A). The HOMA-IR levels of rats in the DE and antagomiR-34a groups significantly decreased after 8-week interventions when compared with those in DM and antagomiR-neg groups (Fig 6B). The blood glucose AUC during OGTT for the rats in DM and antagomiR-neg groups was significantly higher than that of rats in the NC group; however, swimming and antagomiR-34a interventions significantly reduced blood glucose AUC (Fig 6C and 6D). The mitigation of antagomiR-34a treatment on blood glucose and insulin resistance (as measured by HOMA-IR) in diabetic rats was comparable to that of the swimming intervention. FBG levels revealed significant differences at different time points within the group (*P* < 0.001), and between groups (*P* < 0.001); similarly, the interaction effect between group and time was also significant (*P* < 0.001).

**Fig 7:**
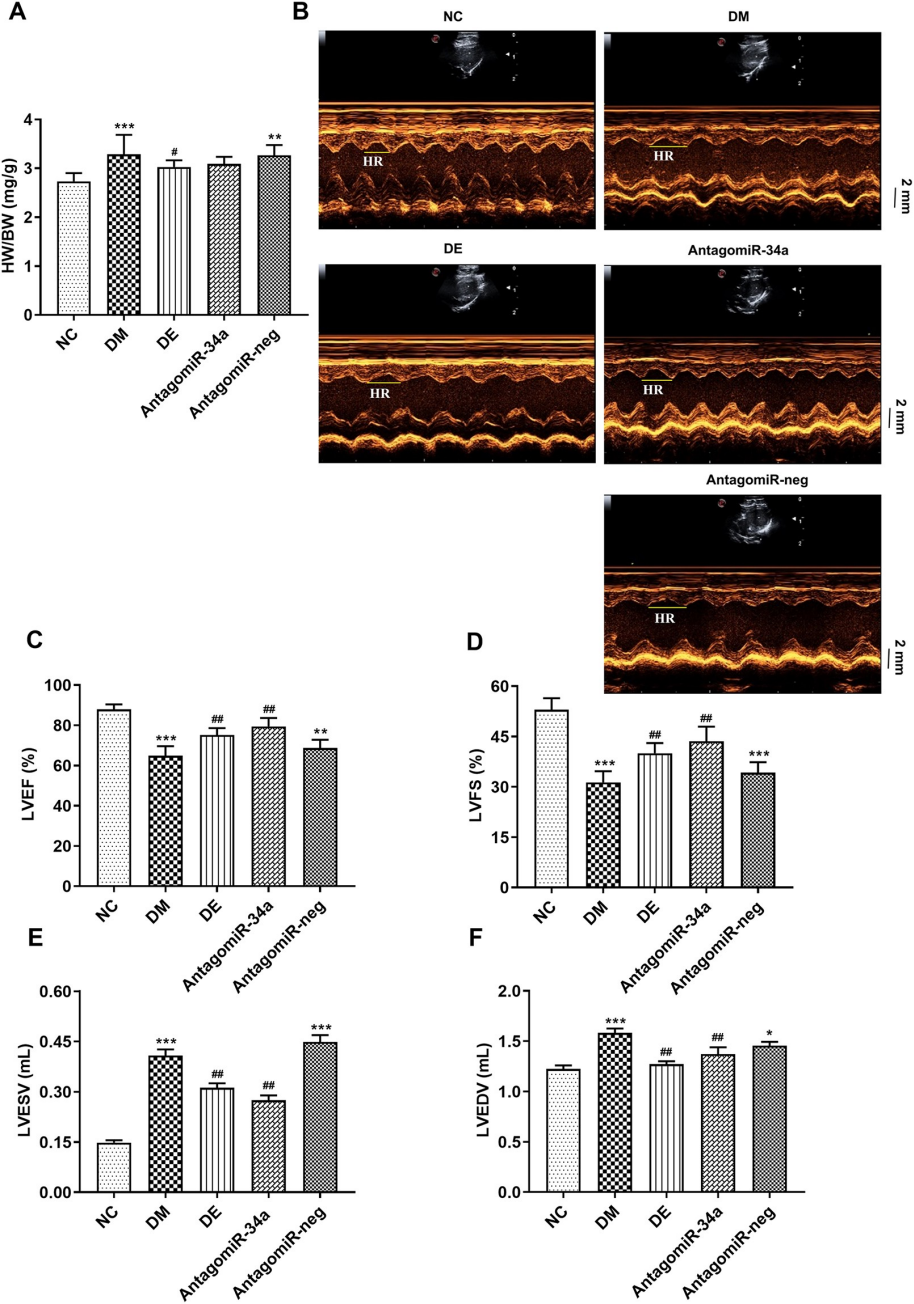
Swimming and miR-34a antagonist interventions prevented myocardial hypertrophy and dysfunction in T2DM rats. (**A**) HW/BW ratio, (**B**) Representative echocardiographic images of the left ventricle (scale bar: 2 mm), (**C**) LVEF, (**D**) LVFS, (**E**) LVESV, and (**F**) LVEDV. All data are expressed as mean ± standard deviation (M ± SD) (n = 5 rats per group). *P < 0.05, **P < 0.01, ***P < 0.001 versus NC group; ^#^*P* < 0.05, ^##^*P* < 0.01 versus DM group. NC, normal control; DM, diabetic model; DE, diabetic-swimming exercise; antagomiR-34a, miR-34a antagonist intervention; antagomiR-neg, antagomiR negative control.

The HW/BW ratio, an apparent indicator of myocardial hypertrophy, did not exhibit a significant decrease in rats from the antagomiR-34a group as compared with those from the DM and antagomiR-neg groups (Fig 7A). Based on the parameter analysis of ultrasonic images of hearts from all rats upon corresponding interventions (Fig 7B), echocardiography showed that the rats in the DM and antagomiR-neg groups exhibited a significant decrease in LVEF and LVFS, while LVEDV and LVESV increased significantly when compared with those in the NC group. Following 8 weeks of miR-34a inhibitor injection, the LVEF and LVFS of T2DM rats significantly increased, while LVEDV and LVESV markedly decreased when compared with those of rats from the DM and antagomiR-neg groups. These changes were consistent with the trends observed in the DE group (Fig 7C–7F). These findings indicated that the effect of the miR-34a antagonist on left ventricular dysfunction and remodeling in T2DM rats is similar to the swimming intervention.

### 3.6. MiR-34a antagonist alleviated myocardial fibrosis and restored cardiomyocyte structure in T2DM rats

**Fig 8:**
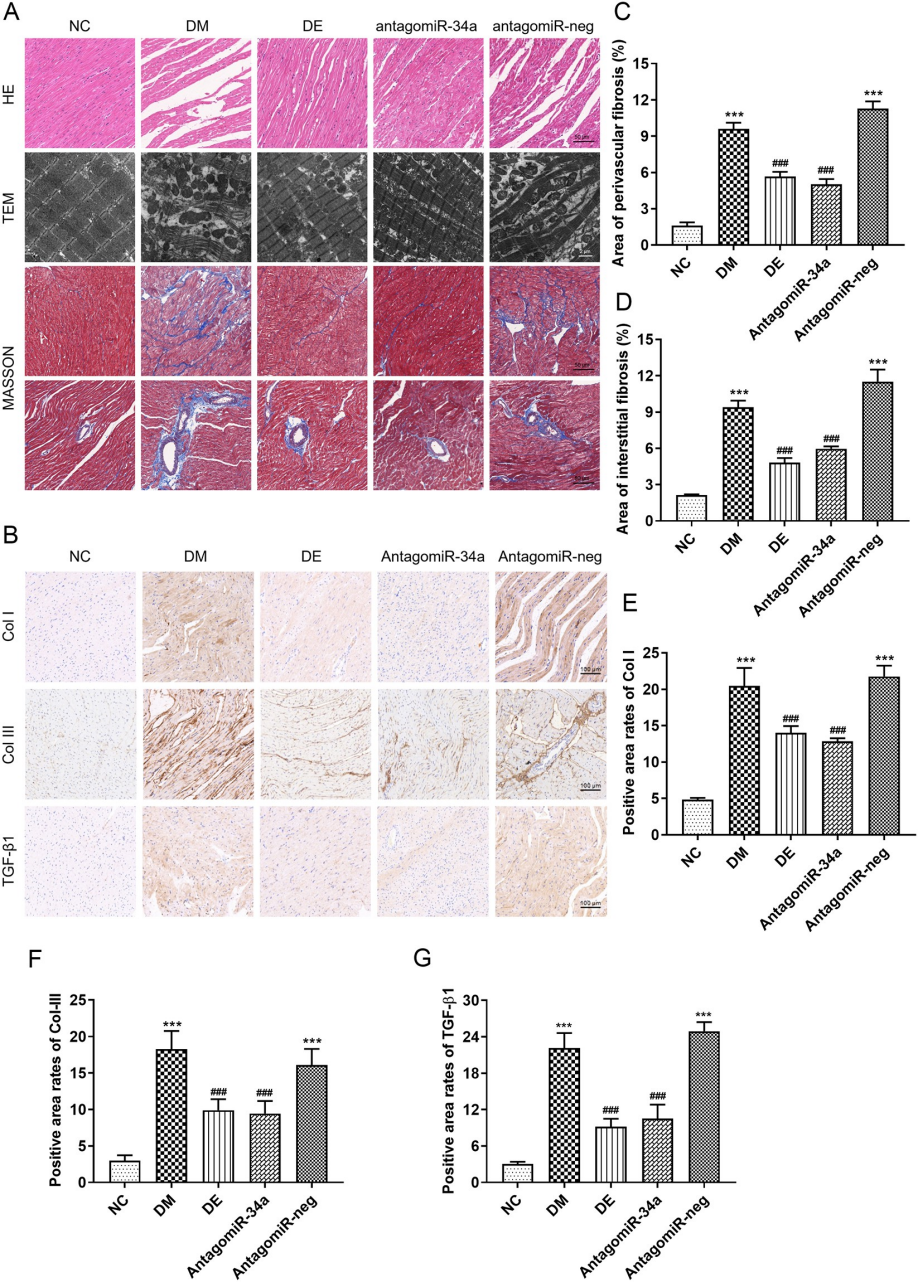
Swimming and miR-34a antagonist suppressed myocardial fibrosis of T2DM rats. (**A**) Representative HE staining images (scale bar: 50 μm), observation of the ultrastructure with TEM (scale bar: 2 μm), interstitial and perivascular fibrosis of left ventricular (LV) longitudinal sections stained with Masson’s trichrome (scale bar: 50 μm). (**B**) Representative images of immunohistochemical staining for Col I, Col III, and TGF-β1 (scale bar: 100 μm). (**C, D**) Interstitial and perivascular fibrosis area subjected to Masson’s trichrome staining. (**E, F, G**) Quantification of Col I, Col III, and TGF-β1 expression evaluated by immunohistochemical staining. All data are expressed as mean ± standard deviation (M ± SD). ***P < 0.001 versus NC group; ^###^*P* < 0.001 versus DM group. NC, normal control; DM, diabetic model; DE, diabetic-swimming exercise; antagomiR-34a, miR-34a antagonist intervention; antagomiR-neg, antagomiR negative control; TEM, transmission electron microscope.

Disordered and broken myocardial fibers and irregular nuclear size were observed in rats from DM and antagomiR-neg groups when compared to the NC group; however, antagomiR-34a injection significantly alleviated this myocardial injury (Fig 8A). Transmission electron microscopic analysis revealed neatly arranged microfilaments, and a normal, closely arranged mitochondrial ultrastructure in rats from the NC group. Conversely, the rats from DM and antagomiR-neg groups exhibited broken myocardial sarcomeres, thinner microfilaments, swollen and deformed mitochondria, and ruptured and ablated mitochondrial membrane. However, the ultrastructure of myocardial tissues in rats from DE and antagomiR-34a groups improved significantly after 8 weeks of interventions (Fig 8A).

Quantitative analysis of interstitial and perivascular fibrosis areas using Masson’s trichrome staining revealed substantial collagen deposition in the myocardium of diabetic rats when compared with that of rats in the NC group, which was substantially alleviated by antagomiR-34a and swimming interventions (Fig 8A–8C). Similarly, immunohistochemical staining showed significant upregulation of Col I, Col III and TGF-β1 in the myocardium of T2DM rats when compared with that in the NC group, which was downregulated by antagomiR-34a and swimming interventions (Fig 8D–8G). These findings suggest that antagomiR-34a treatment attenuates myocardial fibrosis and restores the structural disorders of myocardial tissues in T2DM rats.

### 3.7. AntagomiR-34a activated the SIRT1/PGC-1α/FNDC5 signal pathway in the myocardium of T2DM rats

**Fig 9:**
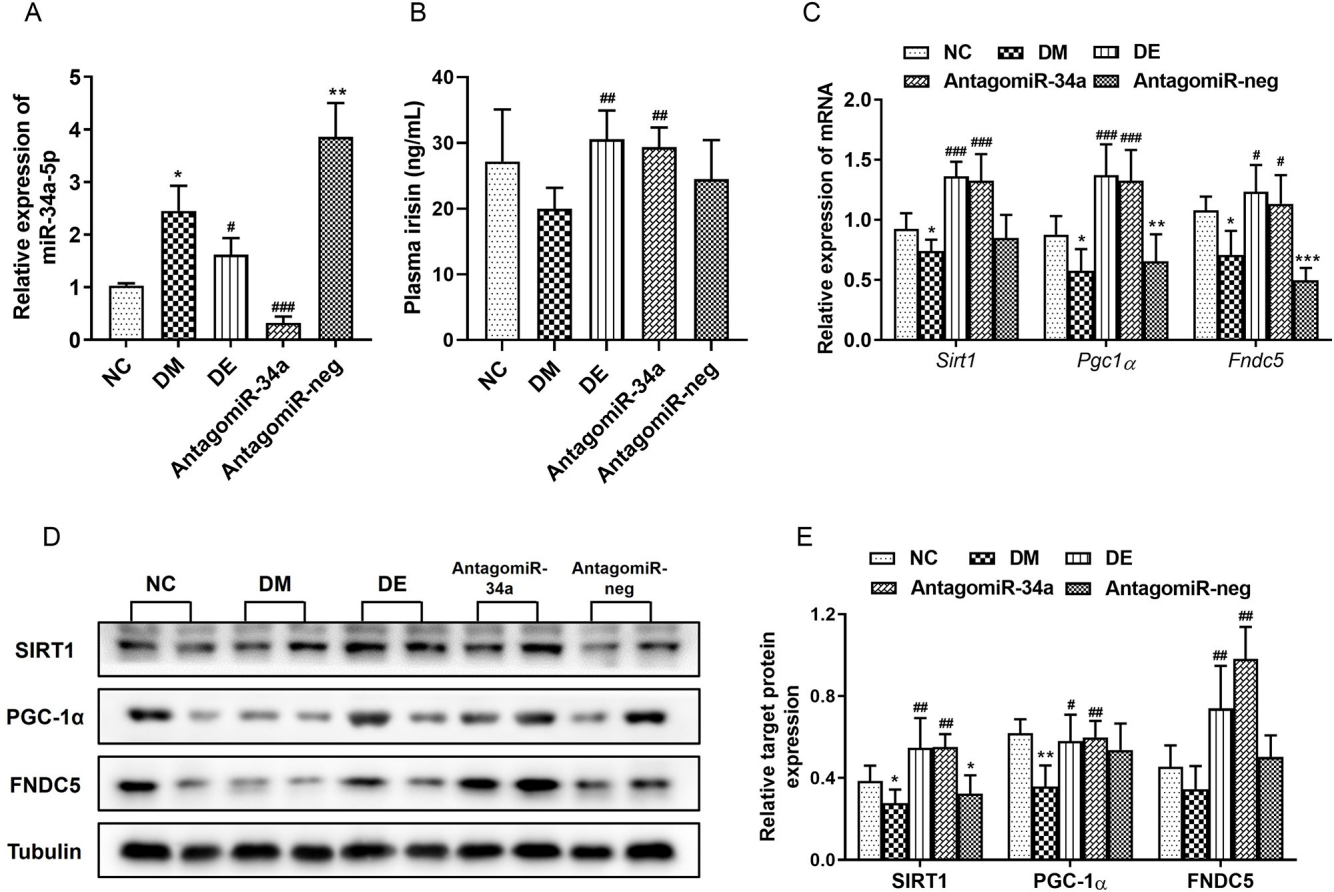
The plasma irisin levels and the expression of miR-34a, target genes and proteins. (**A**) Relative expression of miR-34a. **(B)** The levels of plasma irisin. **(C)** The mRNA expression of *Sirt1*, *Pgc1α*, and *Fndc5*. **(D)** Western blots for evaluating protein expression of SIRT1, PGC-1α, and FNDC5. **(E)** Corresponding statistical analysis for expression levels of SIRT1, PGC-1α, and FNDC5 proteins. All data are expressed as mean ± standard deviation (M ± SD). *P < 0.05, **P < 0.01, ***P < 0.001 versus NC group; ^#^*P* < 0.05, ^##^*P* < 0.01, ^###^*P* < 0.001 versus DM group. NC, normal control; DM, diabetic model; DE, diabetic-swimming exercise; antagomiR-34a, miR-34a antagonist intervention; antagomiR-neg, antagomiR negative control.

MiR-34a expression in the myocardium of the rats from DM and antagomiR-neg groups was significantly higher than that in the NC group. Furthermore, after treatment with the miR-34a antagonist for 8 weeks, the miR-34a expression level in T2DM rats was significantly reduced when compared with that in DM and antagomiR-neg groups (Fig 9A), confirming that the antagomiR-34a effectively inhibits miR-34a expression in heart tissues of T2DM rats. The expression of miR-34a in the myocardium of rats from the DE group also decreased significantly (Fig 9A). The plasma irisin levels of the rats from DE and antagomiR-34a groups were significantly increased when compared with those in the DM group (Fig 9B). Further experimental results showed that the expression levels of *Sirt1*, *Pgc1α*, and *Fndc5* mRNA in the myocardium of the rats from DM and antagomiR-neg groups significantly declined when compared with those in the NC group. However, after 8 weeks of antagomiR-34a treatment, the expression levels of these genes significantly upregulated in T2DM rats, showing a similar effect to the swimming intervention (Fig 9C). In addition, the expression of SIRT1, PGC-1α, and FNDC5 proteins in the myocardial tissue of the rats from the DM group was downregulated when compared with that in the NC group, which was also reversed upon antagomiR-34a treatment (Fig 9D and 9E). These results suggested that miR-34a suppression can activate the SIRT1/PGC-1α/FNDC5 signal pathway, thereby triggering irisin secretion in the myocardium of T2DM rats.

## 4. Discussion

**Fig 10:**
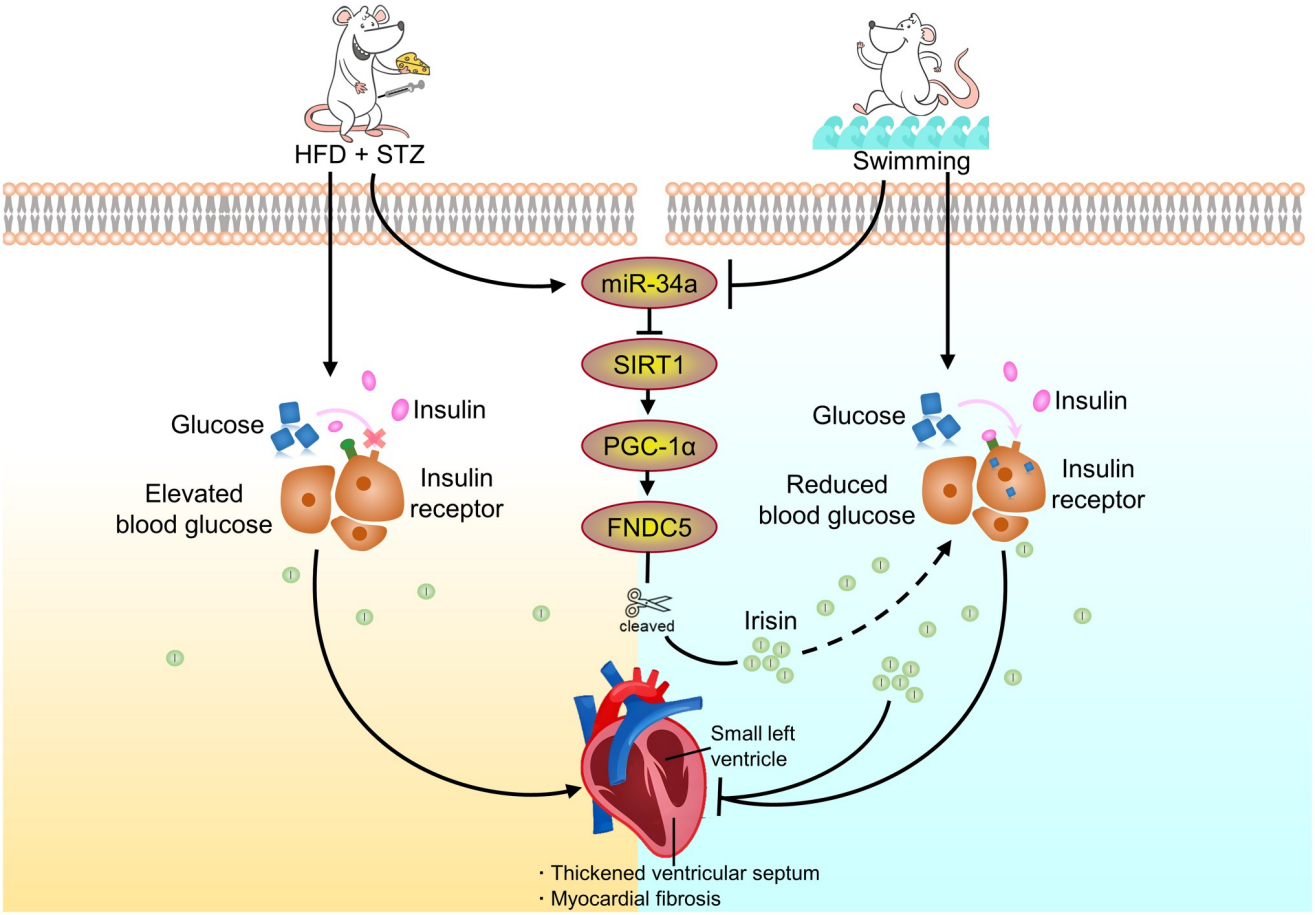
Swimming rescued the myocardial fibrosis of T2DM rats caused by HFD combined with STZ by inducing the release of irisin to alleviate insulin resistance and reduce blood glucose levels.

This study in male SD rats showed that swimming, as moderate intensity aerobic exercise for 8 weeks can reverse insulin resistance, reduce FBG levels, alleviate cardiac hypertrophy and myocardial fibrosis. This improvement is associated with irisin upregulation and miR-34a downregulation in the myocardium, thus enhancing diabetic cardiac function and mitigating myocardial fibrosis of T2DM rats, as summarized in Fig 10.

DCM is initially characterized by left ventricular hypertrophy and diastolic dysfunction that develops into systolic dysfunction as the disease progresses [35]. Human and animal experiments have indicated that myocardial fibrosis may be an important pathological mechanism in DCM [36, 37]. Myocardial fibrosis in DCM is caused by excessive collagen deposition in the ECM, thus leading to diffuse interstitial and perivascular fibrosis. Myocardial fibrosis plays a crucial role in the progression of several pathological changes associated with DCM, including LVH, diastolic, and systolic dysfunction [2]. Our study showed that T2DM caused ventricular hypertrophy, cardiac dysfunction and myocardial fibrosis. Therefore, anti-fibrotic treatment is essential for DCM.

Regular exercise training is an effective non-pharmacological intervention for the prevention and treatment of metabolic and diabetic cardiovascular complications [38, 39]. In aspects of regulating glucose metabolism, our study showed that 8 weeks of swimming intervention alleviated insulin resistance and glucose tolerance, and played a hypoglycemic role of T2DM rats. Further cardiac function test showed that swimming intervention can ameliorate cardiac dysfunction, including alleviating ventricular hypertrophy, reversing the decrease of EF and FS, and significantly reducing LVESV and LVEDV. Regarding myocardial fibrosis, our results suggest that fibrosis markers (Col I, Col III and TGF-β1) decreased in both exercise and r-irisin intervention groups. Previous animal studies have revealed that 8 weeks of moderate-intensity continuous exercise training was superior to high-intensity interval exercise training in rescuing the abnormal blood glucose and myocardial fibrosis in diabetic rats [40]. Additionally, an 8-week treadmill running program can reduce myocardial fibrosis in diabetic rats by increasing myocardial antioxidant capacity and inhibiting TGF-β1/Smad signal pathway [41]. Similarly, 12-week treadmill running training can reverse cardiac remodeling in T2DM mice by inhibiting the expression of P2X7 purinergic receptors in cardiomyocytes [42]. Combined with previous studies, in the present study, swimming was chosen as the exercise intervention, which is a moderate-intensity aerobic exercise. However, a recent study has demonstrated that high-intensity interval training (HIIT) for alleviating T2DM-related myocardial remodeling and fibrosis is superior to moderate-intensity continuous exercise training [43]. The inconsistency of these results may be highly correlated with animal strains, DCM models, exercise interventional cycle, and so on. Generally speaking, whether moderate-intensity aerobic exercise or HIIT, has a suppression effect on cardiac dysfunction in patients with diabetes.

Irisin, an exercise-induced myokine, is generated by cleavage of the extracellular ectodomain of FNDC5, and highly expressed in muscle tissues, especially myocardial and skeletal tissues [17, 44]. Patients with T2DM typically exhibit reduced circulating levels of irisin [45–48]. Our results showed that 8-week swimming intervention stimulated mRNA and protein expression of FNDC5 in myocardial tissues, which may be a major causative factor in ameliorating myocardial fibrosis in T2DM rats. A recent study has found that low-dose r-irisin (0.5 μg/g BW/d) could attenuate myocardial fibrosis and left ventricular dysfunction in type I diabetic mice by inhibiting high-glucose-induced endothelial-to-mesenchymal transition [27]. While high-dose r-irisin (1.5 μg/g BW/d) could disrupt normal expression of MMPs and induce the proliferation and metastasis of cardiac fibroblasts, thus resulting in excessive collagen deposition [27]. Combined with previous literature and experimental results, we chose r-irisin injection at the dose of 500 μg/kg BW in our study. These results show that the effect of r-irisin at this dose on myocardial fibrosis in T2DM rats is similar to exercise intervention. Meanwhile, our study proves that the optimal dose of exogenous irisin for treating diabetes-related cardiac dysfunction may be 500 μg/kg. Exercise-induced PGC-1α can stimulate the expression of FNDC5 [49]. Similarly, the recombinant irisin treatment also could induce the expression of FNDC5, which may due to the stimulation of energy expenditure for triggering AMPK or PGC-1α as a feedback mechanism. The production of irisin phosphorylates p38 MAPK and activates extracellular signal-regulated kinase (ERK), thereby leading to white fat browning, increased energy expenditure, and upregulation of uncoupling protein 1 (UCP1), thus alleviating HFD-induced obesity and insulin resistance *in vivo* [49]. Recent data indicate that treadmill running reduces myocardial fibrosis and inflammation, and upregulates irisin levels in the pristane-induced arthritis male rats, therefore, irisin is negatively correlated with myocardial inflammation and fibrosis [50]. The mechanism of exercise-induced irisin and r-irisin in reducing blood glucose and enhancing cardiac function of diabetes is not well understood, but irisin may be a promising treatment option for such conditions including persons with physical disability or patients under certain disease status losing physical capacity due to its mimetic exercise efficacy.

Molecular alterations caused by hyperglycemia is an important inducement of the onset and progression of DCM in diabetes. In this context, cardiac miRNAs play a crucial role in the transcriptional and post-transcriptional regulation of cardiovascular fibrosis, dysfunction and heart failure [51, 52]. A previous study has demonstrated that miR-34a is associated with myocardial fibrosis and aging in T2DM patients [53]. In the present study, high miR-34a expression was observed in the myocardium of T2DM rats, further confirming its involvement in the occurrence and development of DCM. One study has identified that miR-34a can modulate functional deterioration of MSCs under the condition of chronic hyperglycemia, and miR-34a inhibitor-treated MSC transplantation can improve cardiac function in diabetic rats [15]. The inhibition of miR-34a *in vivo* can reduce the severity of myocardial fibrosis in experimental mice, while miR-34a overexpression can increase the fibrogenic activity of TGF-β1 in cardiac fibroblasts. *In vitro* studies have confirmed that miR-34a plays a significant role in the progression of myocardial fibrosis by directly targeting Smad4 [13]. Contrary to other studies, a recent report illustrates that miR-34a ameliorates myocardial fibrosis by targeting Pin-1 signaling to suppress Col I production, viability, and migration in DCM [54]. These findings also suggest that glucose metabolism-related myocardial fibrosis may bring out differential targeting effects of miR-34a, while the mechanisms by whether exercise or exercise-induced irisin can execute the mitigation of miR-34a-mediated myocardial fibrosis are unknown. Our previous studies have revealed that swimming intervention can delay the aging process by inhibiting miR-34a, rescuing dysfunctional autophagy, and restoring abnormal mitochondrial dynamics in rats with D-galactose-induced oxidative stress and cognitive impairment [55]. Furthermore, swimming can prevent pancreatic apoptosis in T2DM rats subjected to an HFD combined with STZ through the miR-34a/SIRT1/p53 signal axis [56]. However, it has not been reported whether exercise-mediated miR-34a can affect the functional changes of diabetic heart. MiR-34a is the first miRNA found to regulate SIRT1, thus playing an important role in regulating cell senescence and angiogenesis through its effect on SIRT1 expression. SIRT1 is highly expressed in the normal heart, while significantly decreased in diabetic heart. In this study, we have found that exercise can inhibit the expression of miR-34a in the heart, and its downstream target protein SIRT1 is activated. SIRT1 can ameliorate the metabolism of diabetic individuals by regulating the activity of cytoplasmic protein PGC-1α. In response to exercise, PGC-1α leads to the production of the FNDC5 protein, which is cleaved to generate irisin, thus contributing to mitigating DCM. We have also observed that the gene and protein expression of FNDC5 in the heart of T2DM rats are increased significantly after injection of miR-34a inhibitor *in vivo*, which is consistent with the results from swimming intervention. Our findings have confirmed that swimming can promote glucose homeostasis by triggering the generation and secretion of FNDC5/irisin via miR-34a-dependent post-transcriptional mechanisms, thus suppressing myocardial fibrosis of T2DM rats.

Nevertheless, it is important to point out some limitations in the present study: First, considering that estrogen may mitigate insulin resistance-induced cardiomyopathy [57, 58], we used male rats only in the entire experiment. Second, due to individual difference, it is impossible to ensure that all T2DM rats enrolled in the experimental group have same symptoms of DCM at the same time point. Third, considering the risk of *in situ* cardiac injection and the consistency of swimming intervention, we have adopted tail vein injection, which may not comprehensively reflect the effect of antagomiR-34a on the heart. Finally, no miR-34a-specific knockout mice have been used, and the selectivity of pharmacological inhibitors is relative. Thus, future studies are essential to address these limitations and verify the results of the present study.

## 5. Conclusion

Swimming can trigger the generation and secretion of irisin to execute its functions in the myocardial tissue, thereby alleviating diabetic myocardial fibrosis through the miR-34a-mediated SIRT1/PGC-1α/FNDC5 signal pathway. These findings also suggest that exogenous r-irisin could be used as a substitute for exercise in treating patients with advanced diabetes who cannot engage in physical exercise due to physical disabilities or severe disease status.

## Supporting information

S1 File (ZIP)
