# Supplementary material for: Swimming alleviates myocardial fibrosis of type II diabetic rats through activating miR-34a-mediated SIRT1/PGC-1α/FNDC5 signal pathway
Source: PLoS One. 2024 Sep 9;19(9):e0310136. doi: 10.1371/journal.pone.0310136 (PMC11383238; doi:10.1371/journal.pone.0310136)
Supplement: S1 File — (ZIP) [file pone.0310136.s001.zip › Original data-GYJ-20240628/File 1. The original data of Figure.2 and Figure. 6/The data of FBG in different group rats.docx]

**Statistical analysis results of GraphPad Prism version 9.0 software**

**The original data of Figure.2 statistics table**

**The data of FBG in different group rats**

|  | 0 week | 2 week | 4 week | 6 week | 8 week | Repeated measurement F test | |
| --- | --- | --- | --- | --- | --- | --- | --- |
|  | M ± SD | M ± SD | M ± SD | M ± SD | M ± SD | *F*（DFn,DFd） | *P* |
| NC | 5.28 ± 0.732 | 6.28 ± 0.481 | 6.30 ± 0.458 | 6.46 ± 0.550 | 5.12 ± 0.909 |  | |
| DM | 17.24 ± 2.459 | 23.76 ± 1.904 | 20.00 ± 5.480 | 21.06 ± 1.892 | 25.2 ± 2.673 |  |  |
| DI | 18.28 ± 1.971 | 11.70 ± 1.878 | 18.82 ± 3.523 | 18.44 ± 3.573 | 18.48 ± 4.725 |  |  |
| DE | 17.48 ± 2.027 | 18.20 ± 3.914 | 15.42 ± 3.815 | 18.02 ± 2.299 | 15.06 ± 2.633 |  |  |
| Group main effect | | | | | | F (3, 16) = 150.1 | < 0.001 |
| Time main effect | | | | | | F (2.611, 41.77) = 1.017 | 0.3871 |
| Group x time interaction effect | | | | | | F (12, 64) = 4.327 | < 0.001 |

**The data of glucose tolerance in different group rats**

|  | 0 min | 30 min | 60 min | 90 min | 120 min | Repeated measurement F test | |
| --- | --- | --- | --- | --- | --- | --- | --- |
|  | M ± SD | M ± SD | M ± SD | M ± SD | M ± SD | *F*（DFn, DFd） | *P* |
| NC | 5.12 ± 0.909 | 7.62 ± 2.513 | 7.16 ± 1.744 | 6.60 ± 1.124 | 5.92 ± 1.035 |  | |
| DM | 23.32 ± 2.725 | 33.30 ± 0.000 | 32.36 ± 1.515 | 29.18 ± 2.847 | 26.66 ± 2.633 |  |  |
| DI | 23.02 ± 2.298 | 33.30 ± 0.000 | 30.68 ± 1.847 | 26.76 ± 2.035 | 23.16 ± 2.117 |  |  |
| DE | 21.58 ± 2.290 | 31.36 ± 4.337 | 33.04 ± 0.477 | 28.40 ± 4.216 | 25.06 ± 1.429 |  |  |
| Group main effect | | | | | | F (3,16) = 249.6 | < 0.001 |
| Time main effect | | | | | | F (2.353,37.64) = 76.05 | < 0.001 |
| Group x time interaction effect | | | | | | F (12,64) = 5.673 | < 0.001 |

**The original data of Figure.6 statistics table**

**The data of FBG in different group rats**

|  | 0 week | 2 week | 4 week | 6 week | 8 week | Repeated measurement F test | |
| --- | --- | --- | --- | --- | --- | --- | --- |
|  | M ± SD | M ± SD | M ± SD | M ± SD | M ± SD | *F*（DFn,DFd） | *P* |
| NC | 6.10 ± 0.223 | 5.17 ± 0.372 | 5.42 ± 0.335 | 5.04 ± 0.720 | 4.80 ± 0.635 |  | |
| DM | 21.90 ± 4.652 | 26.92 ± 3.798 | 27.06 ± 4.412 | 21.63 ± 1.927 | 24.68 ± 4.612 |  |  |
| DE | 22.03 ± 4.508 | 21.12 ± 3.593 | 18.49 ± 5.778 | 16.74 ± 4.476 | 14.22 ± 5.515 |  |  |
| Antagomir-34a | 22.21 ± 3.990 | 25.07 ± 4.210 | 24.50 ± 5.800 | 21.88 ± 3.349 | 15.28 ± 6.128 |  |  |
| Antagomir-neg | 22.65 ± 3.330 | 23.70 ± 2.694 | 24.18 ± 3.074 | 24.21 ± 2.519 | 21.90 ± 3.638 |  | |
| Group main effect | | | | | | F (4, 34) = 49.52 | < 0.001 |
| Time main effect | | | | | | F (3.504, 119.1) = 12.37 | < 0.001 |
| Group x time interaction effect | | | | | | F (16, 136) = 4.403 | < 0.001 |

**The data of glucose tolerance in different group rats**

|  | 0 min | 30 min | 60 min | 90 min | 120 min | Repeated measurement F test | |
| --- | --- | --- | --- | --- | --- | --- | --- |
|  | M ± SD | M ± SD | M ± SD | M ± SD | M ± SD | *F*（DFn,DFd） | *P* |
| NC | 4.80 ± 0.635 | 7.18 ± 0.601 | 7.64 ± 0.842 | 7.01 ± 0.313 | 6.21 ± 0.323 |  | |
| DM | 11.55 ± 4.882 | 24.92 ± 5.138 | 23.92 ± 4.274 | 23.47 ± 2.329 | 21.86 ± 4.110 |  |  |
| DE | 6.98 ± 2.293 | 17.56 ± 4.638 | 19.27 ± 3.800 | 17.32 ± 3.385 | 14.67 ± 4.053 |  |  |
| Antagomir-34a | 15.28 ± 6.128 | 25.20 ± 2.906 | 25.42 ± 4.972 | 21.90 ± 3.237 | 19.20 ± 3.284 |  |  |
| Antagomir-neg | 16.90 ± 7.696 | 26.10 ± 4.170 | 28.36 ± 3.633 | 25.38 ± 3.948 | 22.26 ± 4.985 |  | |
| Group main effect | | | | | | F (4, 35) =29.90 | < 0.001 |
| Time main effect | | | | | | F (2.606, 91.22) = 90.07 | < 0.001 |
| Group x time interaction effect | | | | | | F (16, 140) = 4.061 | < 0.001 |

**Statistical analysis results of SPSS version** **22.0 software**

**The original data of Figure.2**

The data of FBG in different group rats

|  | 0 week | 2 week | 4 week | 6 week | 8 week | Repeated measurement F test | |
| --- | --- | --- | --- | --- | --- | --- | --- |
|  | M ± SD | M ± SD | M ± SD | M ± SD | M ± SD | *F* | *P* |
| NC | 5.28 ± 0.732 | 6.28 ± 0.481 | 6.30 ± 0.458 | 6.46 ± 0.550 | 5.12 ± 0.909 |  | |
| DM | 17.24 ± 2.459 | 23.76 ± 1.904 | 20.00 ± 5.480 | 21.06 ± 1.892 | 25.2 ± 2.673 |  |  |
| DI | 18.28 ± 1.971 | 11.70 ± 1.878 | 18.82 ± 3.523 | 18.44 ± 3.573 | 18.48 ± 4.725 |  |  |
| DE | 17.48 ± 2.027 | 18.20 ± 3.914 | 15.42 ± 3.815 | 18.02 ± 2.299 | 15.06 ± 2.633 |  |  |
| Group main effect | | | | | | 150.15 | < 0.001 |
| Time main effect | | | | | | 1.017 | 0.406 |
| Group x time interaction effect | | | | | | 4.327 | < 0.001 |
|  | | | | | |  |  |

The data of HOMA-IR in different group rats

| group | HOMA-IR | F test | |
| --- | --- | --- | --- |
|  | (M ± SD) | *F* | *P* |
| NC | 5.22 ± 1.072 | 53.211 | < 0.001 |
| DM | 24.82 ± 2.237 *** |  | < 0.001 |
| DI | 23.01 ± 4.684 |  |  |
| DE | 16.86 ± 2.690 ^##^ |  | 0.002 |

^***^*P* < 0.001 versus NC group; ^##^*P* < 0.01 versus DM and antagomiR-neg groups. NC, normal control; DM, diabetic model; antagomiR-34a, miR-34a antagonist intervention; antagomiR-neg, antagomir negative control.

The data of glucose tolerance in different group rats

|  | 0 min | 30 min | 60 min | 90 min | 120 min | Repeated measurement F test | |
| --- | --- | --- | --- | --- | --- | --- | --- |
|  | M ± SD | M ± SD | M ± SD | M ± SD | M ± SD | *F* | *P* |
| NC | 5.12 ± 0.909 | 7.62 ± 2.513 | 7.16 ± 1.744 | 6.60 ± 1.124 | 5.92 ± 1.035 |  | |
| DM | 23.32 ± 2.725 | 33.30 ± 0.000 | 32.36 ± 1.515 | 29.18 ± 2.847 | 26.66 ± 2.633 |  |  |
| DI | 23.02 ± 2.298 | 33.30 ± 0.000 | 30.68 ± 1.847 | 26.76 ± 2.035 | 23.16 ± 2.117 |  |  |
| DE | 21.58 ± 2.290 | 31.36 ± 4.337 | 33.04 ± 0.477 | 28.40 ± 4.216 | 25.06 ± 1.429 |  |  |
| Group main effect | | | | | | 249.63 | < 0.001 |
| Time main effect | | | | | | 76.048 | < 0.001 |
| Group x time interaction effect | | | | | | 5.673 | < 0.001 |

The data of AUC in different group rats

| group | AUC | F test | |
| --- | --- | --- | --- |
|  | (M ± SD) | *F* | *P* |
| NC | 0.22 ± 0.517 | 313.528 | < 0.001 |
| DM | 1.00 ± 0.396 *** |  | < 0.001 |
| DI | 0.90 ± 0.049 ^##^ |  | 0.003 |
| DE | 0.92 ± 0.040 ^#^ |  | 0.021 |

^***^*P* < 0.001 versus NC group; ^#^*P* < 0.05, ^##^*P* < 0.01 versus DM and antagomiR-neg groups. NC, normal control; DM, diabetic model; antagomiR-34a, miR-34a antagonist intervention; antagomiR-neg, antagomir negative control.

**The original data of Figure.6**

The data of FBG in different group rats

|  | 0 week | 2 week | 4 week | 6 week | 8 week | Repeated measurement F test | |
| --- | --- | --- | --- | --- | --- | --- | --- |
|  | M ± SD | M ± SD | M ± SD | M ± SD | M ± SD | *F* | *P* |
| NC | 6.10 ± 0.223 | 5.17 ± 0.372 | 5.42 ± 0.335 | 5.04 ± 0.720 | 4.80 ± 0.635 |  | |
| DM | 21.90 ± 4.652 | 26.92 ± 3.798 | 27.06 ± 4.412 | 21.63 ± 1.927 | 24.68 ± 4.612 |  |  |
| DE | 22.03 ± 4.508 | 21.12 ± 3.593 | 18.49 ± 5.778 | 16.74 ± 4.476 | 14.22 ± 5.515 |  |  |
| Antagomir-34a | 22.21 ± 3.990 | 25.07 ± 4.210 | 24.50 ± 5.800 | 21.88 ± 3.349 | 15.28 ± 6.128 |  |  |
| Antagomir-neg | 22.65 ± 3.330 | 23.70 ± 2.694 | 24.18 ± 3.074 | 24.21 ± 2.519 | 21.90 ± 3.638 |  | |
| Group main effect | | | | | | 150.15 | < 0.001 |
| Time main effect | | | | | | 49.516 | < 0.001 |
| Group x time interaction effect | | | | | | 4.403 | < 0.001 |

The data of HOMA-IR in different group rats

| group | HOMA-IR | F test | |
| --- | --- | --- | --- |
|  | (M ± SD) | *F* | *P* |
| NC | 7.13 ± 0.261 | 15.532 | < 0.001 |
| DM | 9.50 ± 0.944 *** |  | < 0.001 |
| DE | 8.37 ± 0.460 |  |  |
| Antagomir-34a | 7.49 ± 0.909**^#^** |  | 0.037 |
| Antagomir-neg | 8.72 ± 0.431*** |  | < 0.001 |

^***^*P* < 0.001 versus NC group; ^#^*P* < 0.05 versus DM and antagomiR-neg groups. NC, normal control; DM, diabetic model; antagomiR-34a, miR-34a antagonist intervention; antagomiR-neg, antagomir negative control.

The data of glucose tolerance in different group rats

|  | 0 min | 30 min | 60 min | 90 min | 120 min | Repeated measurement F test | |
| --- | --- | --- | --- | --- | --- | --- | --- |
|  | M ± SD | M ± SD | M ± SD | M ± SD | M ± SD | *F* | *P* |
| NC | 4.80 ± 0.635 | 7.18 ± 0.601 | 7.64 ± 0.842 | 7.01 ± 0.313 | 6.21 ± 0.323 |  | |
| DM | 11.55 ± 4.882 | 24.92 ± 5.138 | 23.92 ± 4.274 | 23.47 ± 2.329 | 21.86 ± 4.110 |  |  |
| DE | 6.98 ± 2.293 | 17.56 ± 4.638 | 19.27 ± 3.800 | 17.32 ± 3.385 | 14.67 ± 4.053 |  |  |
| Antagomir-34a | 15.28 ± 6.128 | 25.20 ± 2.906 | 25.42 ± 4.972 | 21.90 ± 3.237 | 19.20 ± 3.284 |  |  |
| Antagomir-neg | 16.90 ± 7.696 | 26.10 ± 4.170 | 28.36 ± 3.633 | 25.38 ± 3.948 | 22.26 ± 4.985 |  | |
| Group main effect | | | | | | 35.184 | < 0.001 |
| Time main effect | | | | | | 52.701 | < 0.001 |
| Group x time interaction effect | | | | | | 2.652 | 0.001 |

The data of AUC in different group rats

| group | AUC | F test | |
| --- | --- | --- | --- |
|  | (M ± SD) | *F* | *P* |
| NC | 13.63 ± 0.954 | 55.844 | < 0.001 |
| DM | 48.55 ± 7.272 *** |  | < 0.001 |
| DE | 35.72 ± 3.739^##^ |  | 0.005 |
| Antagomir-34a | 42.64 ± 6.321^#^ |  | 0.026 |
| Antagomir-neg | 46.74 ± 3.535 *** |  | < 0.001 |

^***^*P* < 0.001 versus NC group; ^#^*P* < 0.05 versus DM and antagomiR-neg groups. NC, normal control; DM, diabetic model; antagomiR-34a, miR-34a antagonist intervention; antagomiR-neg, antagomir negative control.
