# Supplementary figures and images for: Swimming alleviates myocardial fibrosis of type II diabetic rats through activating miR-34a-mediated SIRT1/PGC-1α/FNDC5 signal pathway
Source: PLoS One. 2024 Sep 9;19(9):e0310136. doi: 10.1371/journal.pone.0310136 (PMC11383238; doi:10.1371/journal.pone.0310136)

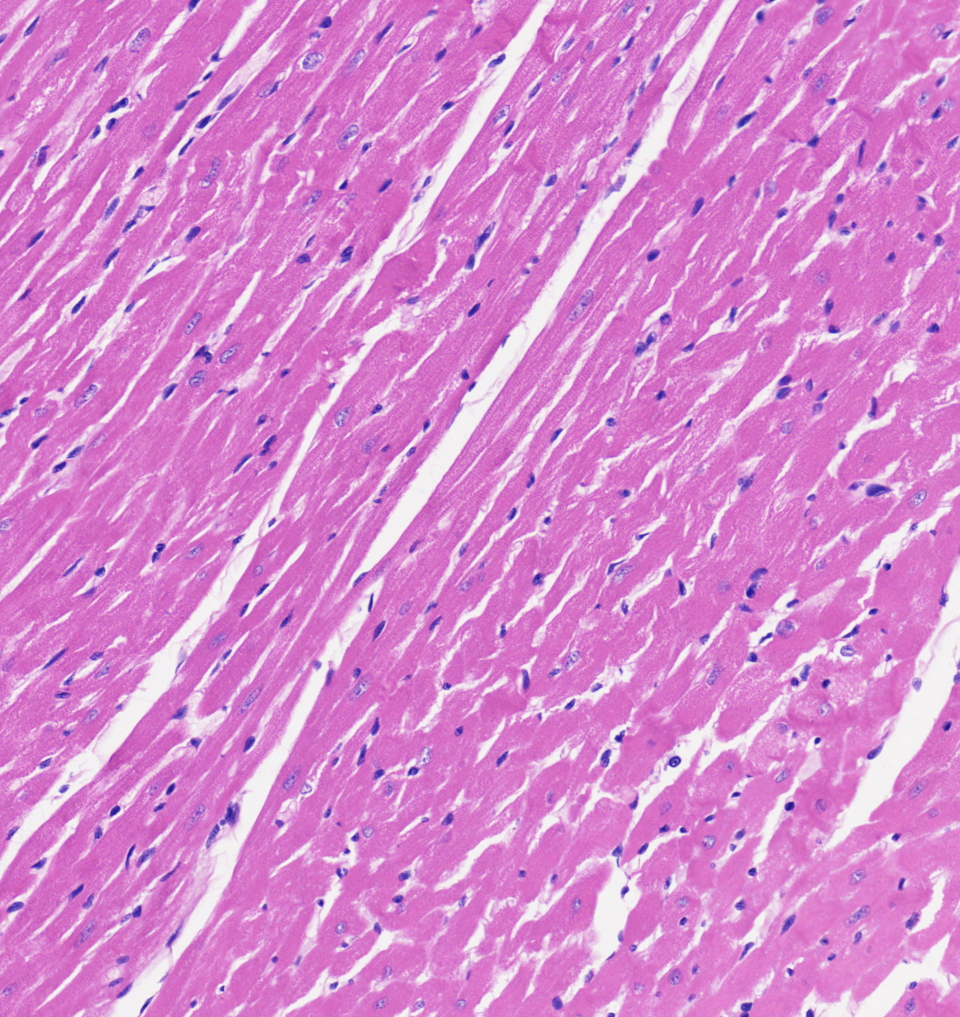

Supplement: S1 File — (ZIP) [file pone.0310136.s001.zip › Original data-GYJ-20240628/File 2. The original images of HE staining/Figure_4/DE 7 -A.svs_20.0x.jpg]

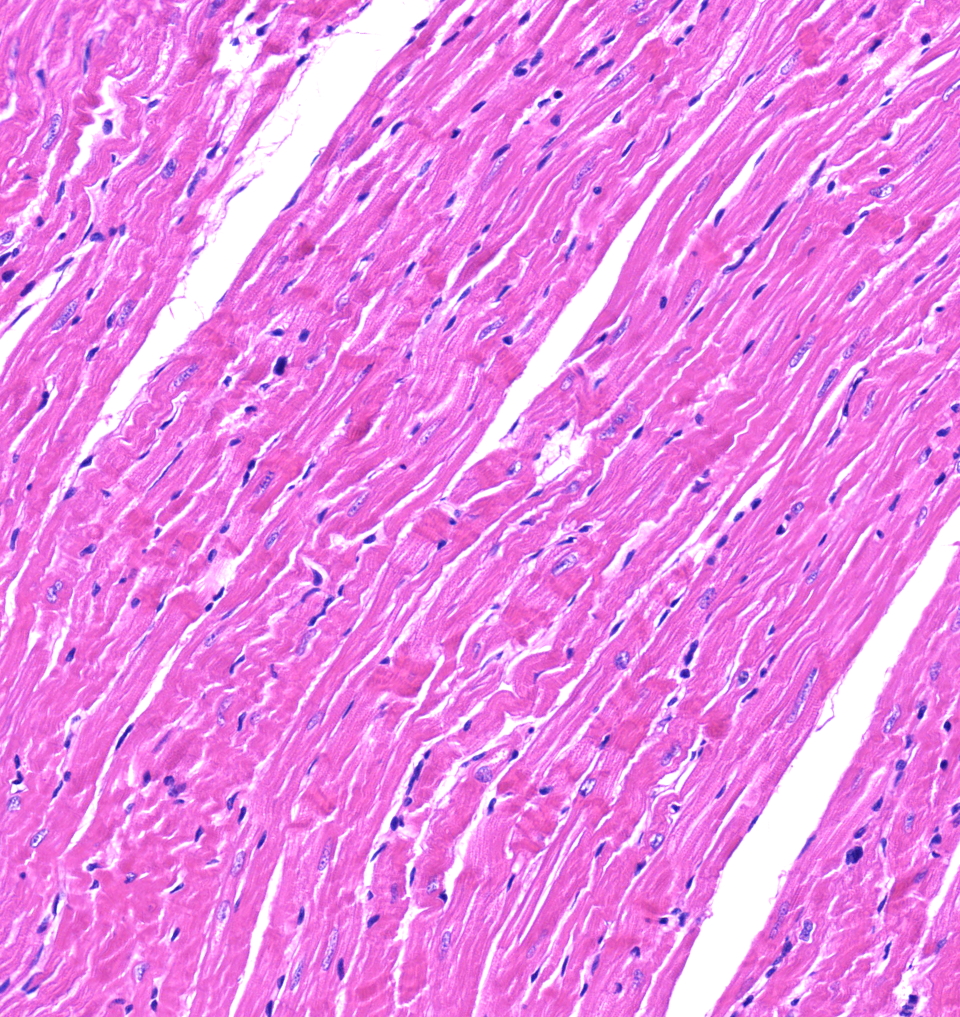

Supplement: S1 File — (ZIP) [file pone.0310136.s001.zip › Original data-GYJ-20240628/File 2. The original images of HE staining/Figure_4/DI-C.svs_20.0x.jpg]

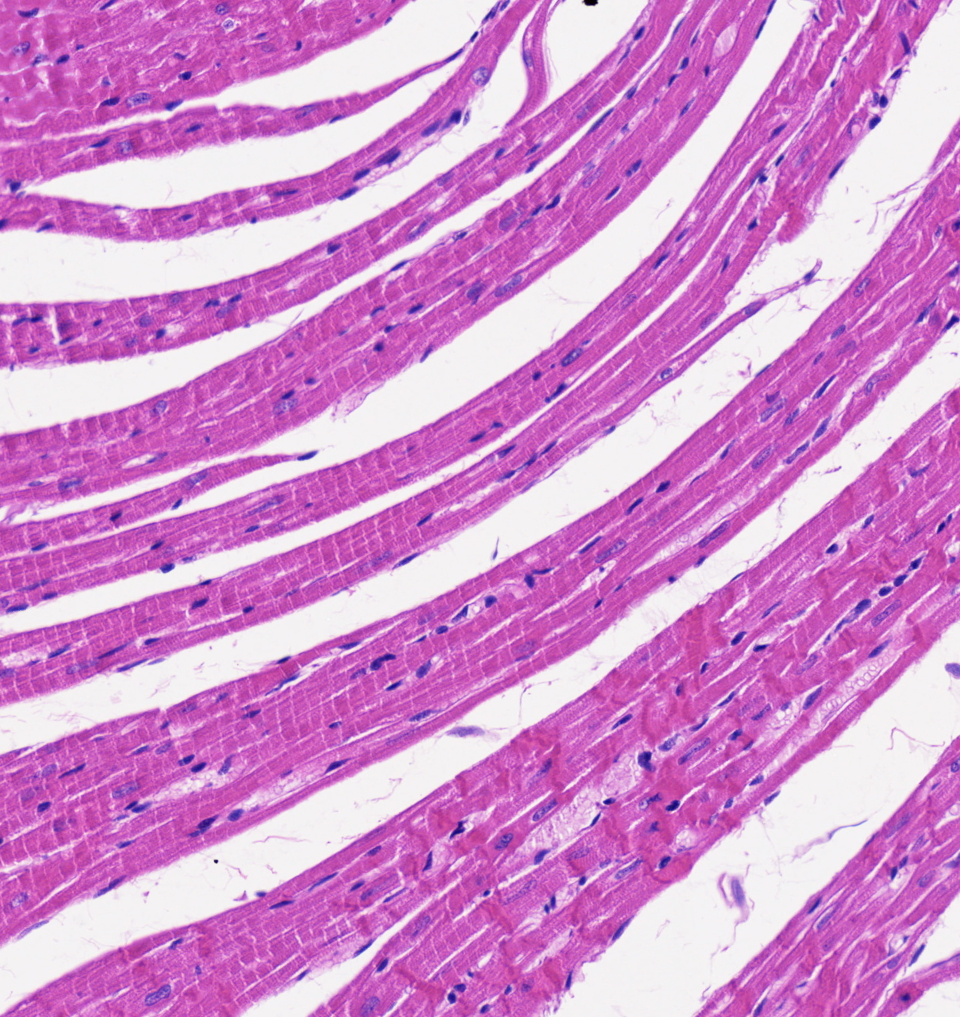

Supplement: S1 File — (ZIP) [file pone.0310136.s001.zip › Original data-GYJ-20240628/File 2. The original images of HE staining/Figure_4/DM -D.svs_20.0x.jpg]

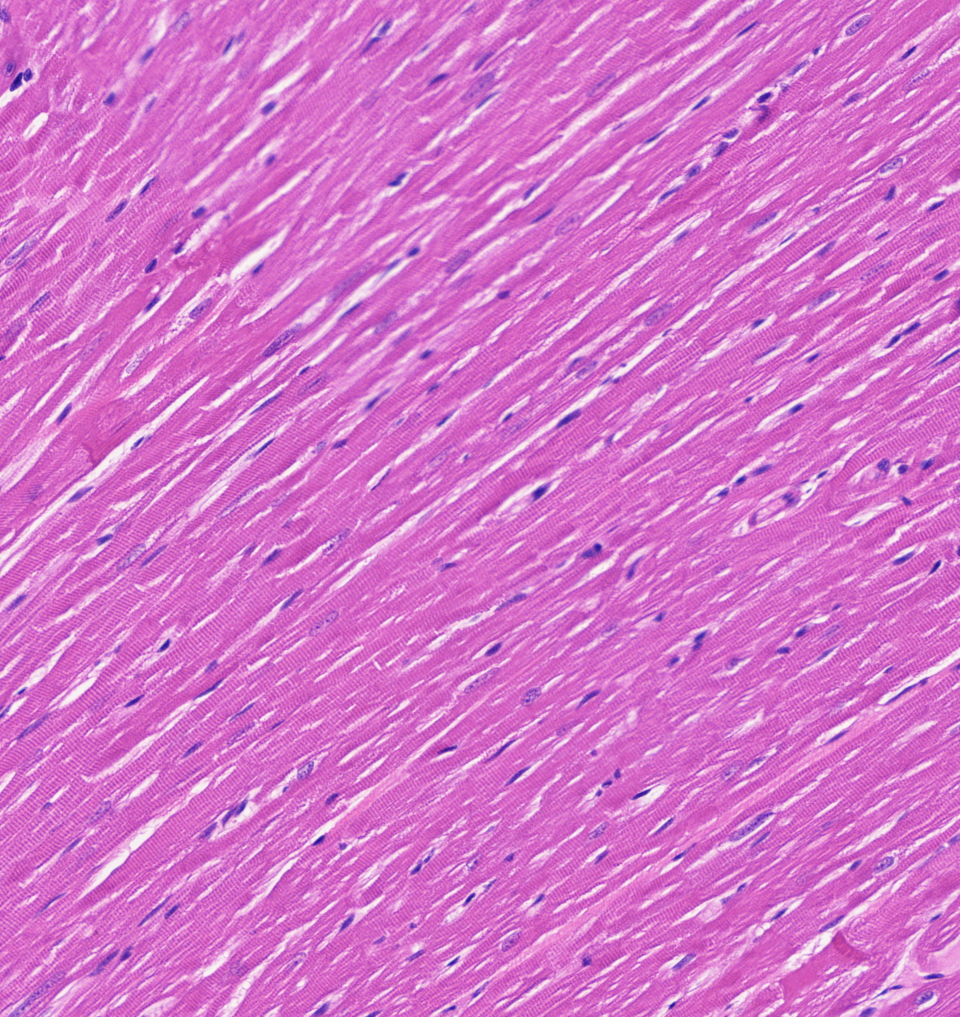

Supplement: S1 File — (ZIP) [file pone.0310136.s001.zip › Original data-GYJ-20240628/File 2. The original images of HE staining/Figure_4/NC 6.17-D.svs_20.0x.jpg]

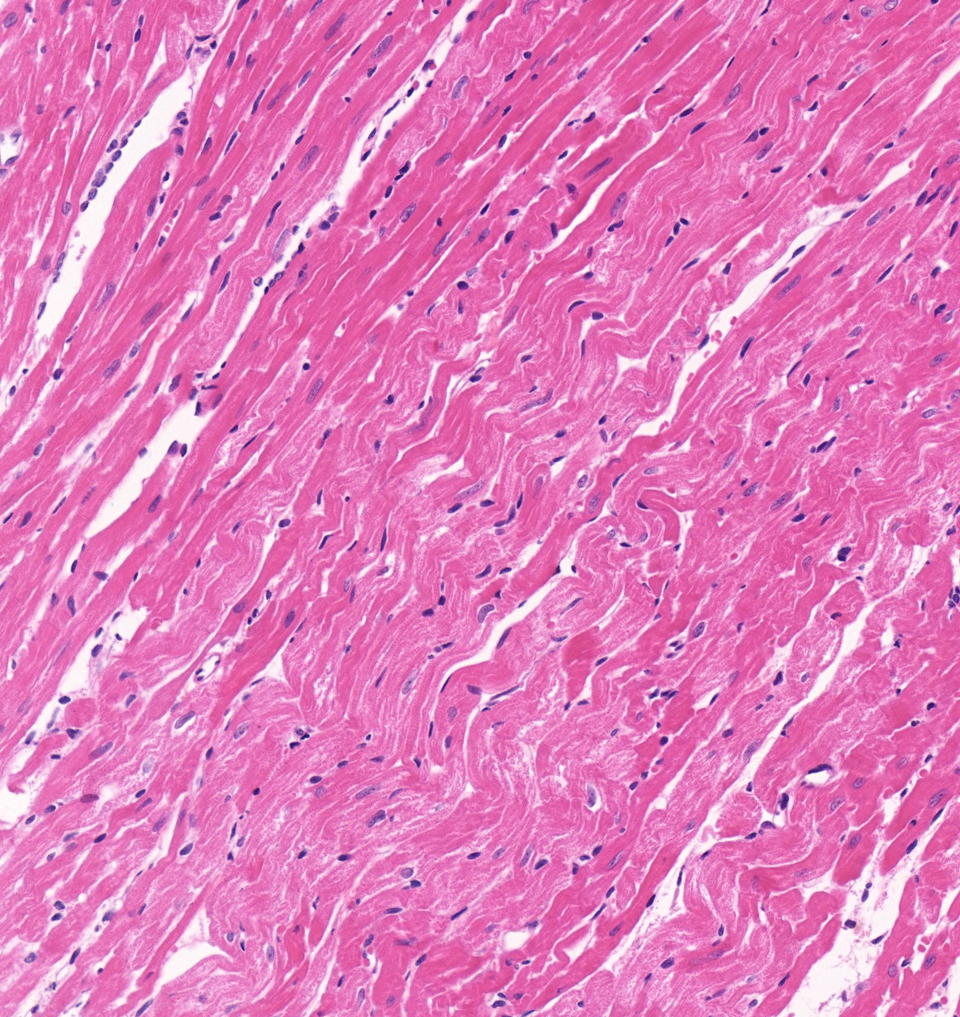

Supplement: S1 File — (ZIP) [file pone.0310136.s001.zip › Original data-GYJ-20240628/File 2. The original images of HE staining/Figure_8/Antagomir-34a -HE-G22-C_20.0x.jpg]

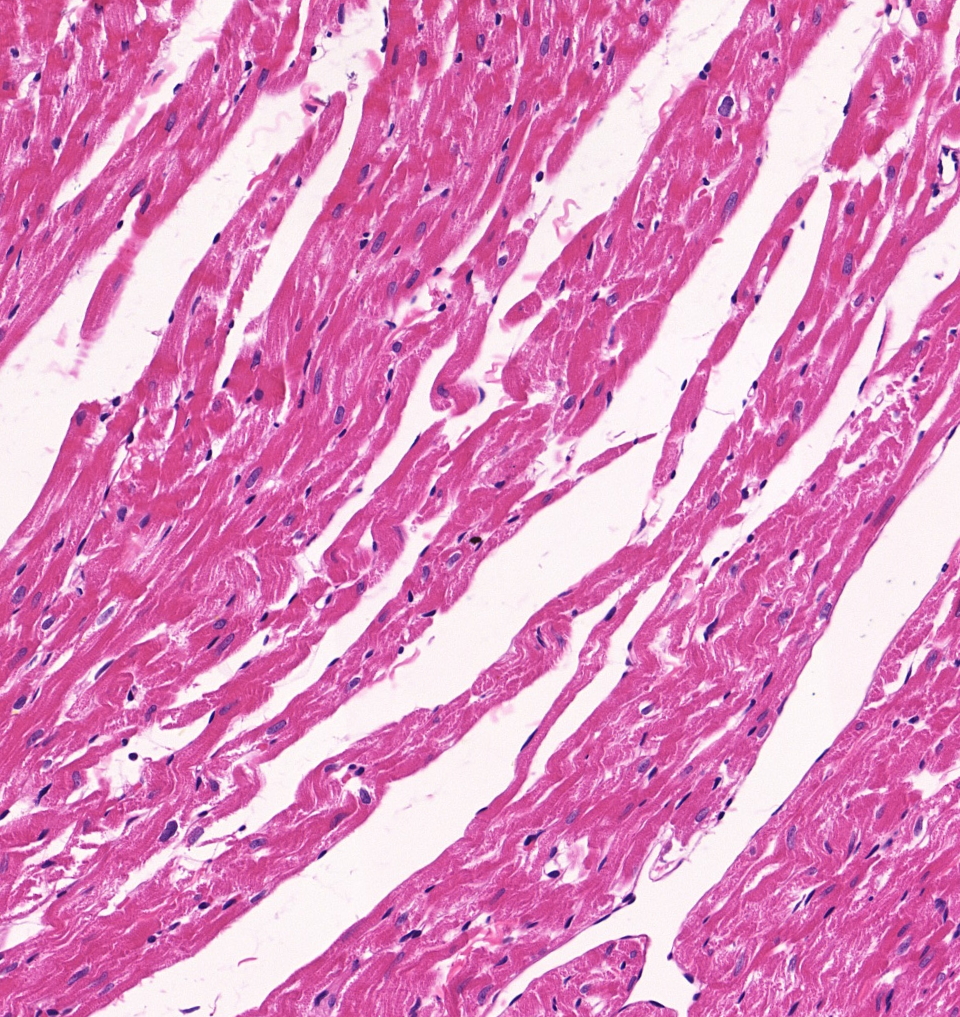

Supplement: S1 File — (ZIP) [file pone.0310136.s001.zip › Original data-GYJ-20240628/File 2. The original images of HE staining/Figure_8/Antagomir-neg -HE-H41-B_20.0x.jpg]

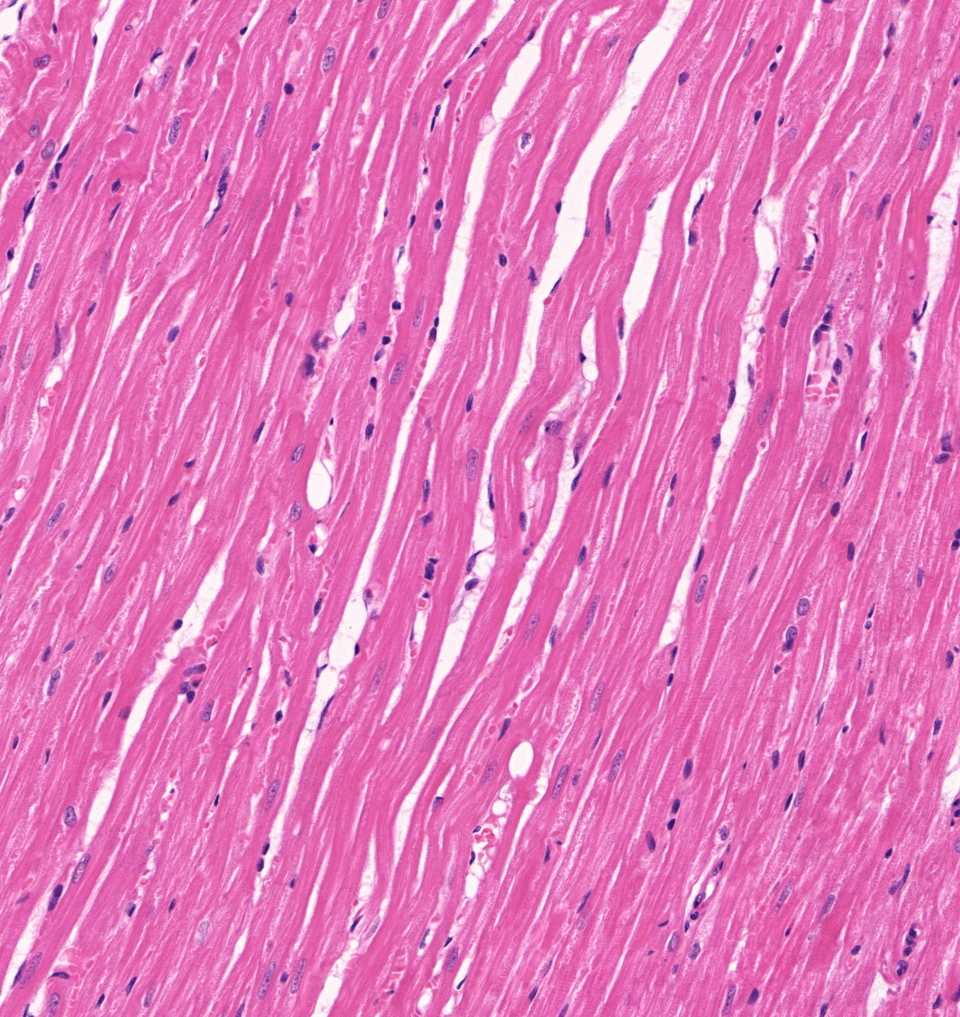

Supplement: S1 File — (ZIP) [file pone.0310136.s001.zip › Original data-GYJ-20240628/File 2. The original images of HE staining/Figure_8/DE-HE-E21-C_20.0x.jpg]

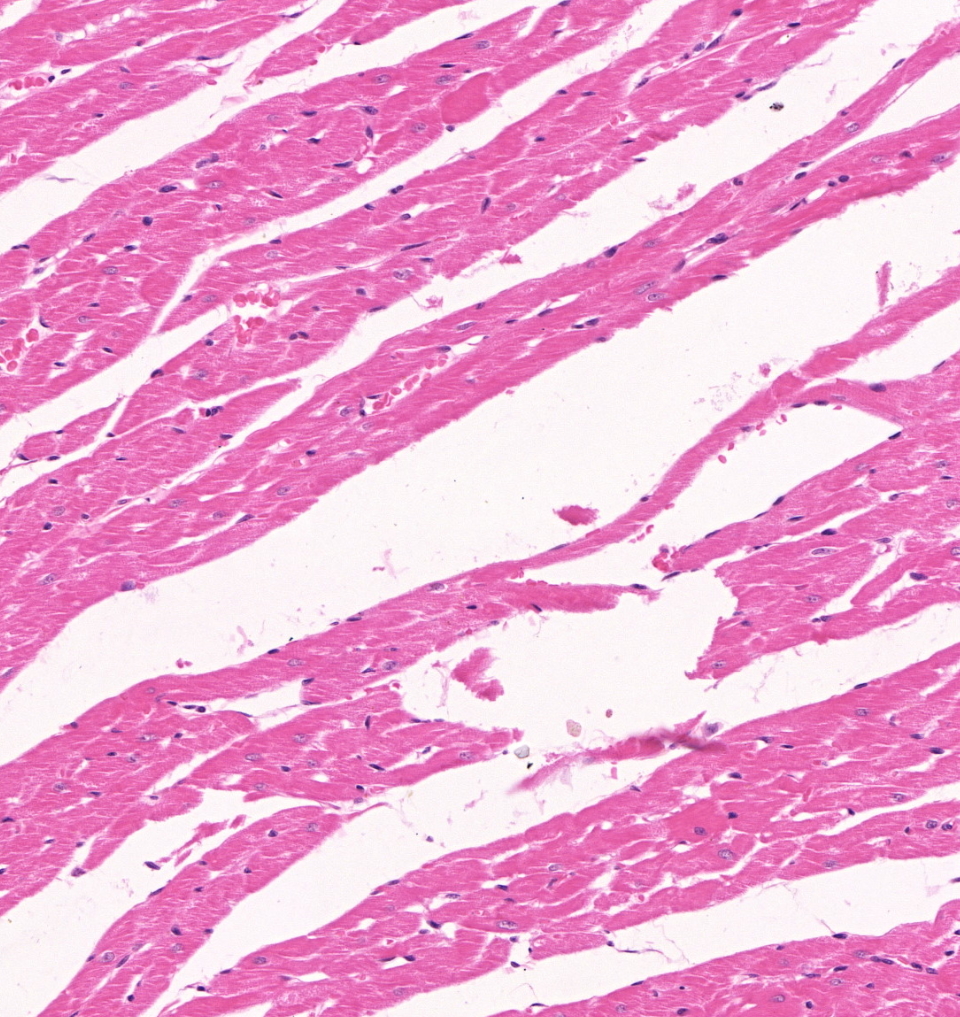

Supplement: S1 File — (ZIP) [file pone.0310136.s001.zip › Original data-GYJ-20240628/File 2. The original images of HE staining/Figure_8/DM-HE-D46-A_20.0x.jpg]

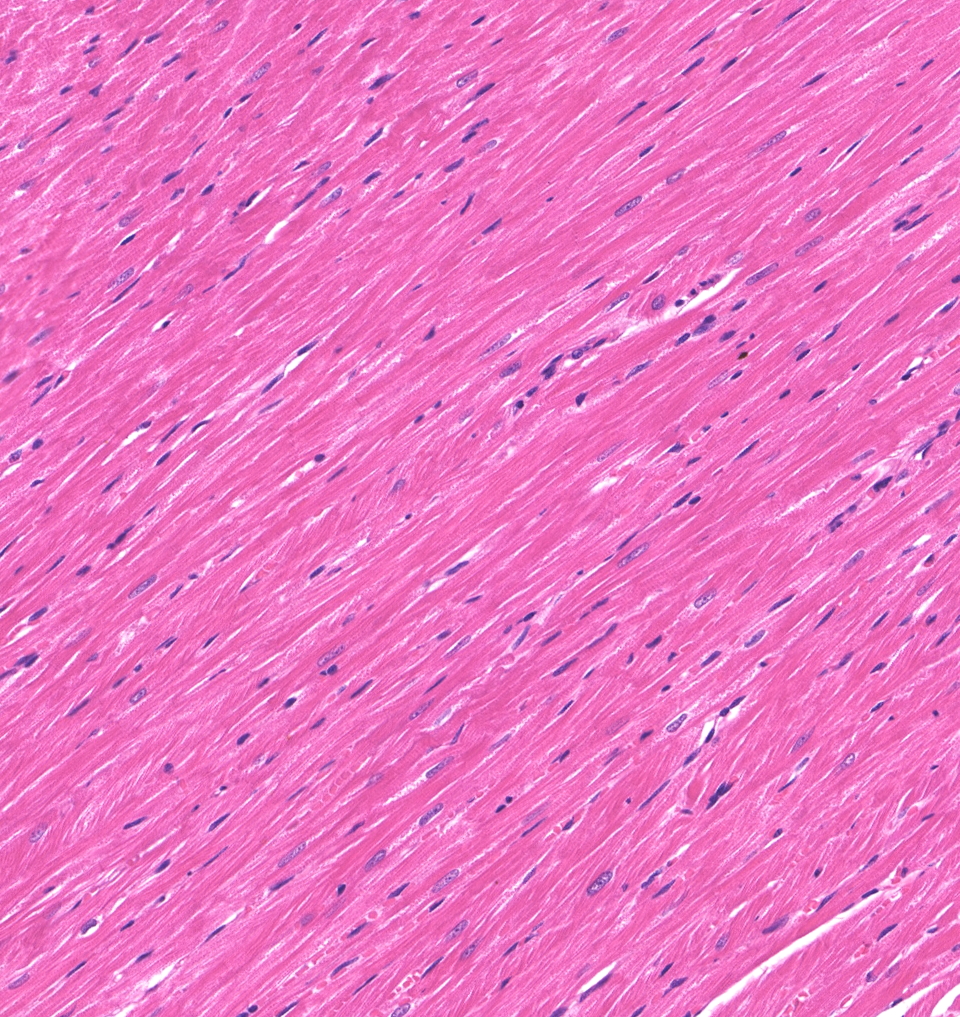

Supplement: S1 File — (ZIP) [file pone.0310136.s001.zip › Original data-GYJ-20240628/File 2. The original images of HE staining/Figure_8/NC-A3-A_20.0x.jpg]

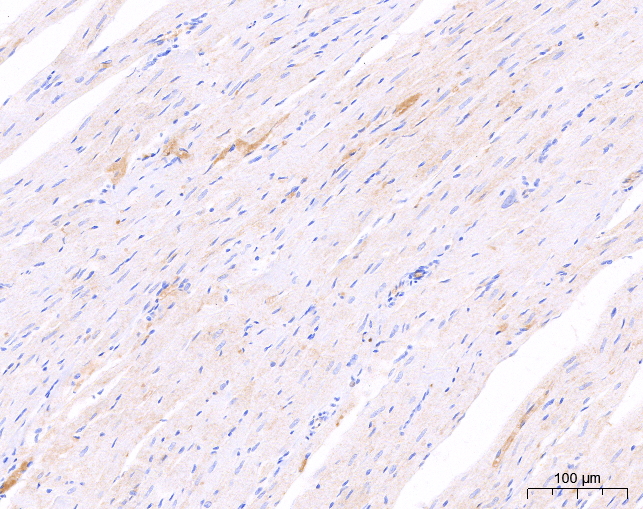

Supplement: S1 File — (ZIP) [file pone.0310136.s001.zip › Original data-GYJ-20240628/File 3. The original images of immumohistochemical staining/Figure_4/DE1 Collagen 1-B.svs_10.0x.jpg]

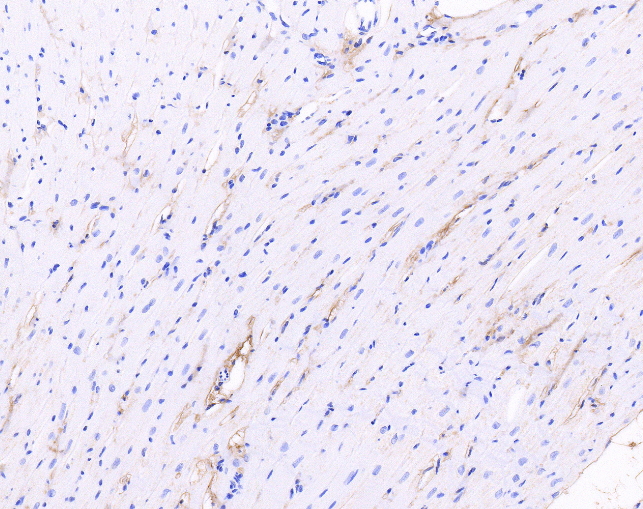

Supplement: S1 File — (ZIP) [file pone.0310136.s001.zip › Original data-GYJ-20240628/File 3. The original images of immumohistochemical staining/Figure_4/DE1 Collagen 3-A.svs_10.0x.jpg]

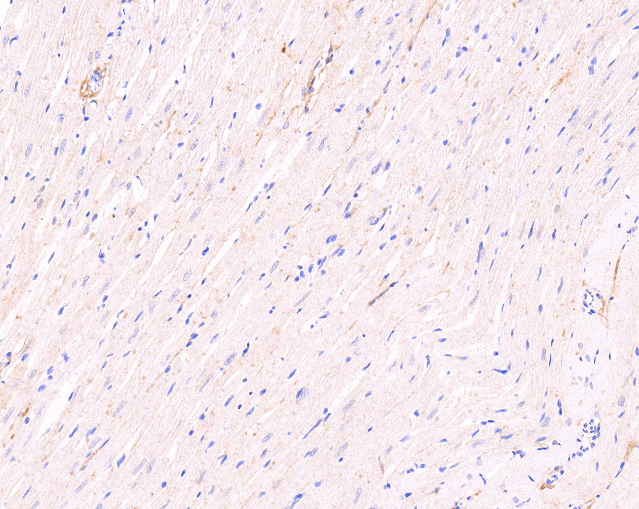

Supplement: S1 File — (ZIP) [file pone.0310136.s001.zip › Original data-GYJ-20240628/File 3. The original images of immumohistochemical staining/Figure_4/DE1 TGF-β1-A.svs_10.0x.jpg]

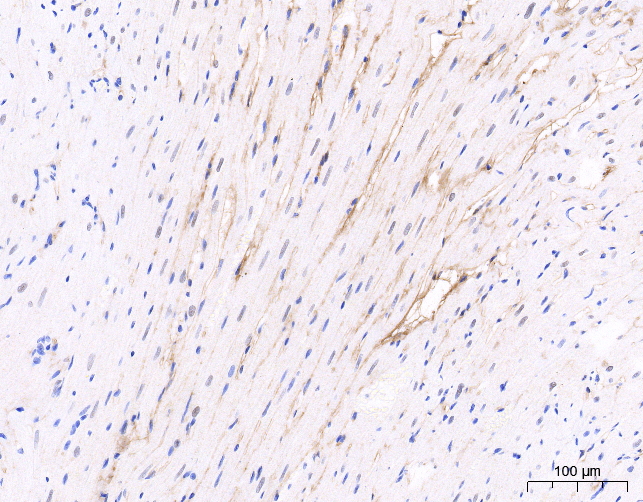

Supplement: S1 File — (ZIP) [file pone.0310136.s001.zip › Original data-GYJ-20240628/File 3. The original images of immumohistochemical staining/Figure_4/DI Collagen 3-B.svs_10.0x.jpg]

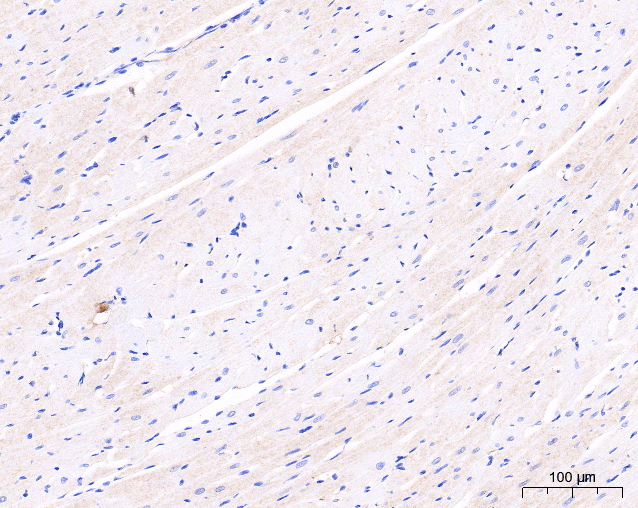

Supplement: S1 File — (ZIP) [file pone.0310136.s001.zip › Original data-GYJ-20240628/File 3. The original images of immumohistochemical staining/Figure_4/DI Collagen-1-A.svs_10.0x.jpg]

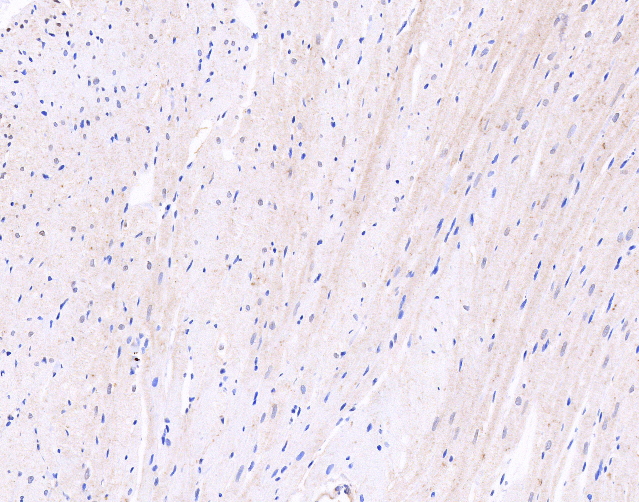

Supplement: S1 File — (ZIP) [file pone.0310136.s001.zip › Original data-GYJ-20240628/File 3. The original images of immumohistochemical staining/Figure_4/DI TGF-β1-A.svs_10.0x.jpg]

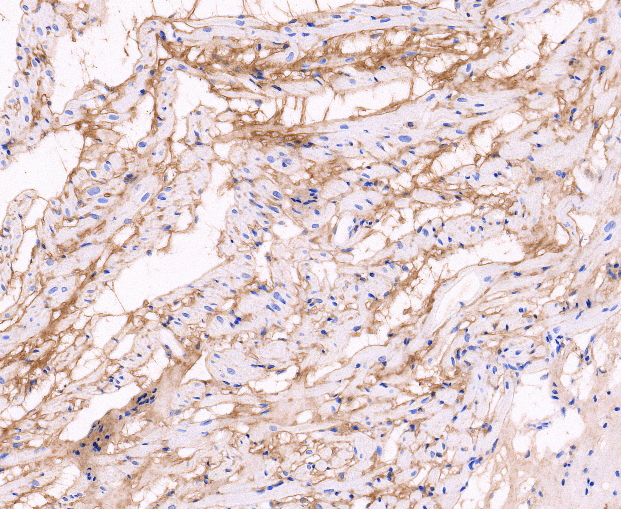

Supplement: S1 File — (ZIP) [file pone.0310136.s001.zip › Original data-GYJ-20240628/File 3. The original images of immumohistochemical staining/Figure_4/DM7 Collagen 3-A.svs_10.0x.jpg]

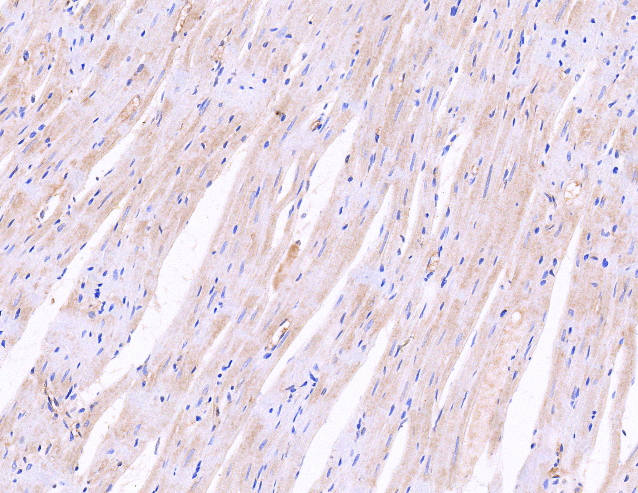

Supplement: S1 File — (ZIP) [file pone.0310136.s001.zip › Original data-GYJ-20240628/File 3. The original images of immumohistochemical staining/Figure_4/DM7 Collagen1-2.svs_10.0x.jpg]

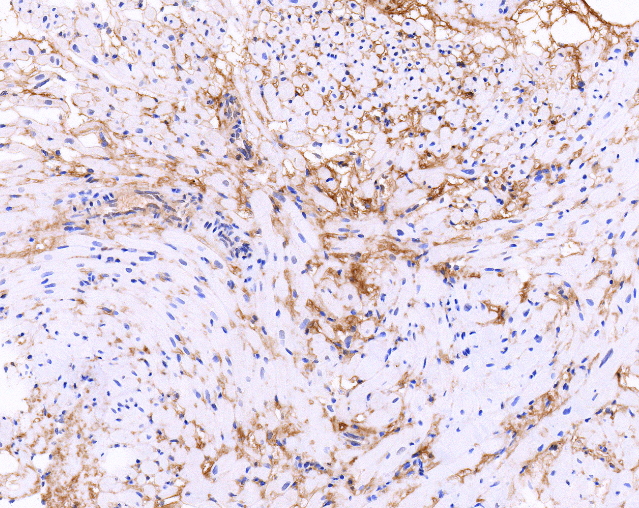

Supplement: S1 File — (ZIP) [file pone.0310136.s001.zip › Original data-GYJ-20240628/File 3. The original images of immumohistochemical staining/Figure_4/DM7 TGF-β1-G.svs_10.0x.jpg]

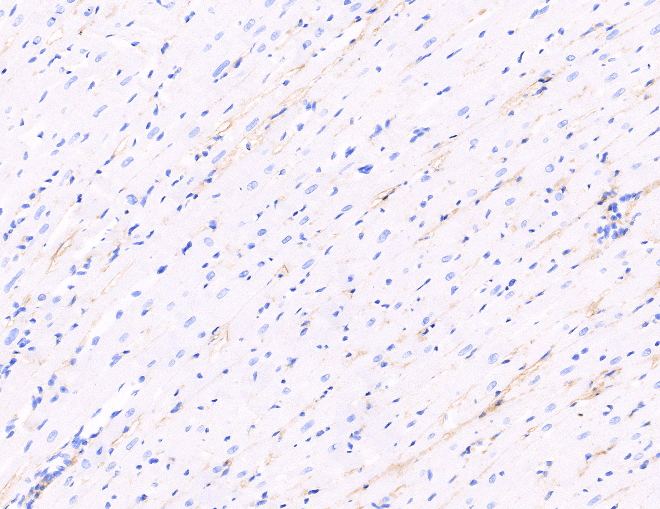

Supplement: S1 File — (ZIP) [file pone.0310136.s001.zip › Original data-GYJ-20240628/File 3. The original images of immumohistochemical staining/Figure_4/NC4 Collagen 3-B.svs_10.0x.jpg]

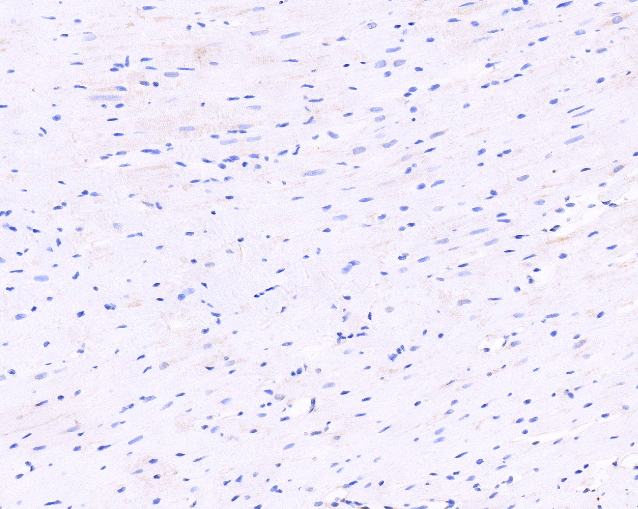

Supplement: S1 File — (ZIP) [file pone.0310136.s001.zip › Original data-GYJ-20240628/File 3. The original images of immumohistochemical staining/Figure_4/NC4 Collagen1-B.svs_10.0x.jpg]

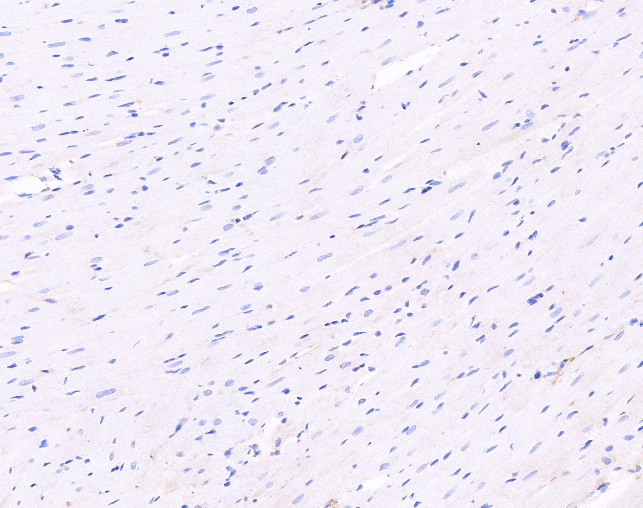

Supplement: S1 File — (ZIP) [file pone.0310136.s001.zip › Original data-GYJ-20240628/File 3. The original images of immumohistochemical staining/Figure_4/NC4 TGF-β1-B.svs_10.0x.jpg]

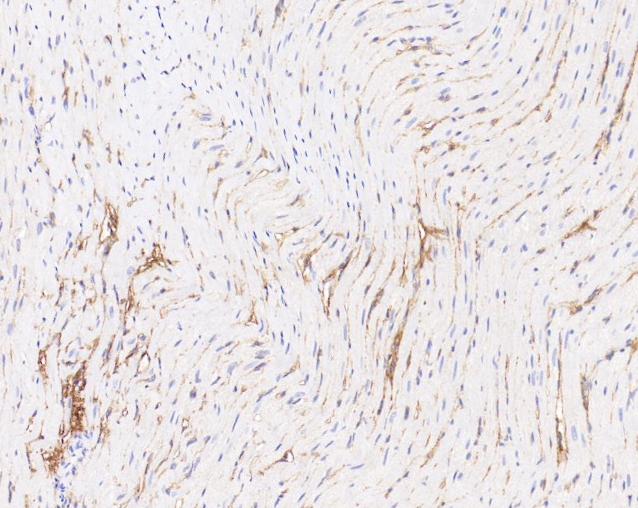

Supplement: S1 File — (ZIP) [file pone.0310136.s001.zip › Original data-GYJ-20240628/File 3. The original images of immumohistochemical staining/Figure_8/Antagomir-34a Collagen 3_10.0x.jpg]

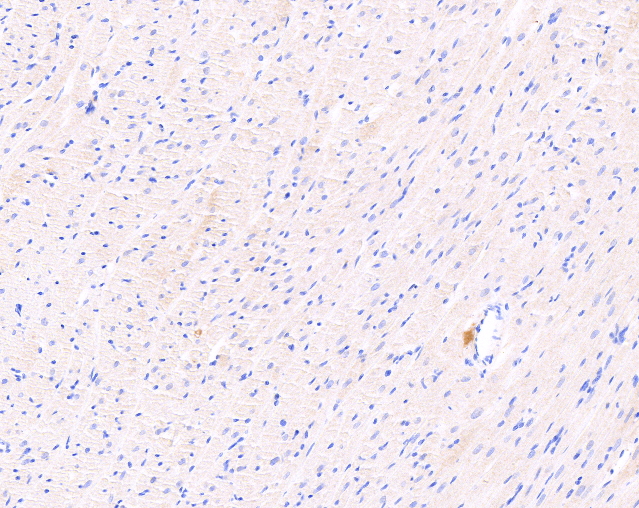

Supplement: S1 File — (ZIP) [file pone.0310136.s001.zip › Original data-GYJ-20240628/File 3. The original images of immumohistochemical staining/Figure_8/Antagomir-34a Collagen1-B.svs_10.0x.jpg]

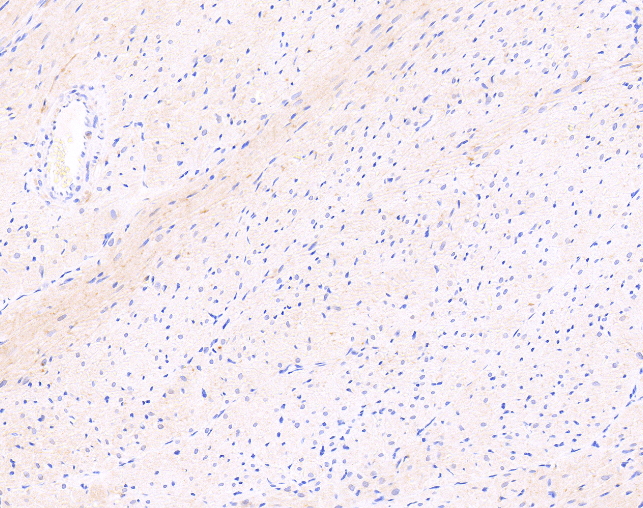

Supplement: S1 File — (ZIP) [file pone.0310136.s001.zip › Original data-GYJ-20240628/File 3. The original images of immumohistochemical staining/Figure_8/Antagomir-34a TGF-β1-B.svs_10.0x.jpg]

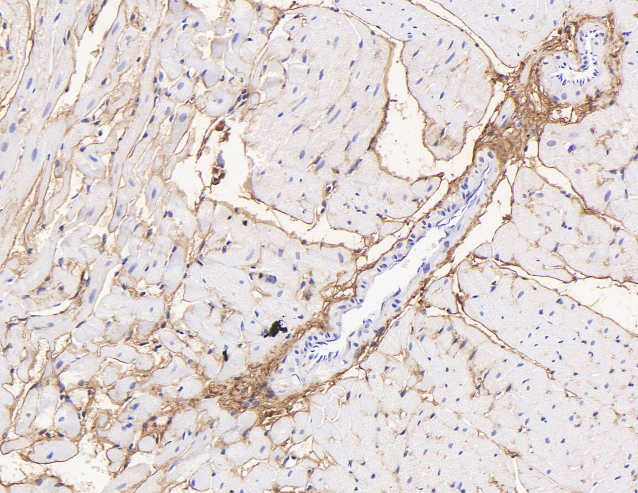

Supplement: S1 File — (ZIP) [file pone.0310136.s001.zip › Original data-GYJ-20240628/File 3. The original images of immumohistochemical staining/Figure_8/Antagomir-neg Collagen 3-A_10.0x.jpg]

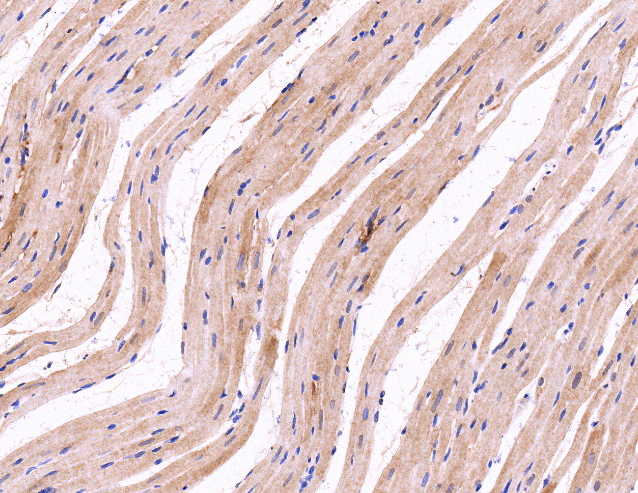

Supplement: S1 File — (ZIP) [file pone.0310136.s001.zip › Original data-GYJ-20240628/File 3. The original images of immumohistochemical staining/Figure_8/Antagomir-neg Collagen1.svs_10.0x.jpg]

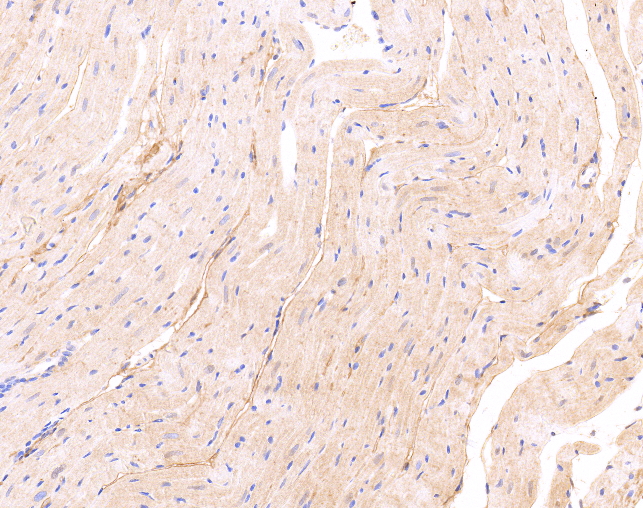

Supplement: S1 File — (ZIP) [file pone.0310136.s001.zip › Original data-GYJ-20240628/File 3. The original images of immumohistochemical staining/Figure_8/Antagomir-neg TGF-β1-C.svs_10.0x.jpg]

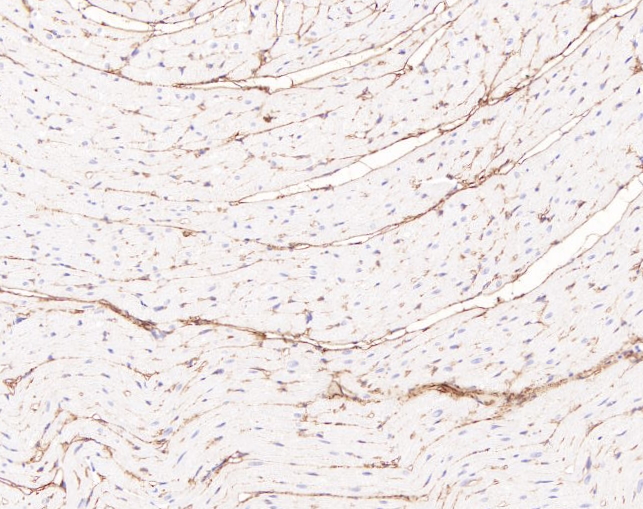

Supplement: S1 File — (ZIP) [file pone.0310136.s001.zip › Original data-GYJ-20240628/File 3. The original images of immumohistochemical staining/Figure_8/DE-Collagen 3-1_10.0x.jpg]

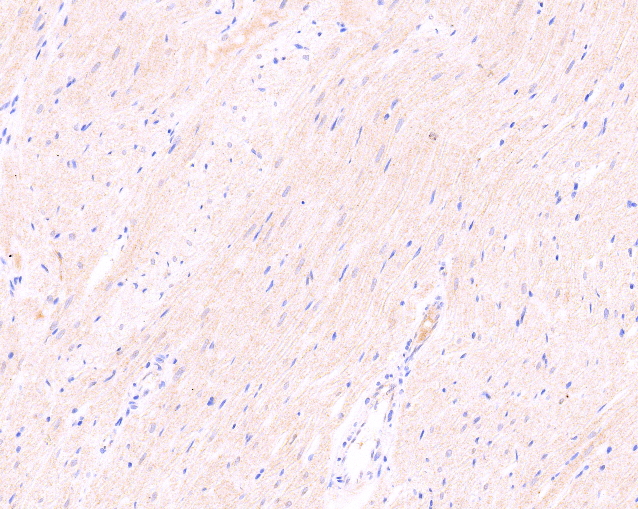

Supplement: S1 File — (ZIP) [file pone.0310136.s001.zip › Original data-GYJ-20240628/File 3. The original images of immumohistochemical staining/Figure_8/DE-Collagen1-A.svs_10.0x.jpg]

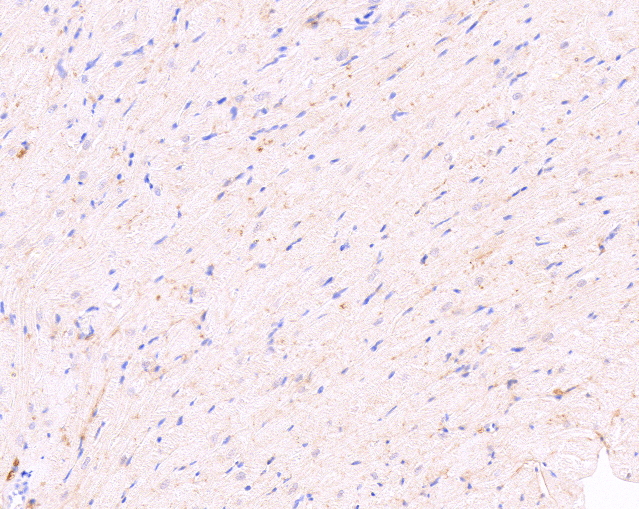

Supplement: S1 File — (ZIP) [file pone.0310136.s001.zip › Original data-GYJ-20240628/File 3. The original images of immumohistochemical staining/Figure_8/DE-TGF-β1-C.svs_10.0x.jpg]

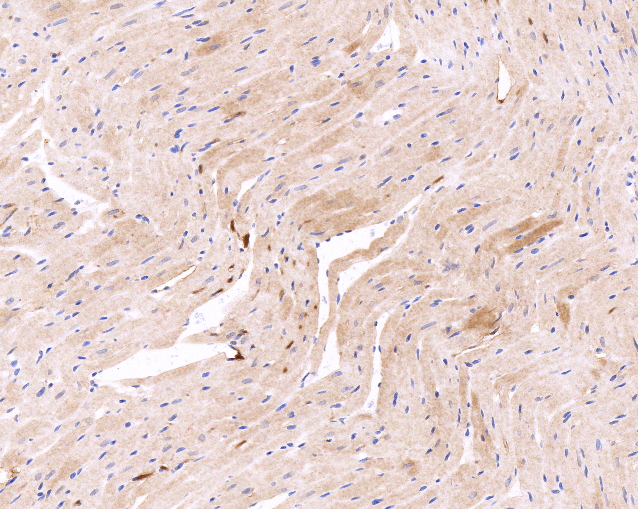

Supplement: S1 File — (ZIP) [file pone.0310136.s001.zip › Original data-GYJ-20240628/File 3. The original images of immumohistochemical staining/Figure_8/DM- Collagen1-A.svs_10.0x.jpg]

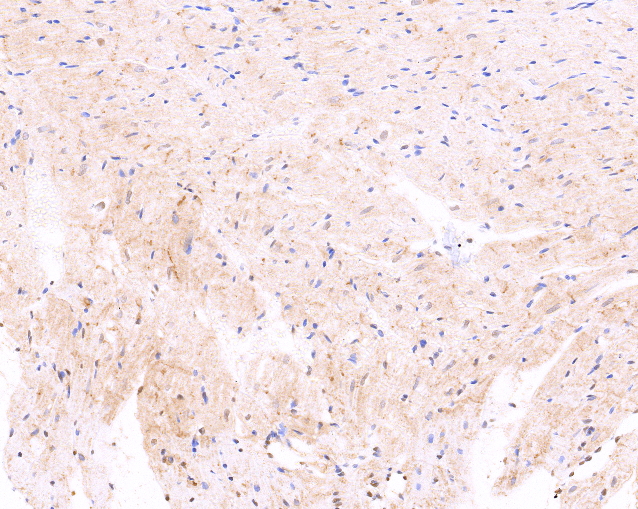

Supplement: S1 File — (ZIP) [file pone.0310136.s001.zip › Original data-GYJ-20240628/File 3. The original images of immumohistochemical staining/Figure_8/DM-TGF-β1-B.svs_10.0x.jpg]

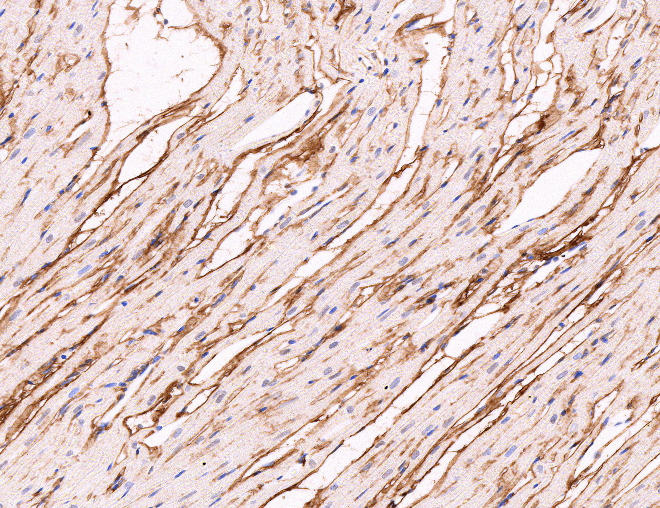

Supplement: S1 File — (ZIP) [file pone.0310136.s001.zip › Original data-GYJ-20240628/File 3. The original images of immumohistochemical staining/Figure_8/DM-collagen3-D.svs_10.0x.jpg]

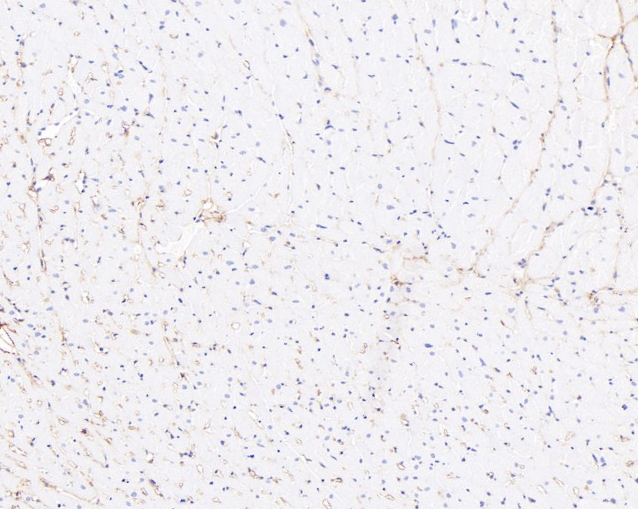

Supplement: S1 File — (ZIP) [file pone.0310136.s001.zip › Original data-GYJ-20240628/File 3. The original images of immumohistochemical staining/Figure_8/NC Collagen 3-3_10.0x.jpg]

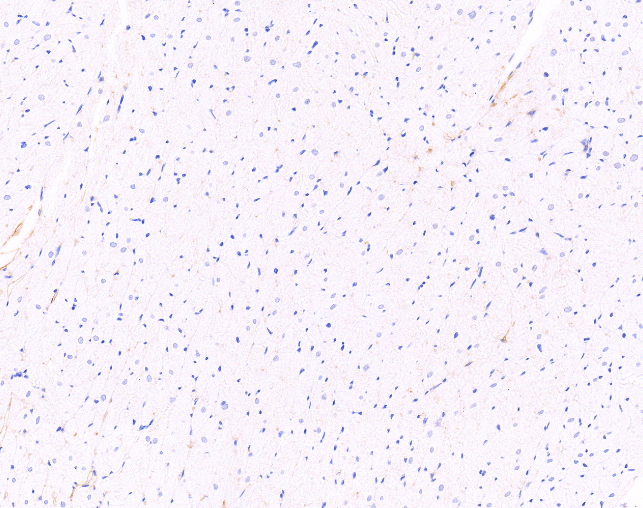

Supplement: S1 File — (ZIP) [file pone.0310136.s001.zip › Original data-GYJ-20240628/File 3. The original images of immumohistochemical staining/Figure_8/NC-TGF-β1-A.svs_10.0x.jpg]

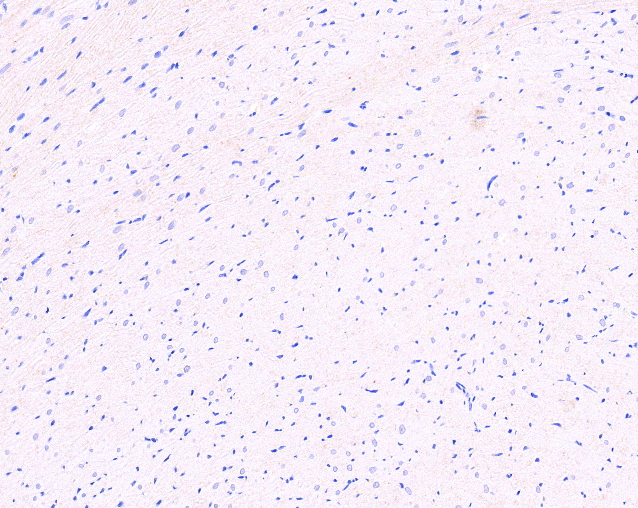

Supplement: S1 File — (ZIP) [file pone.0310136.s001.zip › Original data-GYJ-20240628/File 3. The original images of immumohistochemical staining/Figure_8/NC-collagen1-B.svs_10.0x.jpg]

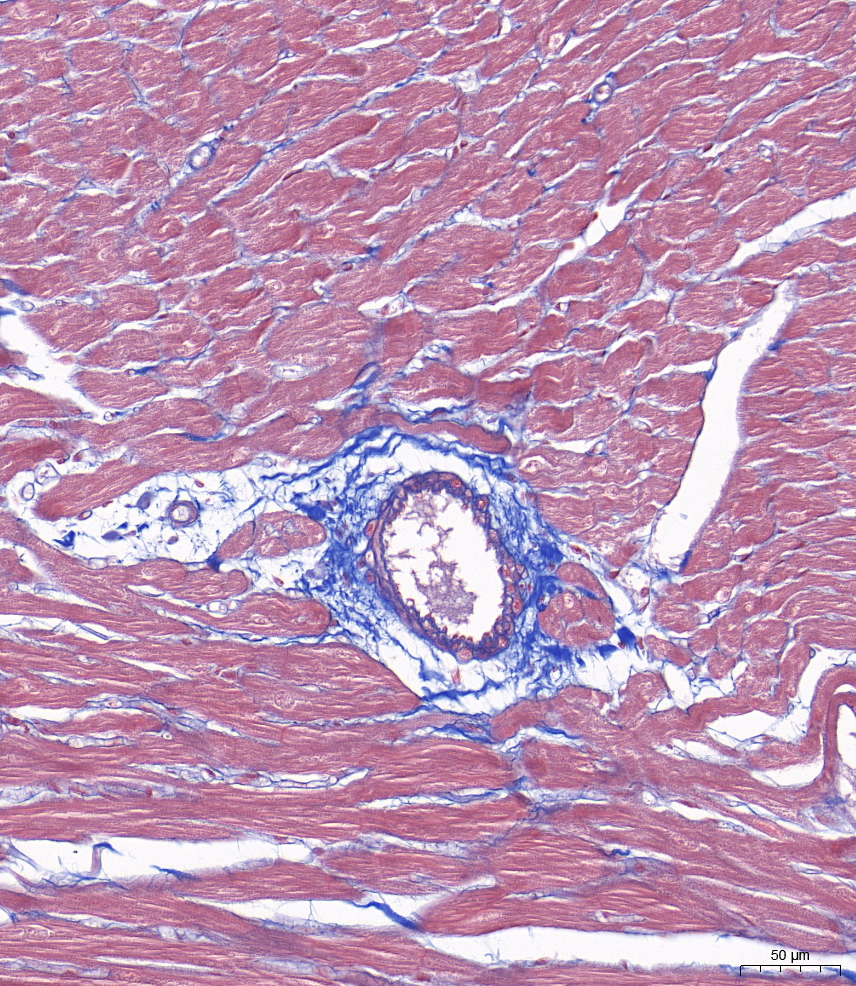

Supplement: S1 File — (ZIP) [file pone.0310136.s001.zip › Original data-GYJ-20240628/File 4. The original images of Masson staining/Figure_4/DE-Perivascular fibrosis_20.0x 2.jpg]

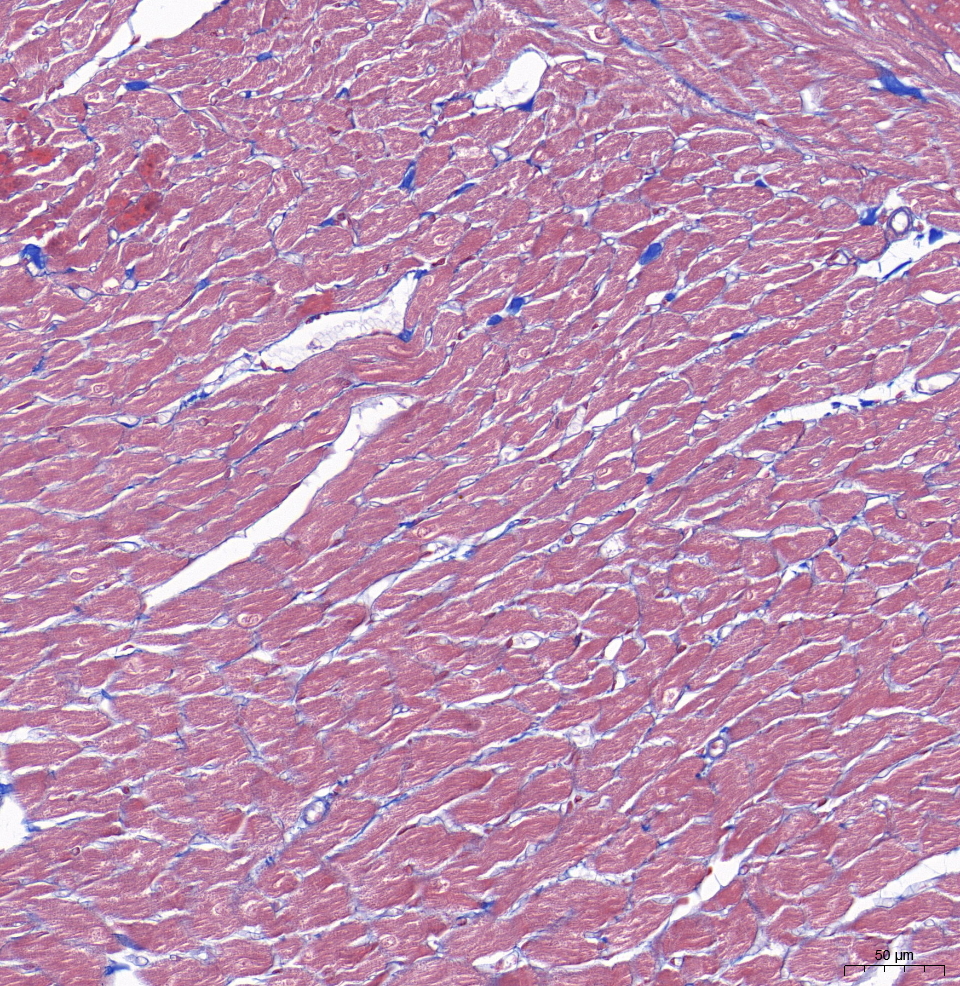

Supplement: S1 File — (ZIP) [file pone.0310136.s001.zip › Original data-GYJ-20240628/File 4. The original images of Masson staining/Figure_4/DE-interstitial fibrosis-A_20.0x.jpg]

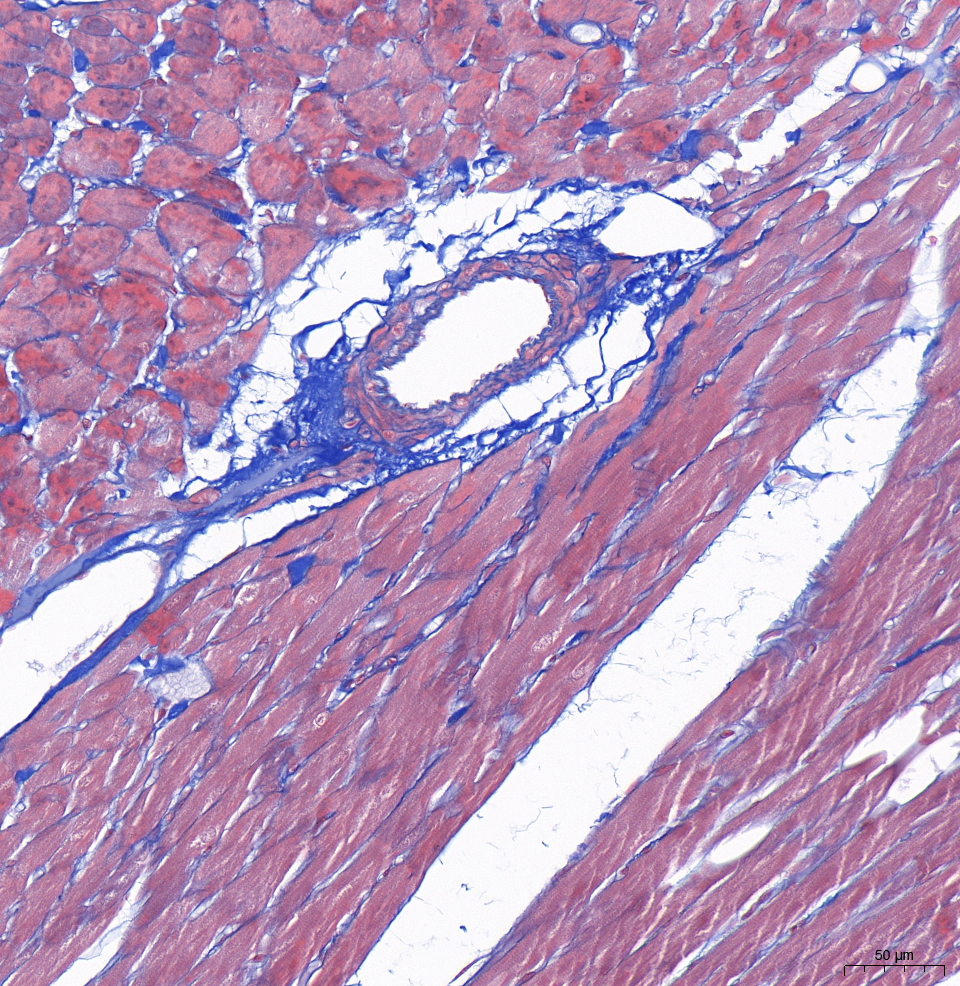

Supplement: S1 File — (ZIP) [file pone.0310136.s001.zip › Original data-GYJ-20240628/File 4. The original images of Masson staining/Figure_4/DI-Perivascular fibrosis 7512_20.0x.jpg]

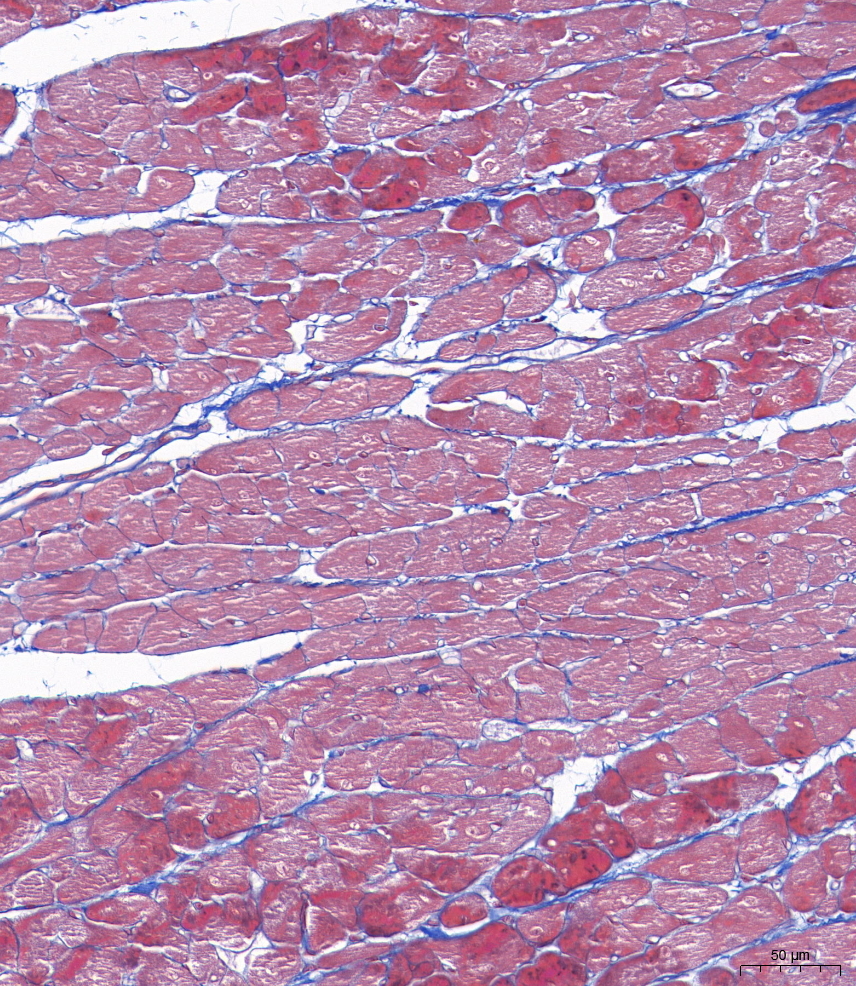

Supplement: S1 File — (ZIP) [file pone.0310136.s001.zip › Original data-GYJ-20240628/File 4. The original images of Masson staining/Figure_4/DI-interstitial fibrosis- 7512_20.0x 3.jpg]

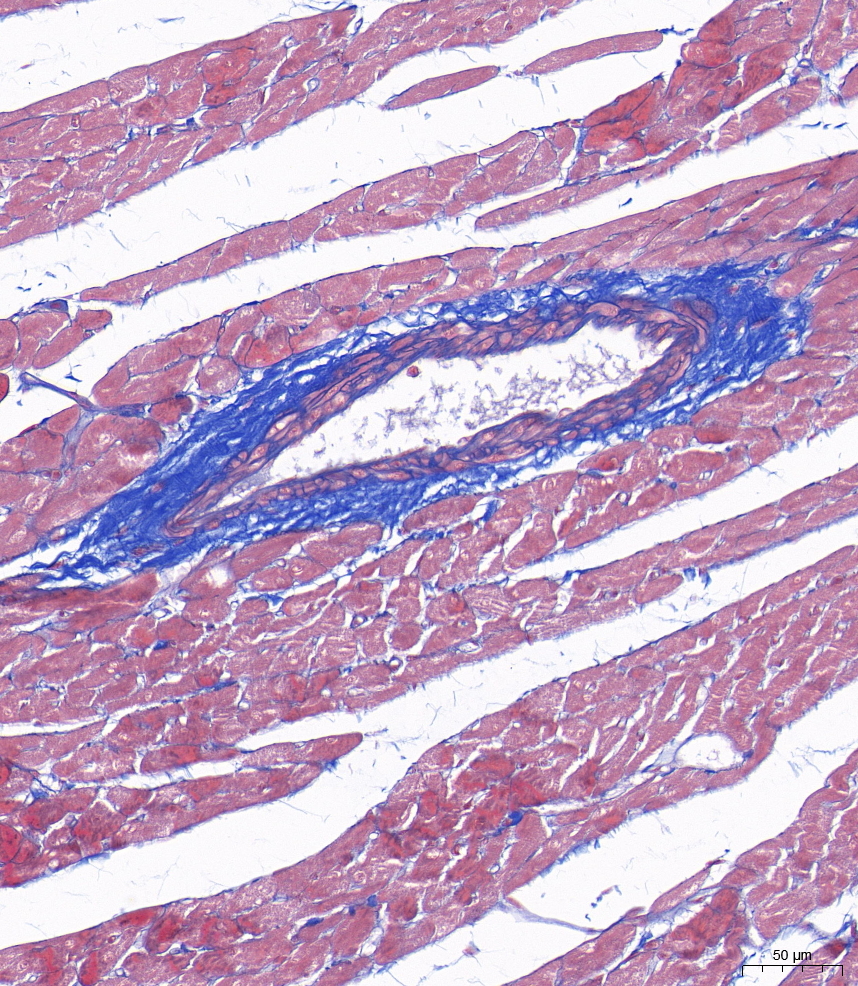

Supplement: S1 File — (ZIP) [file pone.0310136.s001.zip › Original data-GYJ-20240628/File 4. The original images of Masson staining/Figure_4/DM-Perivascular fibrosis_20.0x 2.jpg]

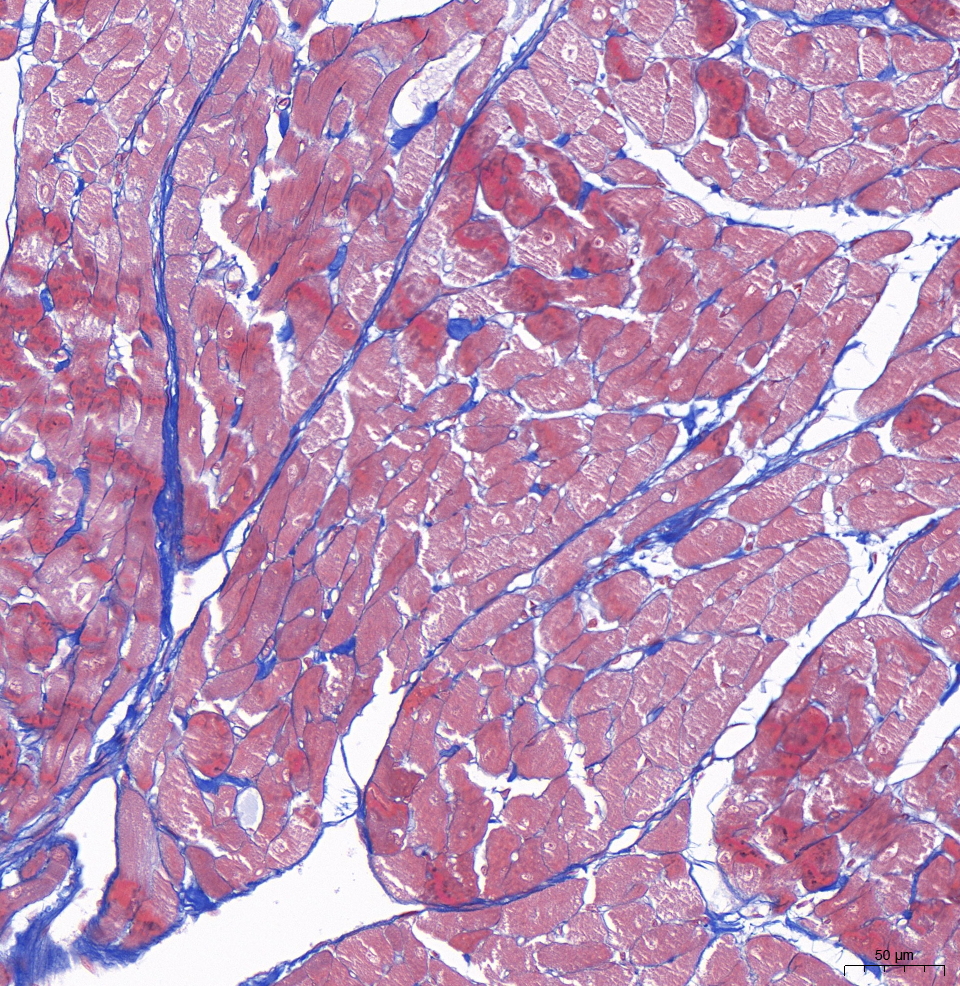

Supplement: S1 File — (ZIP) [file pone.0310136.s001.zip › Original data-GYJ-20240628/File 4. The original images of Masson staining/Figure_4/DM-interstitial fibrosis_20.0x.jpg]

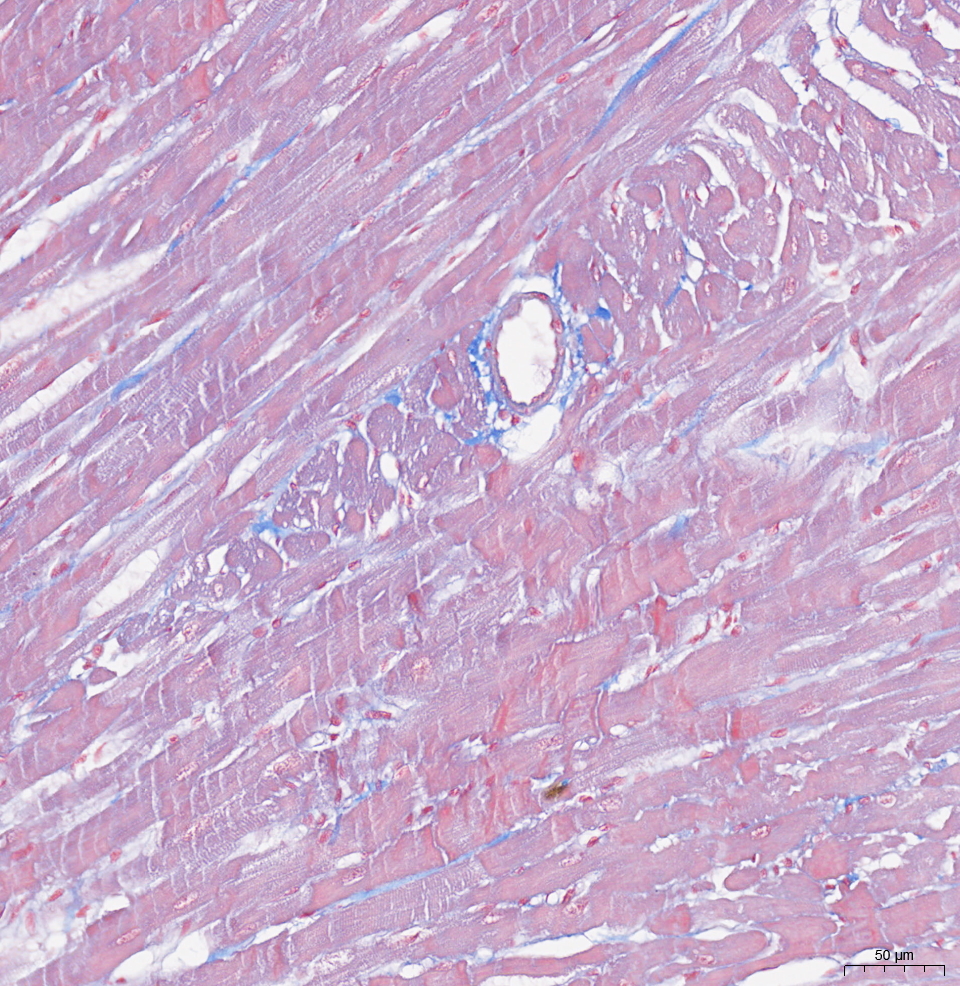

Supplement: S1 File — (ZIP) [file pone.0310136.s001.zip › Original data-GYJ-20240628/File 4. The original images of Masson staining/Figure_4/NC-Perivascular fibrosis 6.17.svs_20.0x 5.jpg]

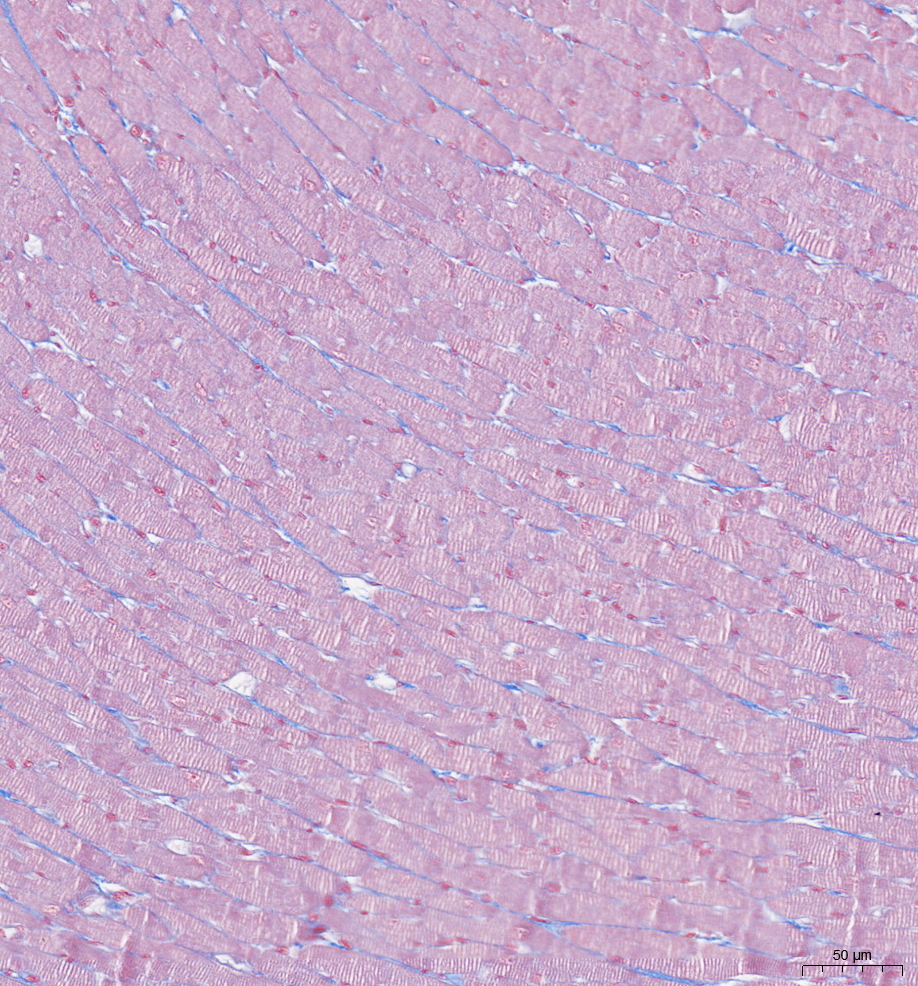

Supplement: S1 File — (ZIP) [file pone.0310136.s001.zip › Original data-GYJ-20240628/File 4. The original images of Masson staining/Figure_4/NC-interstitial fibrosis 6.17.svs_20.0x 1.jpg]

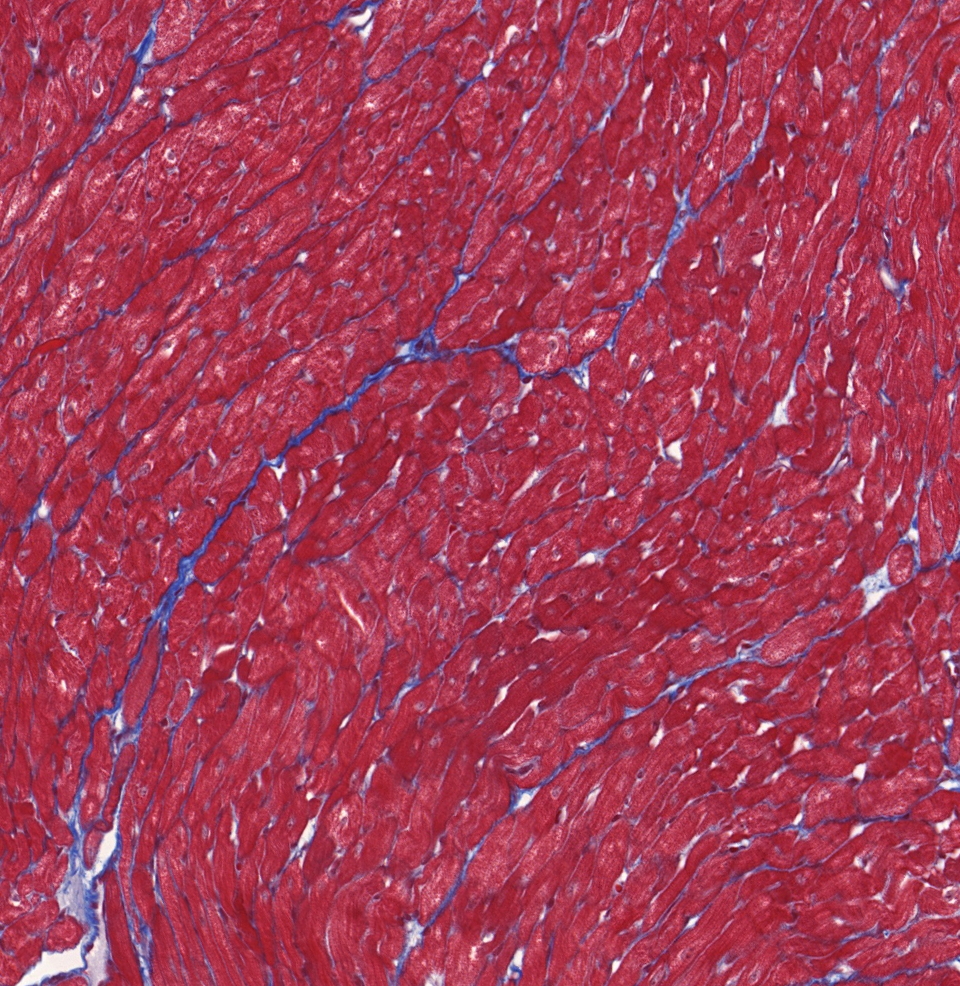

Supplement: S1 File — (ZIP) [file pone.0310136.s001.zip › Original data-GYJ-20240628/File 4. The original images of Masson staining/Figure_8/Antagomir-34a -interstitial fibrosis_20.0x.jpg]

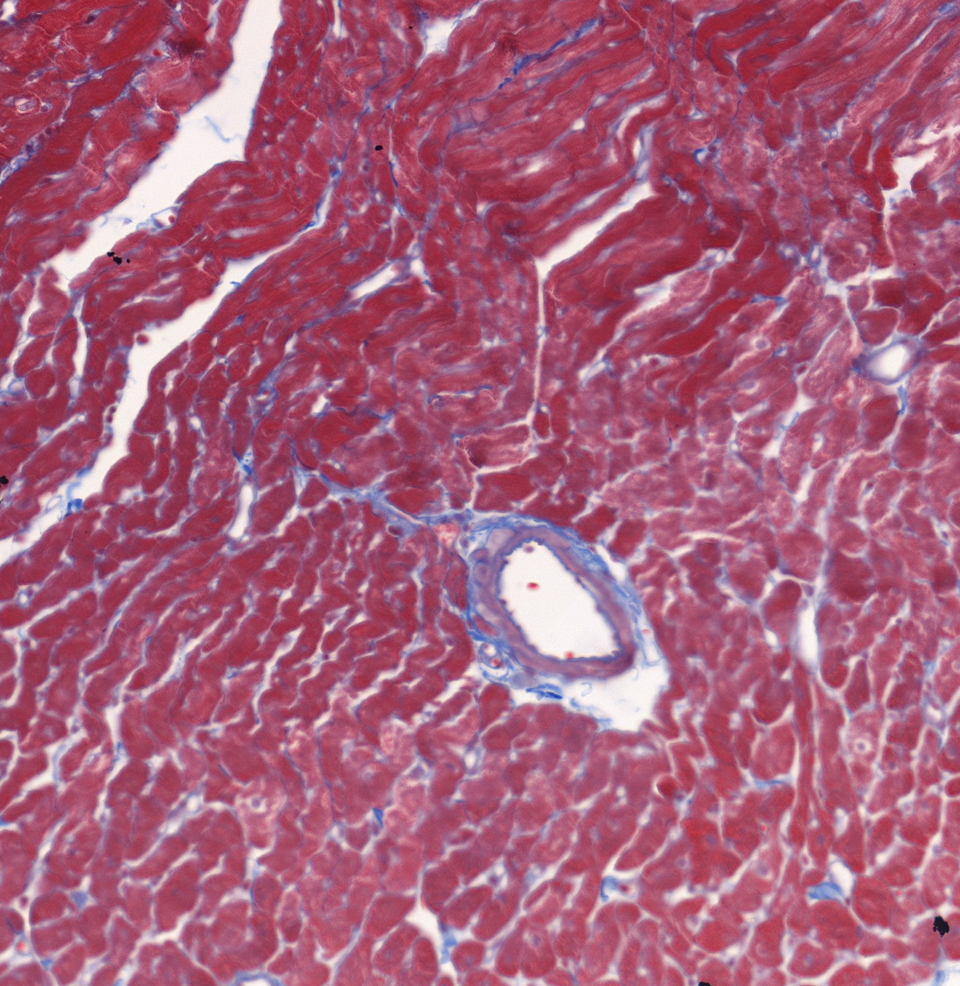

Supplement: S1 File — (ZIP) [file pone.0310136.s001.zip › Original data-GYJ-20240628/File 4. The original images of Masson staining/Figure_8/Antagomir-34a-Perivascular fibrosis-MA-_20.0x.jpg]

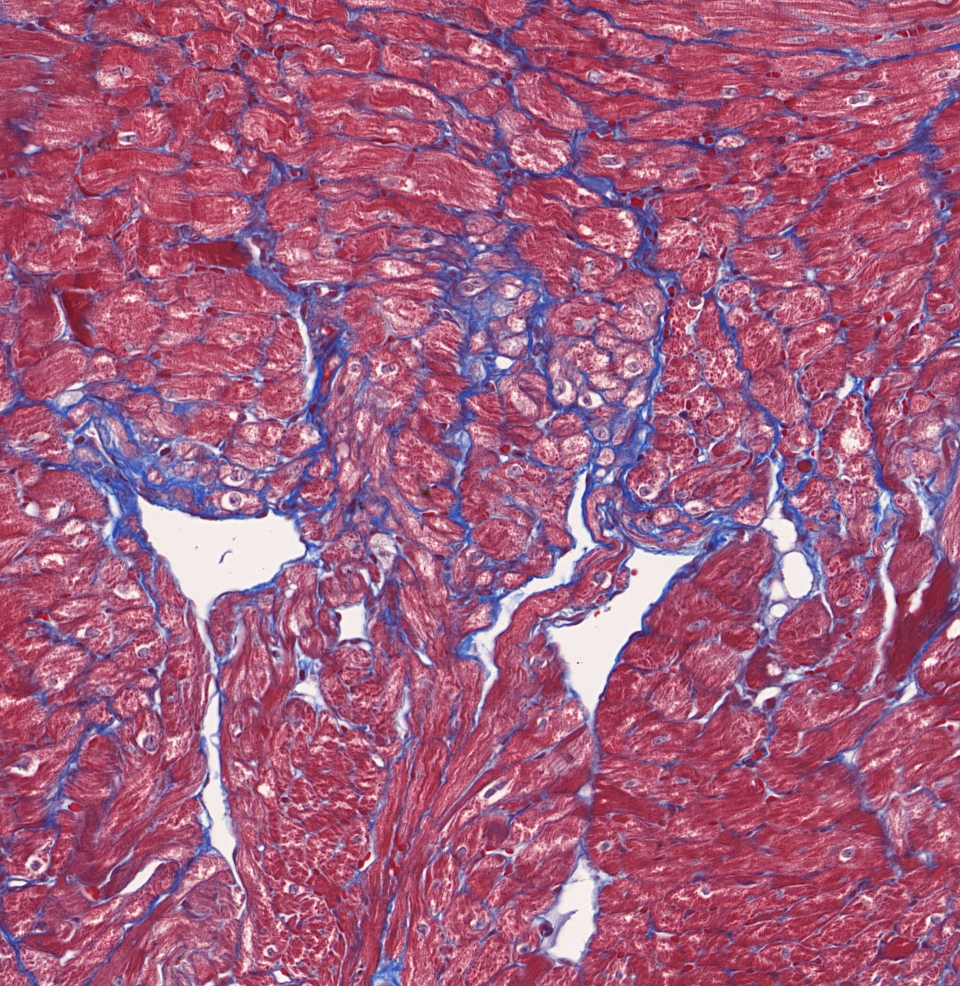

Supplement: S1 File — (ZIP) [file pone.0310136.s001.zip › Original data-GYJ-20240628/File 4. The original images of Masson staining/Figure_8/Antagomir-neg -interstitial fibrosis-A_20.0x.jpg]

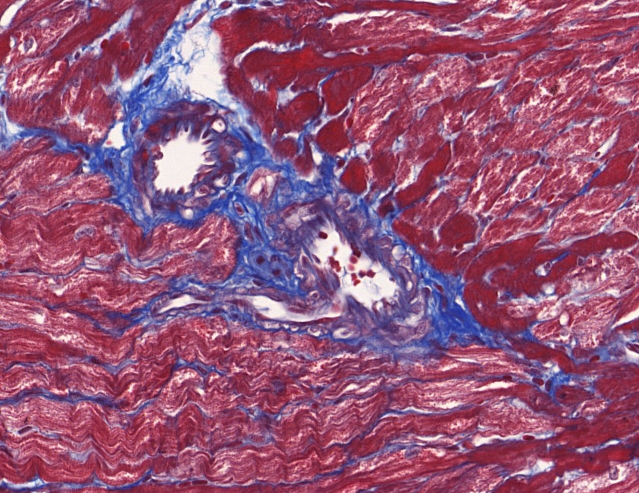

Supplement: S1 File — (ZIP) [file pone.0310136.s001.zip › Original data-GYJ-20240628/File 4. The original images of Masson staining/Figure_8/Antagomir-neg-Perivascular fibrosis-MA-_20.0x.jpg]

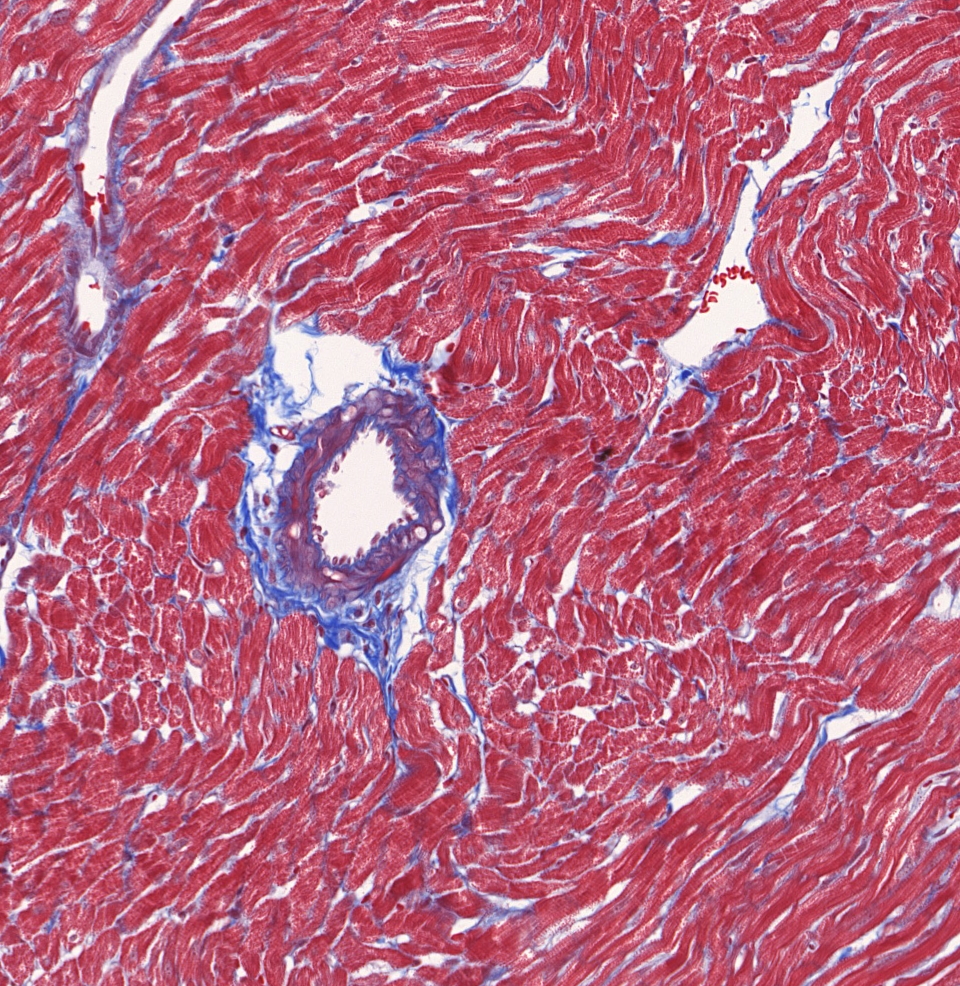

Supplement: S1 File — (ZIP) [file pone.0310136.s001.zip › Original data-GYJ-20240628/File 4. The original images of Masson staining/Figure_8/DE-Perivascular fibrosis-MA-20.0x.jpg]

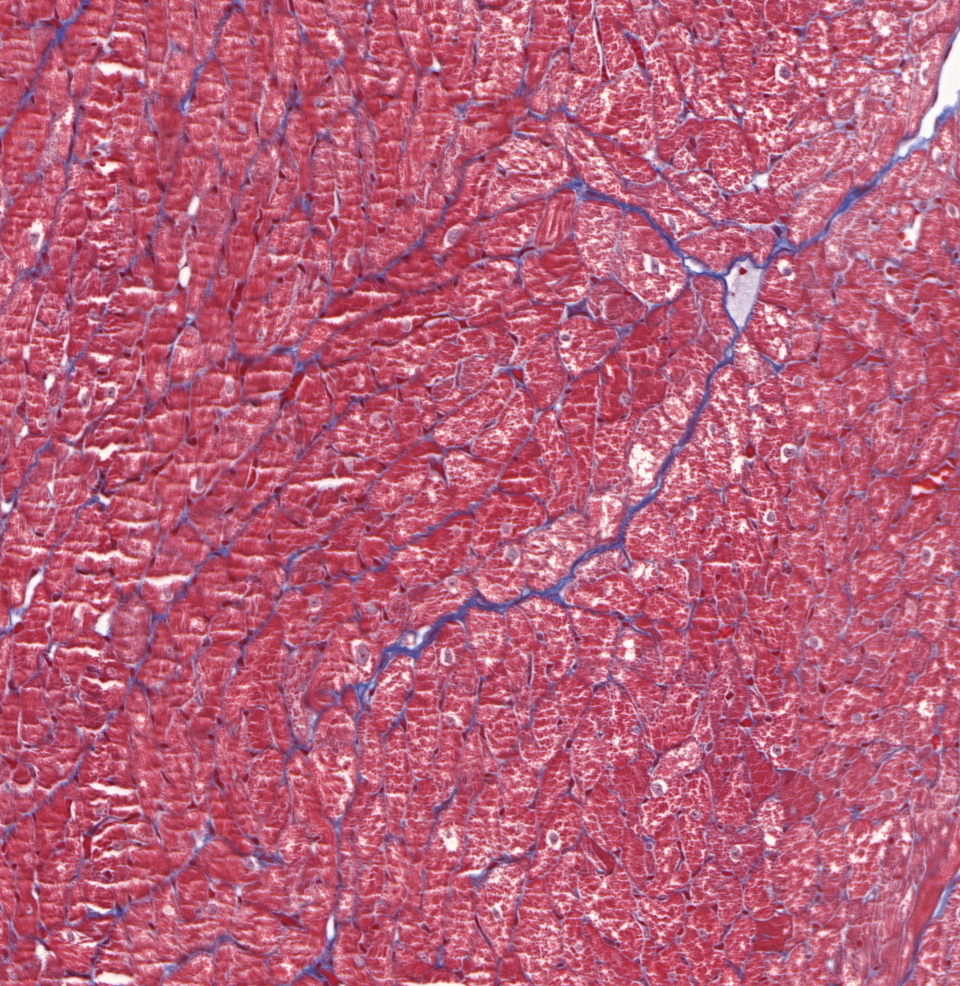

Supplement: S1 File — (ZIP) [file pone.0310136.s001.zip › Original data-GYJ-20240628/File 4. The original images of Masson staining/Figure_8/DE-interstitial fibrosis-C_20.0x.jpg]

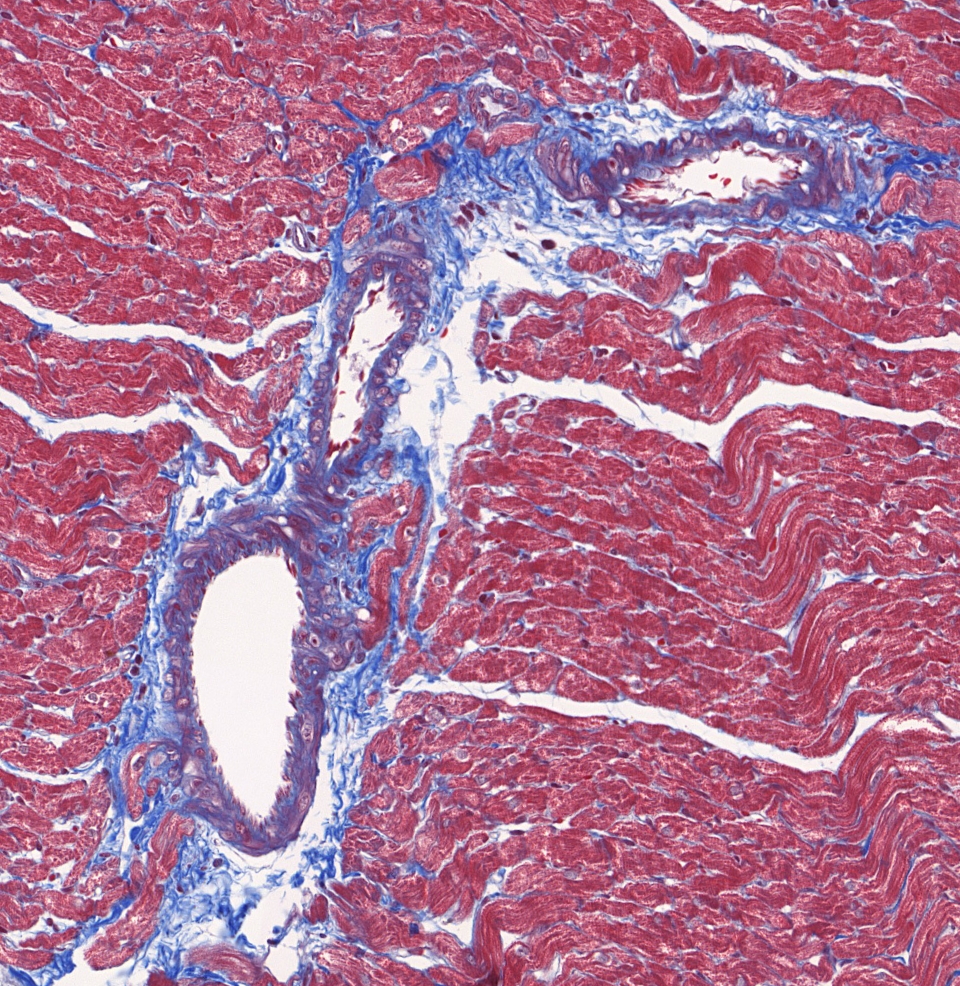

Supplement: S1 File — (ZIP) [file pone.0310136.s001.zip › Original data-GYJ-20240628/File 4. The original images of Masson staining/Figure_8/DM-Perivascular fibrosis-MA_20.0x.jpg]

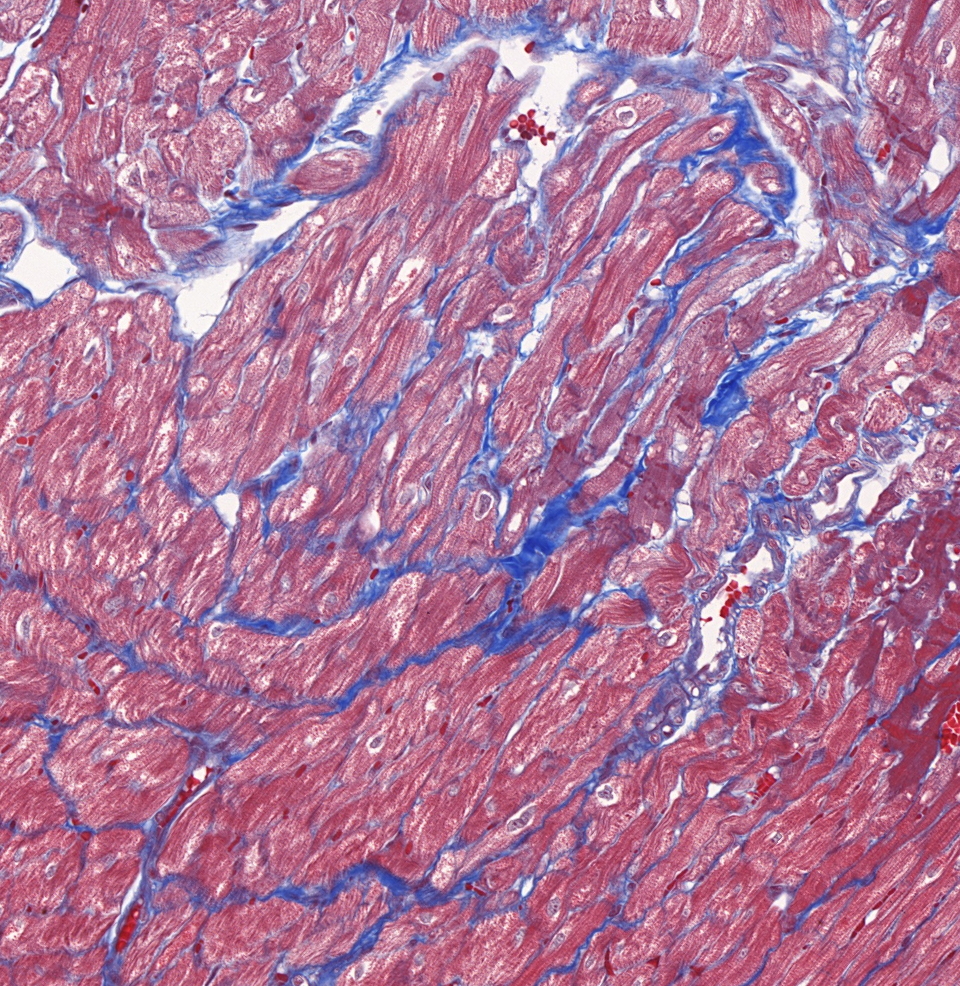

Supplement: S1 File — (ZIP) [file pone.0310136.s001.zip › Original data-GYJ-20240628/File 4. The original images of Masson staining/Figure_8/DM-interstitial fibrosis-B_20.0x.jpg]

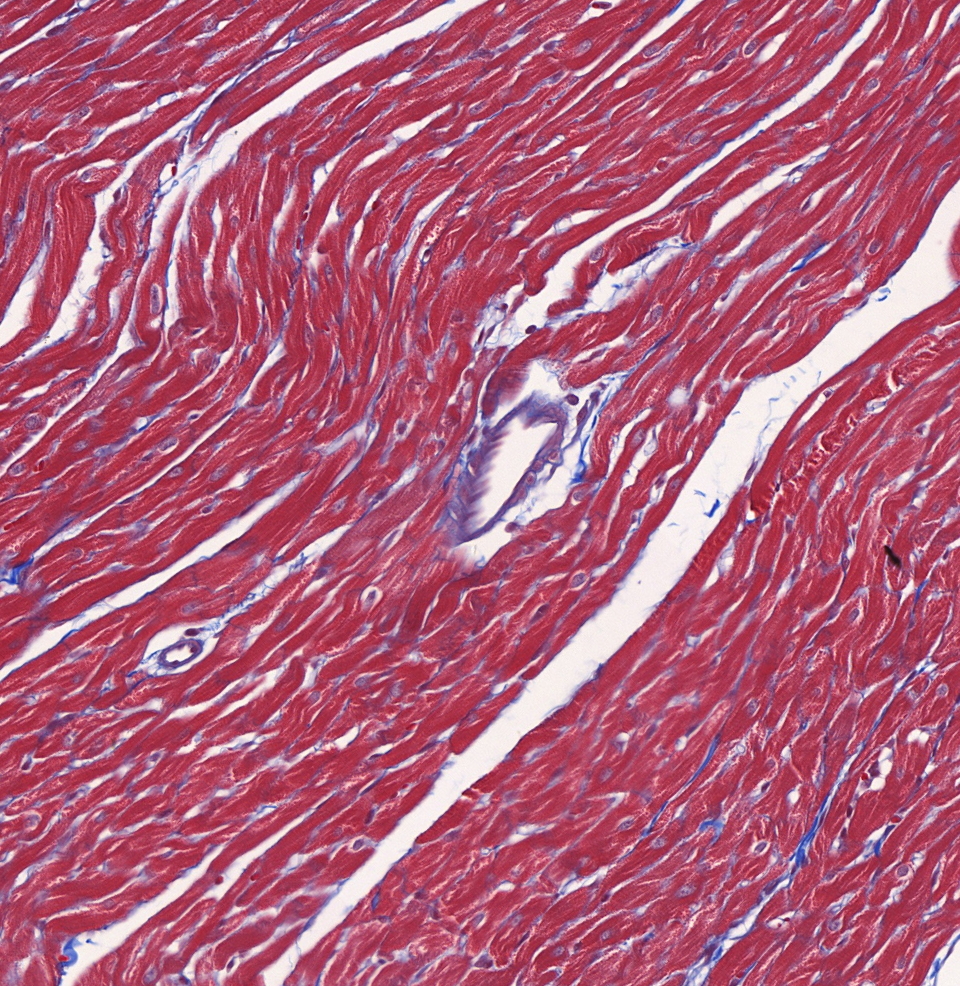

Supplement: S1 File — (ZIP) [file pone.0310136.s001.zip › Original data-GYJ-20240628/File 4. The original images of Masson staining/Figure_8/NC-Perivascular fibrosis-MA_20.0x.jpg]

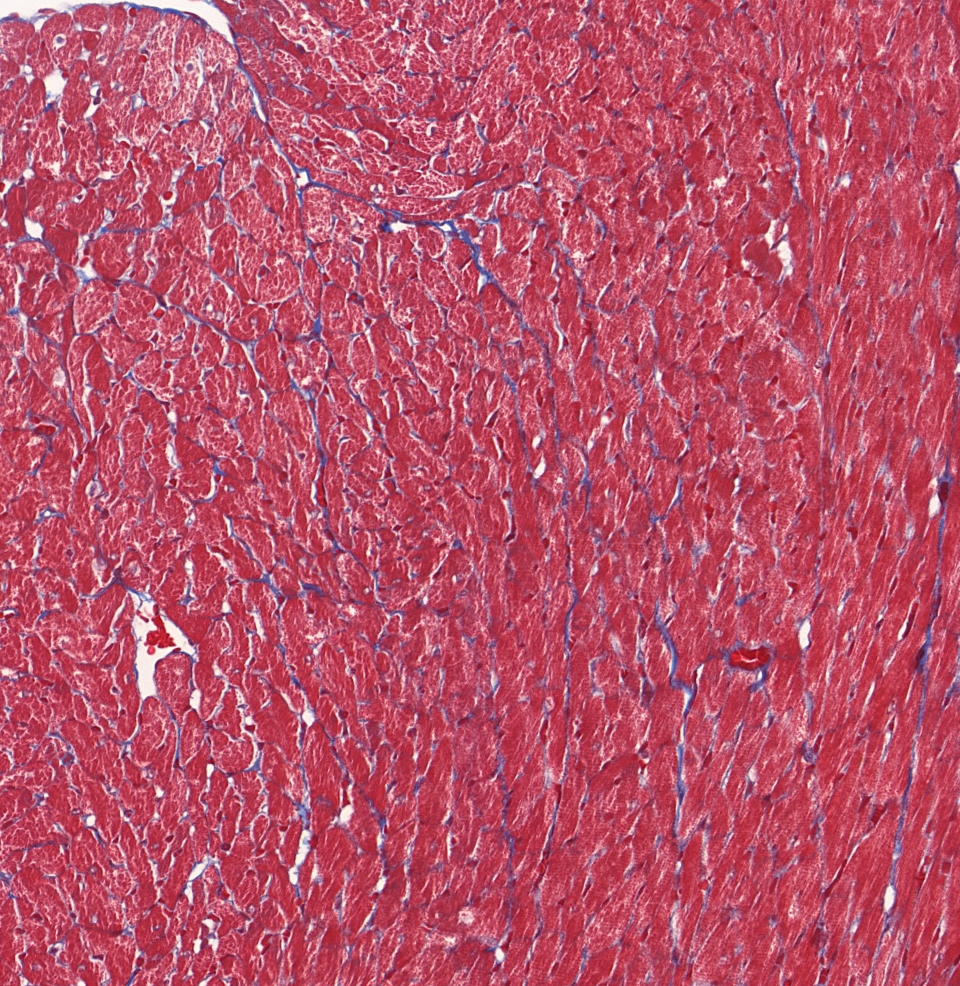

Supplement: S1 File — (ZIP) [file pone.0310136.s001.zip › Original data-GYJ-20240628/File 4. The original images of Masson staining/Figure_8/NC-interstitial fibrosis-A_20.0x.jpg]

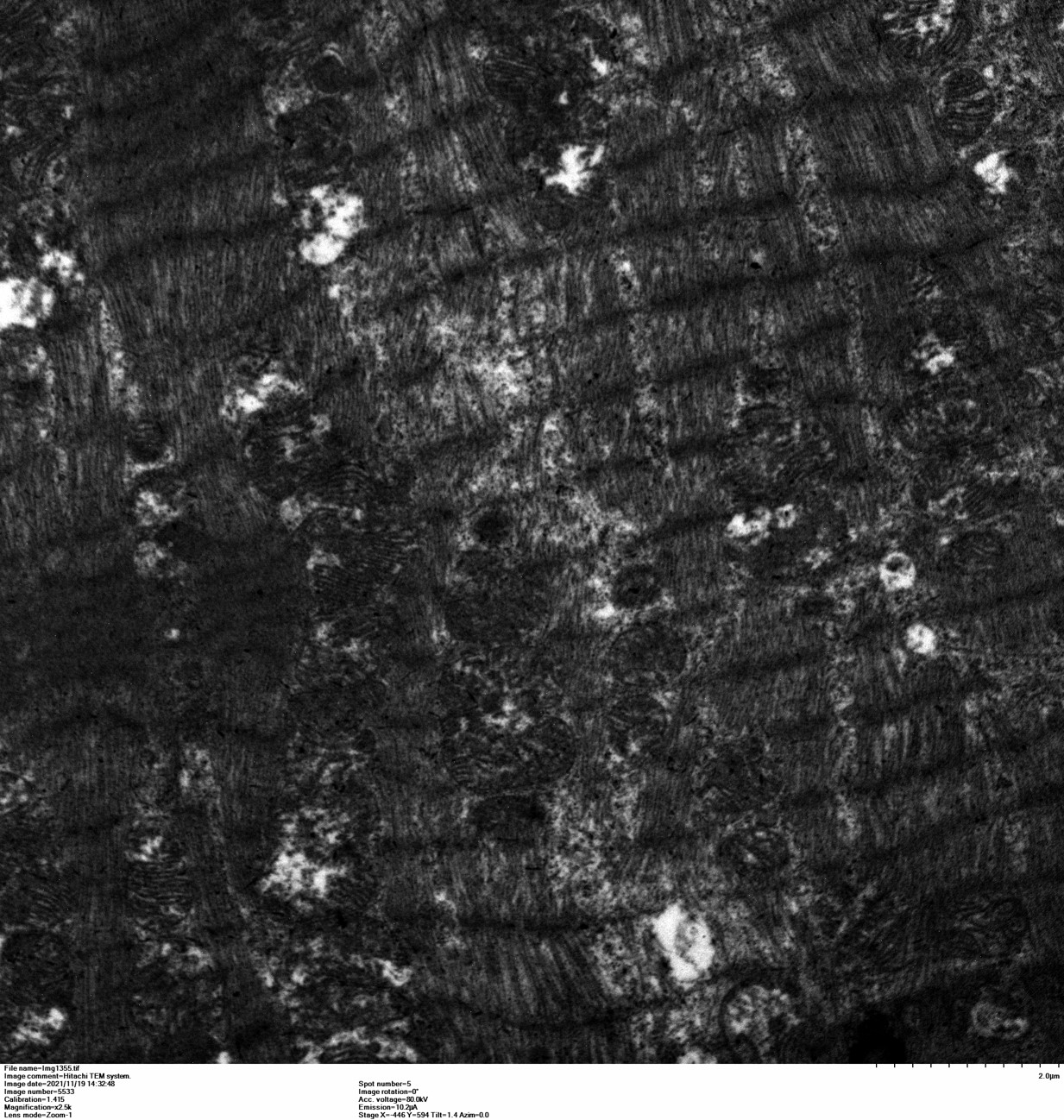

Supplement: S1 File — (ZIP) [file pone.0310136.s001.zip › Original data-GYJ-20240628/File 5. The original images of Transmission electron microscopy/Figure_4/DE-Img1355.jpg]

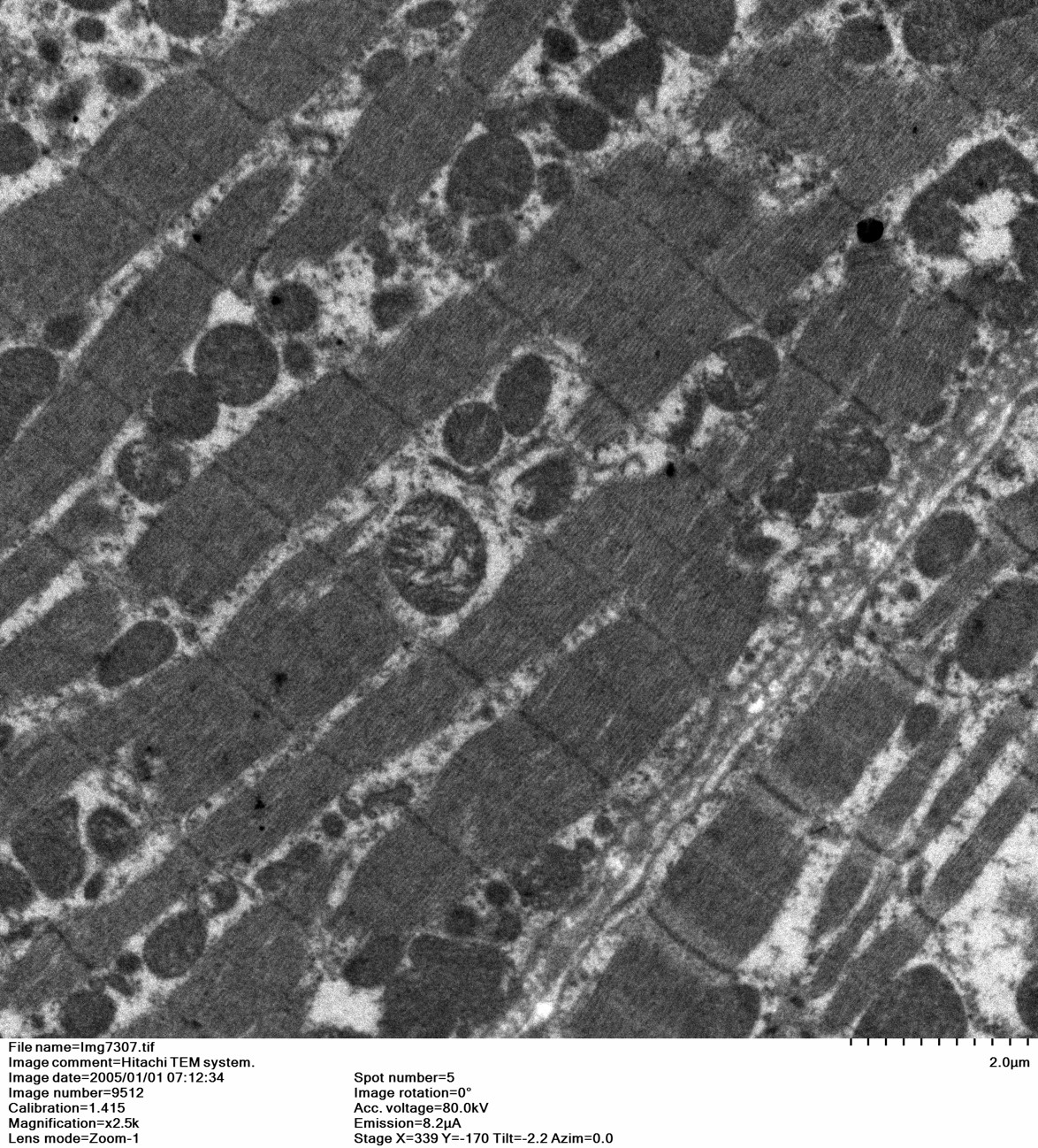

Supplement: S1 File — (ZIP) [file pone.0310136.s001.zip › Original data-GYJ-20240628/File 5. The original images of Transmission electron microscopy/Figure_4/DI-Img7307.jpg]

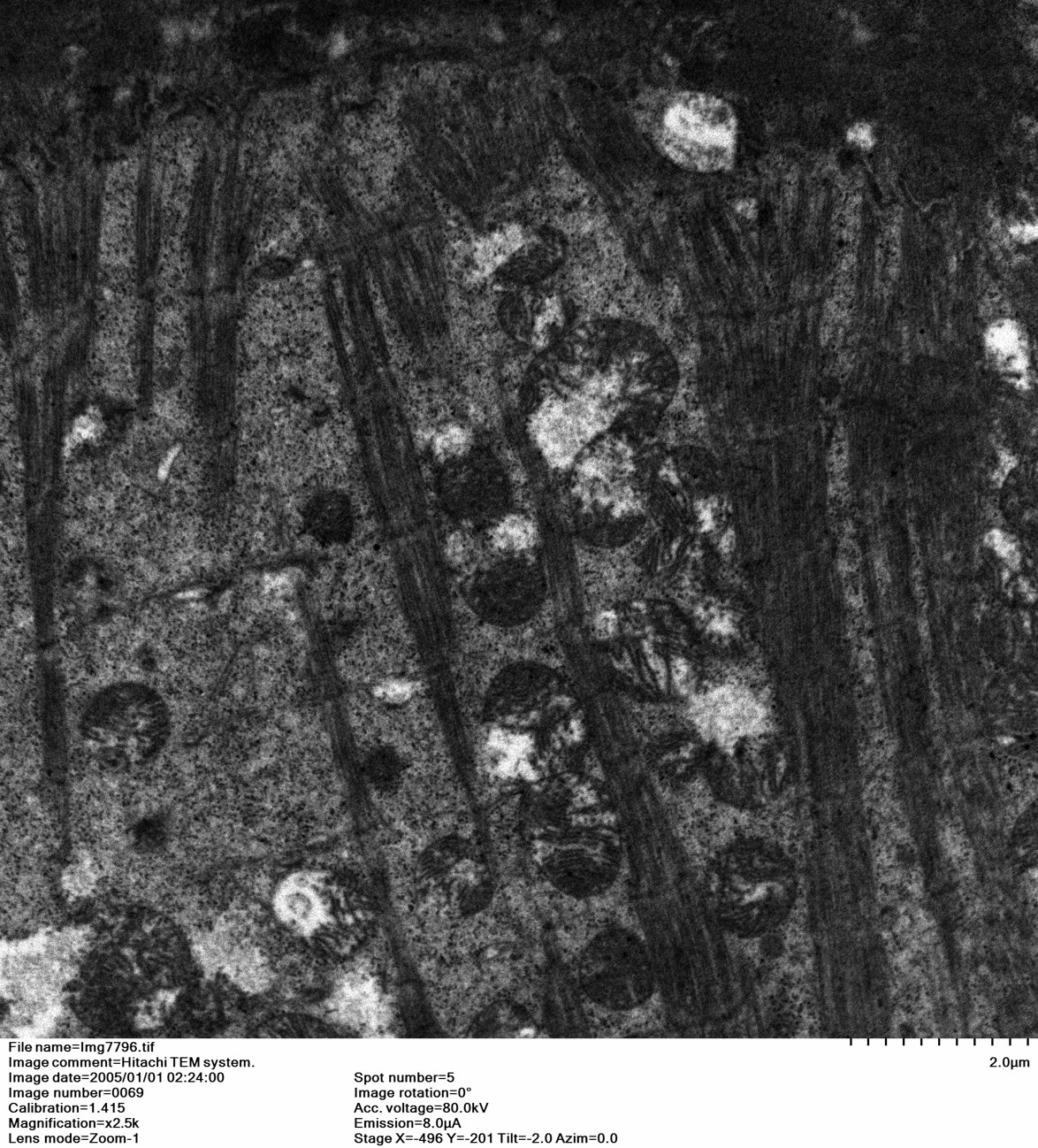

Supplement: S1 File — (ZIP) [file pone.0310136.s001.zip › Original data-GYJ-20240628/File 5. The original images of Transmission electron microscopy/Figure_4/DM-Img7796.jpg]

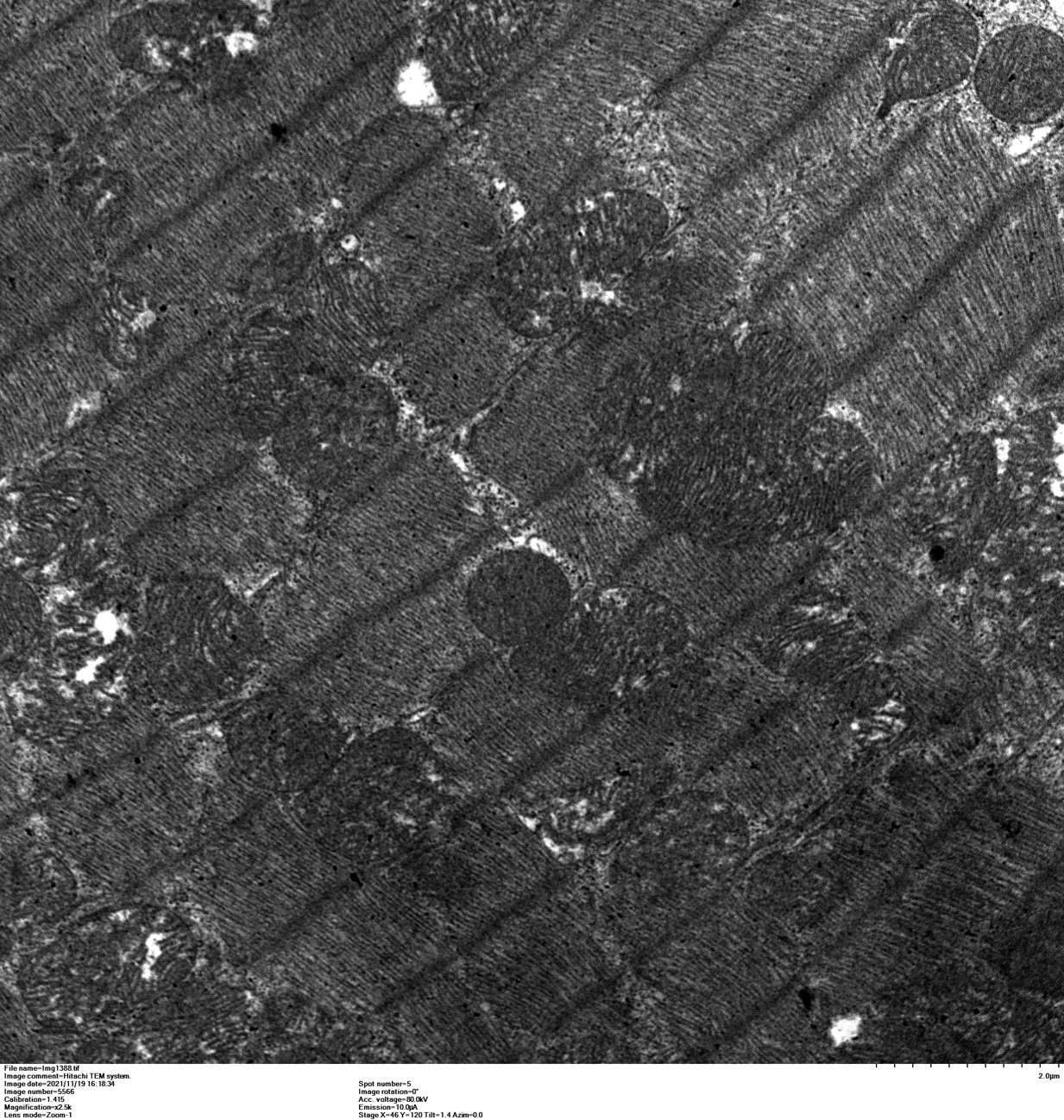

Supplement: S1 File — (ZIP) [file pone.0310136.s001.zip › Original data-GYJ-20240628/File 5. The original images of Transmission electron microscopy/Figure_4/NC-Img1388.jpg]

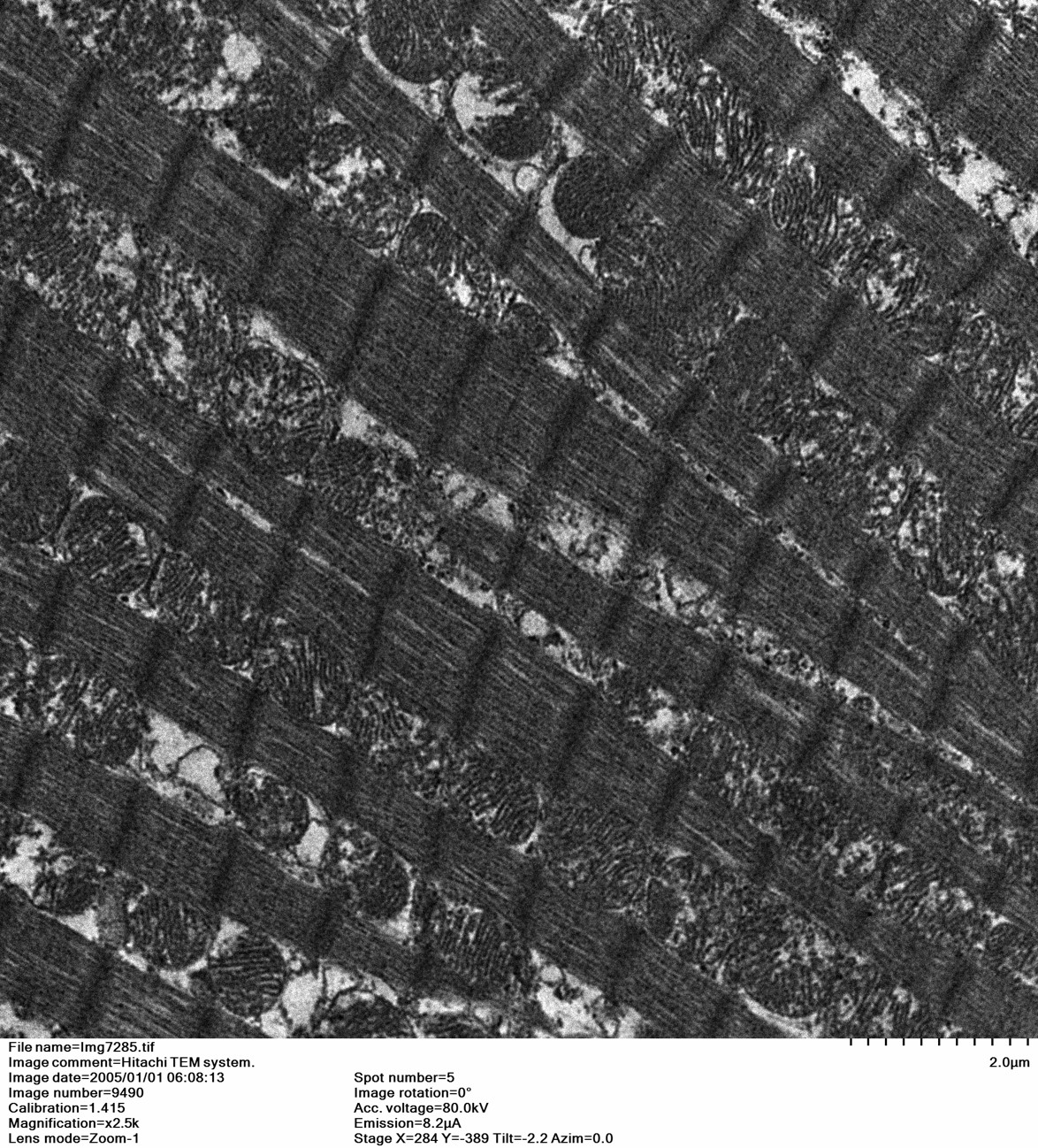

Supplement: S1 File — (ZIP) [file pone.0310136.s001.zip › Original data-GYJ-20240628/File 5. The original images of Transmission electron microscopy/Figure_8/Antagomir-34a Img7285.jpg]

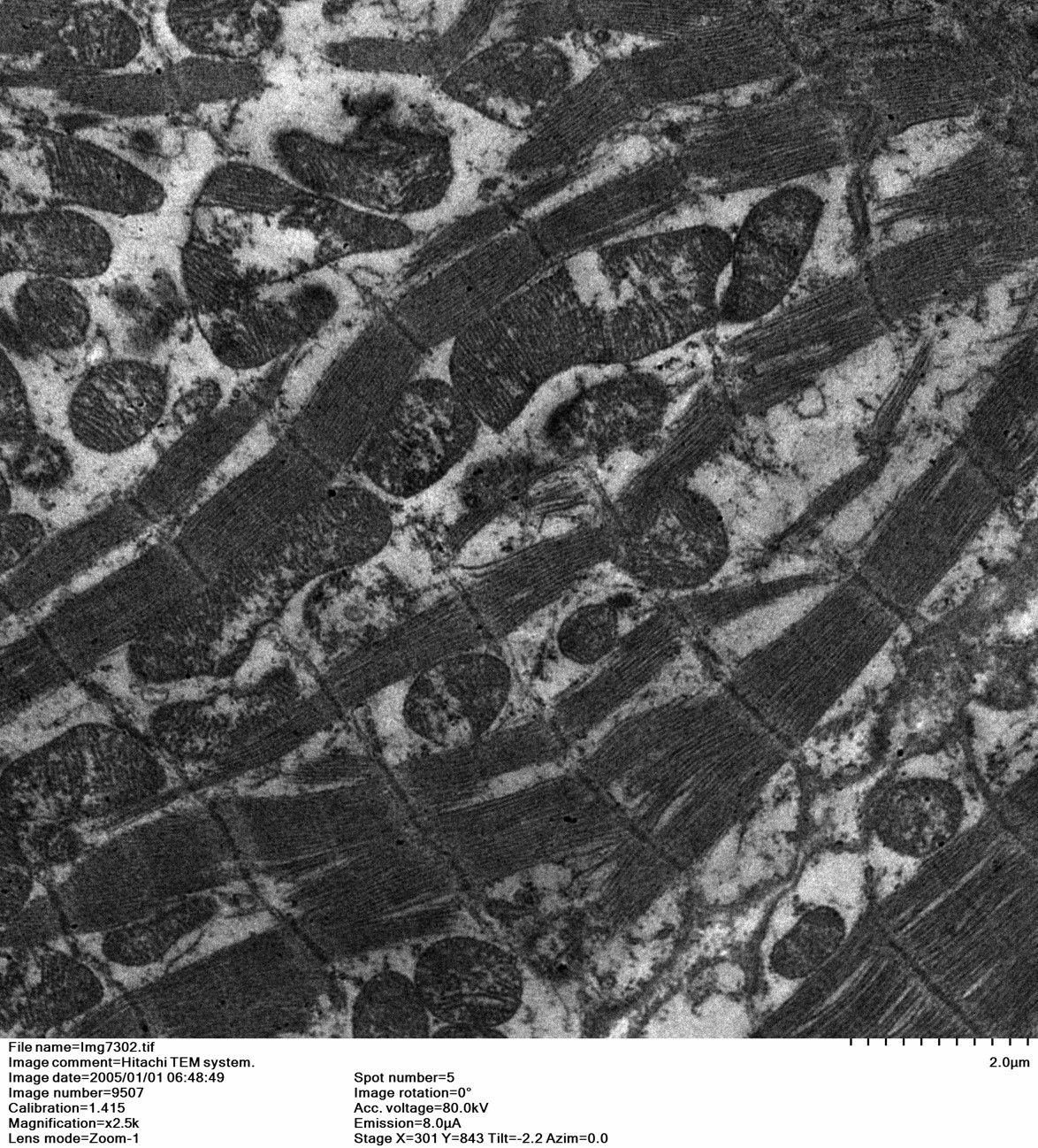

Supplement: S1 File — (ZIP) [file pone.0310136.s001.zip › Original data-GYJ-20240628/File 5. The original images of Transmission electron microscopy/Figure_8/Antagomir-neg Img7302.jpg]

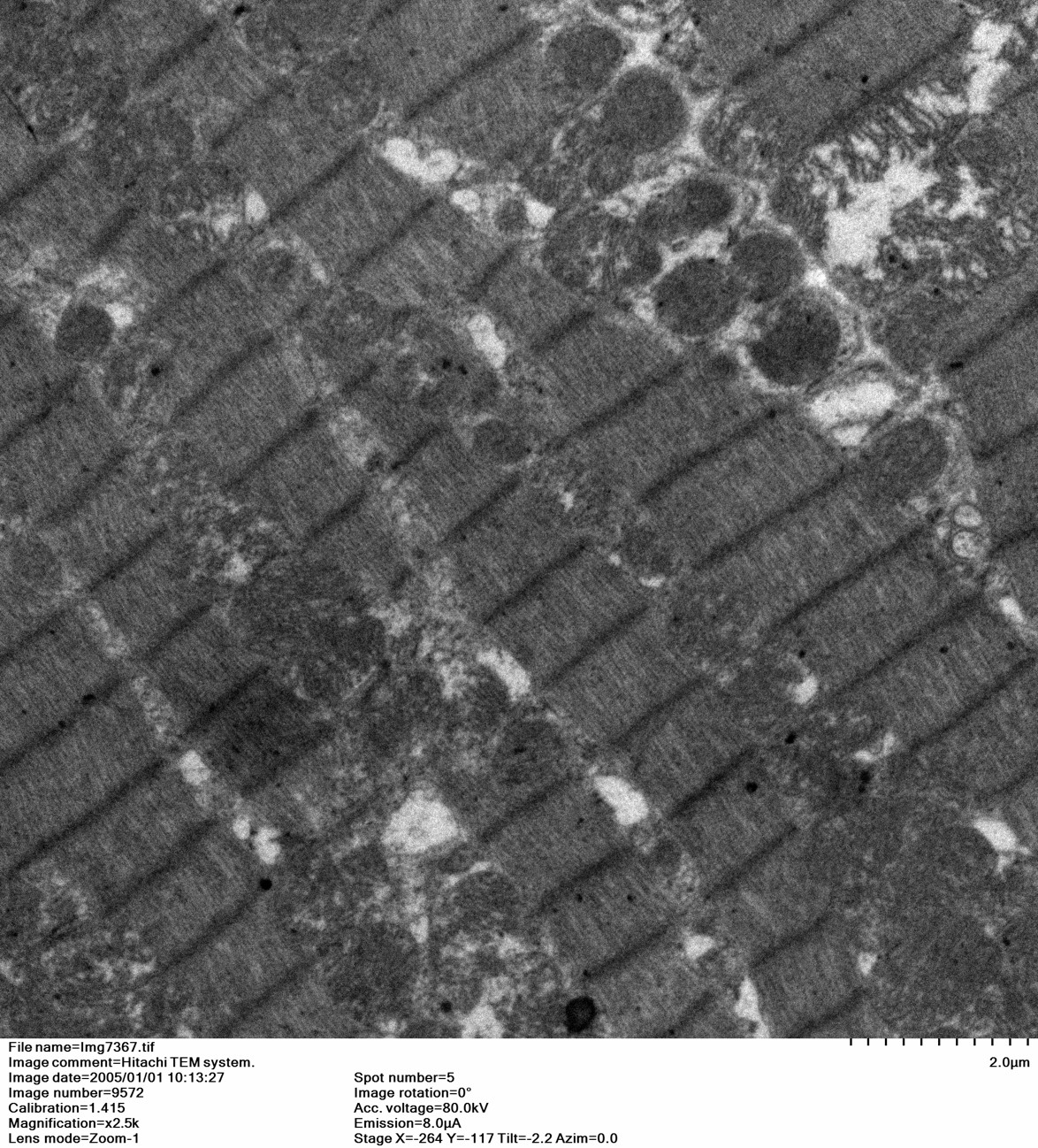

Supplement: S1 File — (ZIP) [file pone.0310136.s001.zip › Original data-GYJ-20240628/File 5. The original images of Transmission electron microscopy/Figure_8/DE-Img7367.jpg]

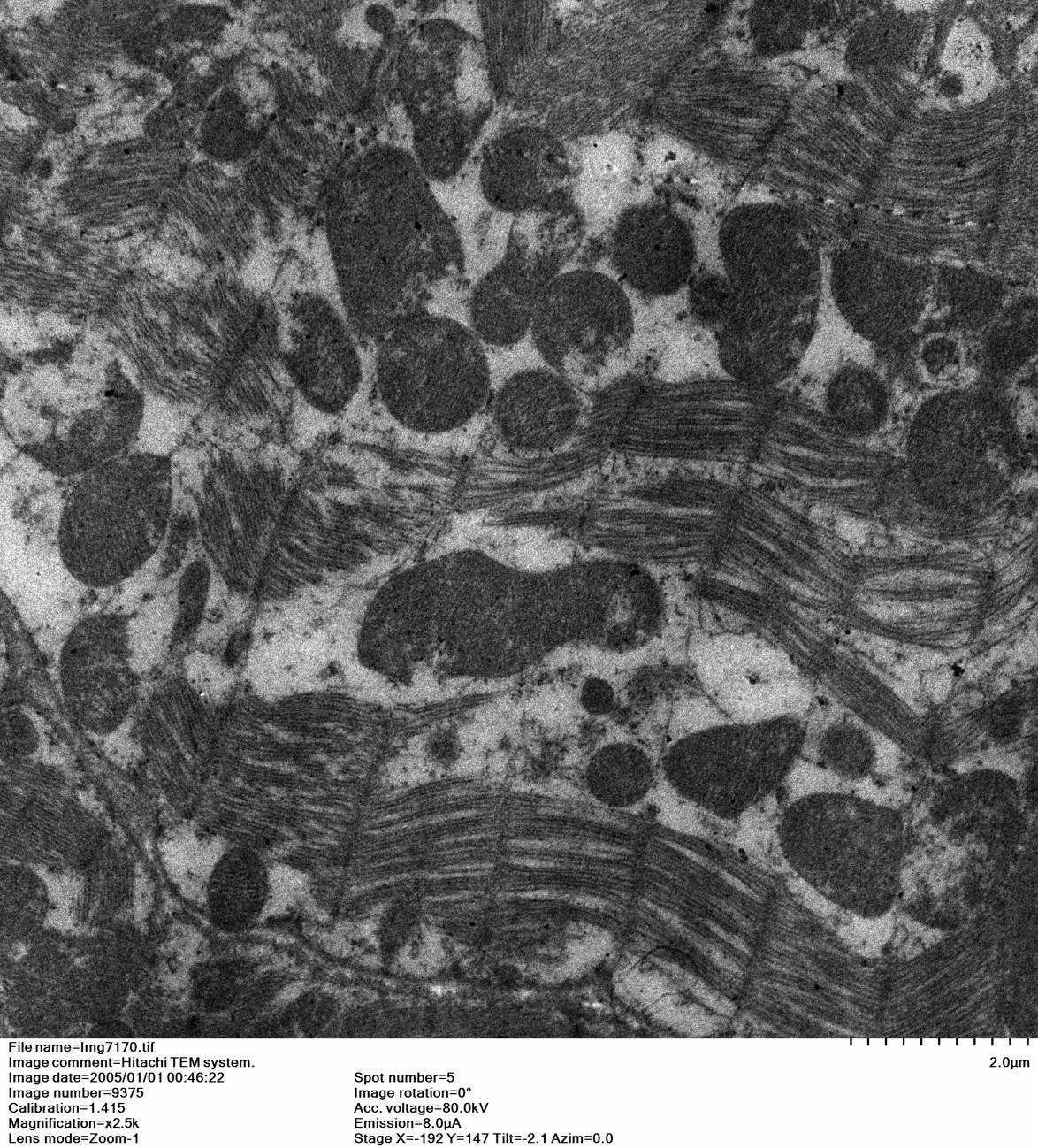

Supplement: S1 File — (ZIP) [file pone.0310136.s001.zip › Original data-GYJ-20240628/File 5. The original images of Transmission electron microscopy/Figure_8/DM-Img7170.jpg]

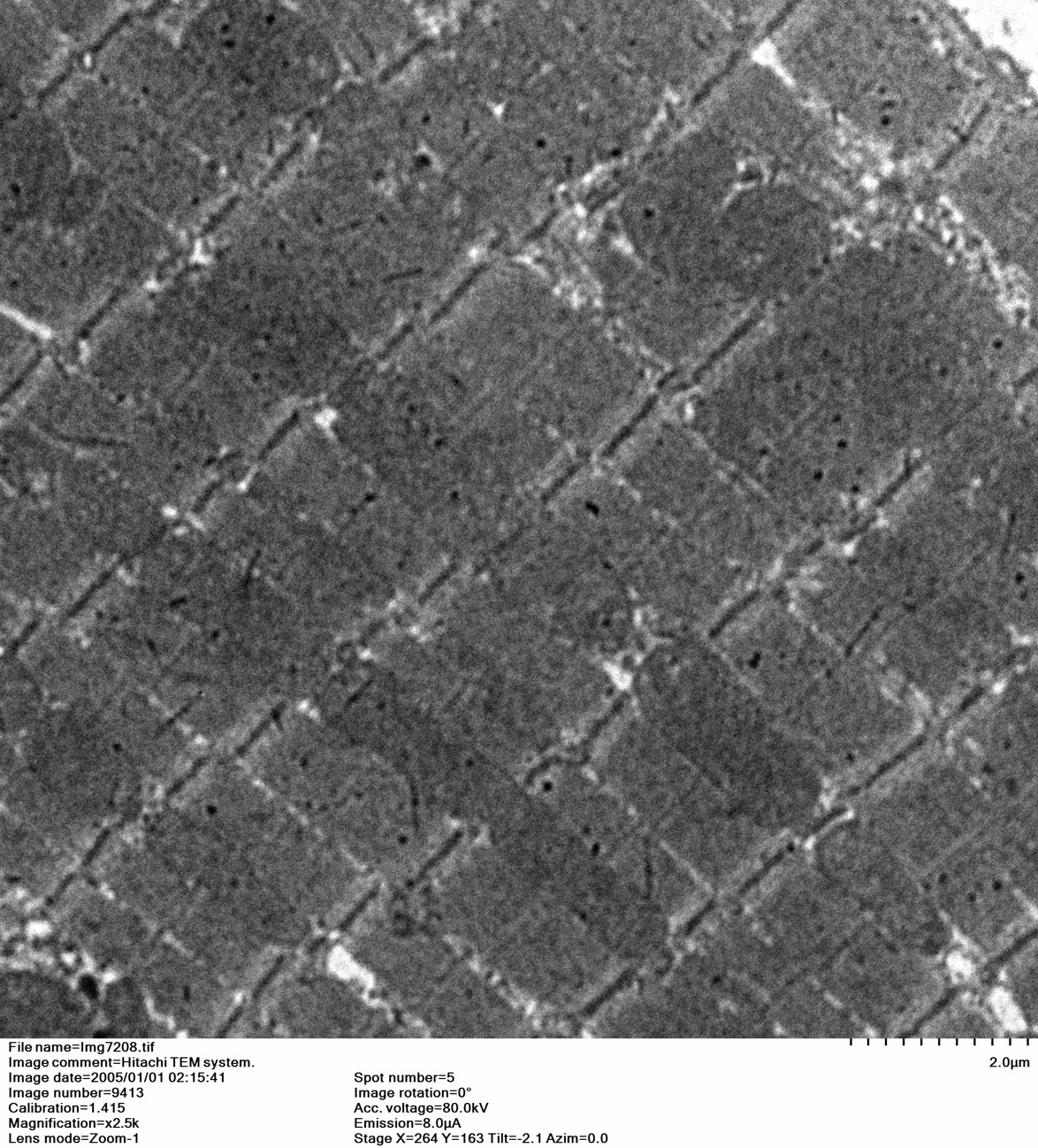

Supplement: S1 File — (ZIP) [file pone.0310136.s001.zip › Original data-GYJ-20240628/File 5. The original images of Transmission electron microscopy/Figure_8/NC-Img7208.jpg]

Figure 5 Original Western Blot images

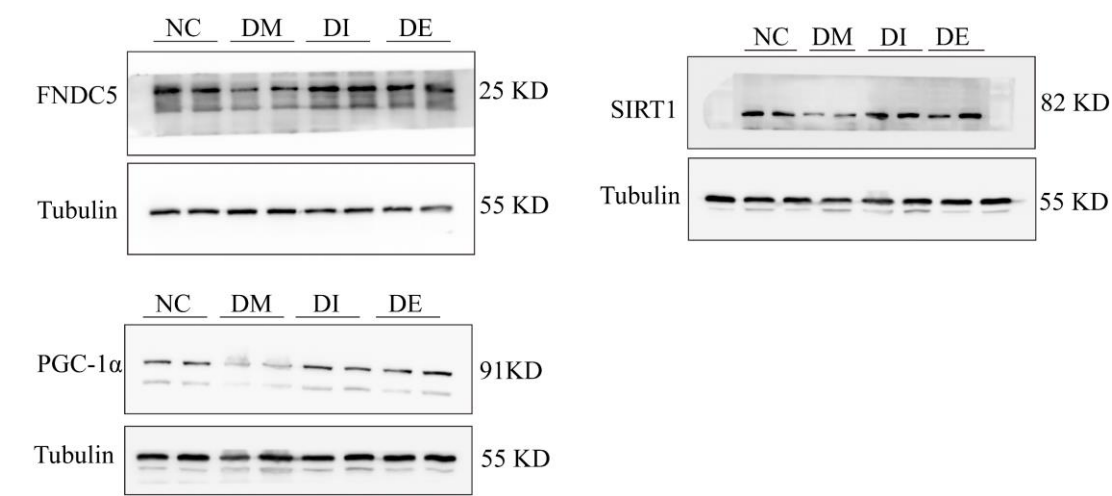

Figure 9 Original Western Blot images

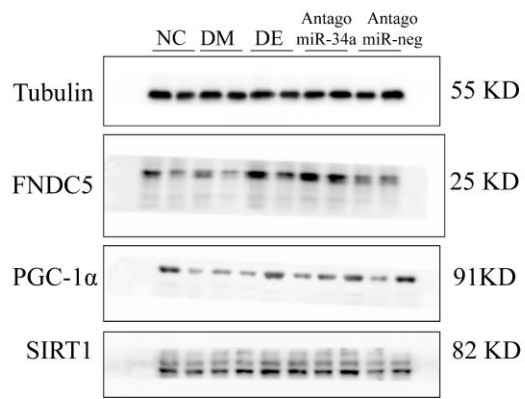

Supplement: S1 File — (ZIP) [file pone.0310136.s001.zip › Original data-GYJ-20240628/File 6. The original images of Western Bloting/The original western blot images of Figure 5 and Figure 9.pdf]
